# Transition Metal Carbonitride MXenes Anchored with Pt Sub-Nanometer Clusters to Achieve High-Performance Hydrogen Evolution Reaction at All pH Range

**DOI:** 10.1007/s40820-025-01654-y

**Published:** 2025-01-31

**Authors:** Zhihao Lei, Sajjad Ali, CI Sathish, MuhammadIbrar Ahmed, Jiangtao Qu, Rongkun Zheng, Shibo Xi, Xiaojiang Yu, M. B. H. Breese, Chao Liu, Jizhen Zhang, Shuai Qi, Xinwei Guan, Vibin Perumalsamy, Mohammed Fawaz, Jae-Hun Yang, Mohamed Bououdina, Kazunari Domen, Ajayan Vinu, Liang Qiao, Jiabao Yi

**Affiliations:** 1 Global Innovative Center of Advanced Nanomaterials, College of Engineering, Science and Environment, University of Newcastle, Callaghan, NSW 2308 Australia; 2 Energy, Water, and Environment Lab, College of Humanities and Sciences, Prince Sultan University, 11586 Riyadh, Saudi Arabia; 3 School of Physics, University of Sydney, Sydney, NSW 2000 Australia; 4 Institute of Chemical and Engineering Sciences, A*STAR, Singapore, 627833 Singapore; 5 Singapore Synchrotron Light Source, National University of Singapore, Singapore, 117603 Singapore; 6 Institute for Frontier Materials, Deakin University, Waurn Ponds, Victoria 3216 Australia; 7 Guangdong Provincial Key Laboratory of Natural Rubber Processing, Agricultural Products Processing Research Institute, Chinese Academy of Tropical Agricultural Sciences, Zhanjiang, 524001 People’s Republic of China; 8 College of Chemistry Environmental Engineering, Shenzhen University, Shenzhen, 518060 Guangdong People’s Republic of China; 9 Research Initiative for Supra-Materials Interdisciplinary Cluster for Cutting Edge Research, Shinshu University, 4-17-1, Wakasato, Nagano-shi, Nagano 380-8533 Japan; 10 School of Physics, University of Electronic Science and Technology of China, Chengdu, 610054 People’s Republic of China; 11 Department of Chemical Engineering and Interdisciplinary Research Center for Hydrogen Technologies and Carbon Management (IRC-HTCM), King Fahd University of Petroleum and Minerals, 31261 Dhahran, Saudi Arabia

**Keywords:** MXene, Hydrogen evolution reaction, Single atom, Two-dimensional nanosheets, Density functional theory

## Abstract

Transition metal carbides, known as MXenes, particularly Ti_3_C_2_T_x_, have been extensively explored as promising materials for electrochemical reactions. However, transition metal carbonitride MXenes with high nitrogen content for electrochemical reactions are rarely reported. In this work, transition metal carbonitride MXenes incorporated with Pt-based electrocatalysts, ranging from single atoms to sub-nanometer dimensions, are explored for hydrogen evolution reaction (HER). The fabricated Pt clusters/MXene catalyst exhibits superior HER performance compared to the single-atom-incorporated MXene and commercial Pt/C catalyst in both acidic and alkaline electrolytes. The optimized sample shows low overpotentials of 28, 65, and 154 mV at a current densities of 10, 100, and 500 mA cm^−2^, a small Tafel slope of 29 mV dec^−1^, a high mass activity of 1203 mA mg_Pt_^−1^ and an excellent turnover frequency of 6.1 s^−1^ in the acidic electrolyte. Density functional theory calculations indicate that this high performance can be attributed to the enhanced active sites, increased surface functional groups, faster charge transfer dynamics, and stronger electronic interaction between Pt and MXene, resulting in optimized hydrogen absorption/desorption toward better HER. This work demonstrates that MXenes with a high content of nitrogen may be promising candidates for various catalytic reactions by incorporating single atoms or clusters.

**Supplementary Information:**

The online version contains supplementary material available at 10.1007/s40820-025-01654-y.

## Highlights

- Two-dimensional mono- and few-layered Ti_3_CNT_x_ MXene nanosheets with extremely high nitrogen content were synthesized.
- Better performance for hydrogen evolution reaction (HER) than Pt/C catalyst in acidic, neutral and alkaline solutions.
- Exceptional performance of HER in both acidic and alkaline solutions.
- A large current density (> 500 mA cm^−2^) has been achieved for HER.

**Supplementary Information:**

The online version contains supplementary material available at 10.1007/s40820-025-01654-y.

## Graphical abstract

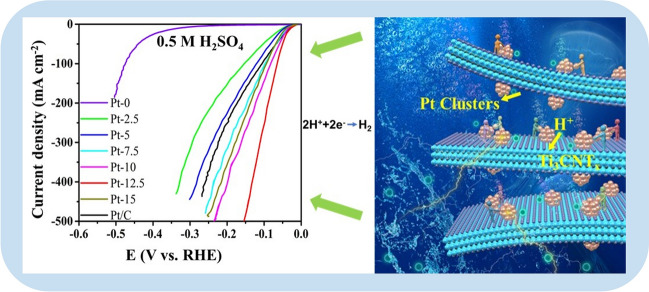

## Introduction

The persistent and intensifying climate issues, the ever-growing depletion of fossil fuels, and the massive greenhouse gas emissions demand the development of green and clean energy to maintain a healthy and sustainable living environment [1–4]. Hydrogen energy, which can be obtained from water, is one of the most promising candidates. In contrast, the efficiency of hydrogen production remains very low, and the associated cost is still pretty high. Therefore, searching for suitable catalysts to achieve a high hydrogen production rate is in demand [5–7]. Currently, platinum (Pt) is the benchmark catalyst for the hydrogen evolution reaction (HER) [8–10]. Traditional Pt-based catalysts have limited atom utilization efficiency, as only a small fraction of Pt atoms is actively involved in catalysis. Moreover, the scarcity and high cost of Pt limit its viability as a long-term and sustainable choice. To realize both high efficiency and maximum atom utilization, rational catalyst design strategies like reducing the dosage and size of Pt are considered promising ways to overcome the dilemma. However, when Pt is downsized to sub-nanometer clusters or single atoms, it is extremely important to search for suitable substrates to accommodate them with strong interactions to avoid surface diffusion and coarsening under applied bias. This could improve the intrinsic activity and stability of the active site, which is a key factor in HER performance.

MXenes, first discovered in 2011, are considered an important substrate to stabilize noble metal atoms due to their intrinsic high surface area, hydrophilicity, diverse redox active sites, and surface functionalities [11–13]. The high surface area provides a platform for enhanced active site density distribution. Moreover, their hydrophilic nature and surface functional groups improve the interaction of metal moieties with the MXene, which results in electronic structure modulation with improved intrinsic catalytic activity [14–16]. As a new type of 2D metal carbides or nitrides, most MXenes were obtained from the parent MAX materials via typical strategies like acid etching [17] and molten-salt etching [18]. Notably, during the acid etching process, defects such as metal and oxygen vacancies are generally formed on the surface of MXenes, which can serve as anchoring sites to capture noble metal atoms/clusters [19–21]. Therefore, MXene is considered an electrocatalytic substrate with low cost and great potential in electrochemical processes. For example, noble-metals-incorporated MXenes have demonstrated excellent HER performance [22–25]. It is well known that nitrogen doping is one of the best strategies for improving materials’ electrochemical activities, such as N doped carbon, graphene or carbon nitride with high content of nitrogen [7, 26, 27]. Similarly, nitrogen-doped MXenes also demonstrated enhanced electrochemical activities [24, 28–32], which is because the presence of N provides localized charge density sites due to the polarizability caused by the electronegative N atoms, thus improving the electronic structure and electrochemical kinetics [33]. From theoretical calculations, Le et al. observed a lower Gibbs free energy on Ti_3_N_x_C with 50% of N content (–0.029 eV) than the Pt (111) plane (–0.09 eV) for hydrogen adsorption [34]. Nitrogen doping has also improved the electrochemical properties of MXenes in other electrochemical reactions besides HER [35, 36].

Herein, we report the synthesis of 2D Ti_3_CNT_x_ MXene nanosheets through a mild etching strategy and employ them as the conductive support to anchor Pt single atoms and sub-nanometer clusters. Due to the vacancies in Ti and other atoms induced by etching, the Pt species are spontaneously reduced on the vacancy sites of MXene and stabilized by the connections with the surrounding –N and –O terminations. The as-produced Pt-MXene catalyst with sub-nanometer Pt clusters shows exceptional HER performance with a small Tafel slope of 29 mV dec^−1^ in 0.5 M H_2_SO_4_, a low overpotential of 28 mV to achieve a current density of 10 mA cm^−2^, excellent mass activity of 1203 mA mg_Pt_^−1^ at the overpotential of 50 mV and a high TOF of 6.1 s^−1^, greatly surpassing the commercial Pt/C catalyst (58 mV dec^−1^, 30 mV, 148 mA mg_Pt_^−1^ and 0.47 s^−1^, respectively). Likewise, in alkaline and neutral electrolytes, the Pt-MXene catalyst also displays better HER activity than the commercial Pt/C. Theoretical calculations indicate that MXene with a high content of N displays a semiconductor-like behavior, while with Pt doping from a single atom to Pt clusters, MXene gradually exhibits a metallic behavior and enhanced electronic conductivity, and the strong interaction between Pt clusters and MXene can effectively optimize the absorption/desorption of the H* intermediate, thus contributing to a reduced energy barrier for a HER process. In addition, the metallic behavior after Pt doping enhances charge carrier transfer kinetics. The work has demonstrated that a high content of N in MXene can be an outstanding support substrate for noble metal catalysts to achieve high-performance HER activity via the modulation of electronic structure, which may also apply to many other metal carbonitrides and MXenes.

## Results and Discussion

**Fig. 1:**
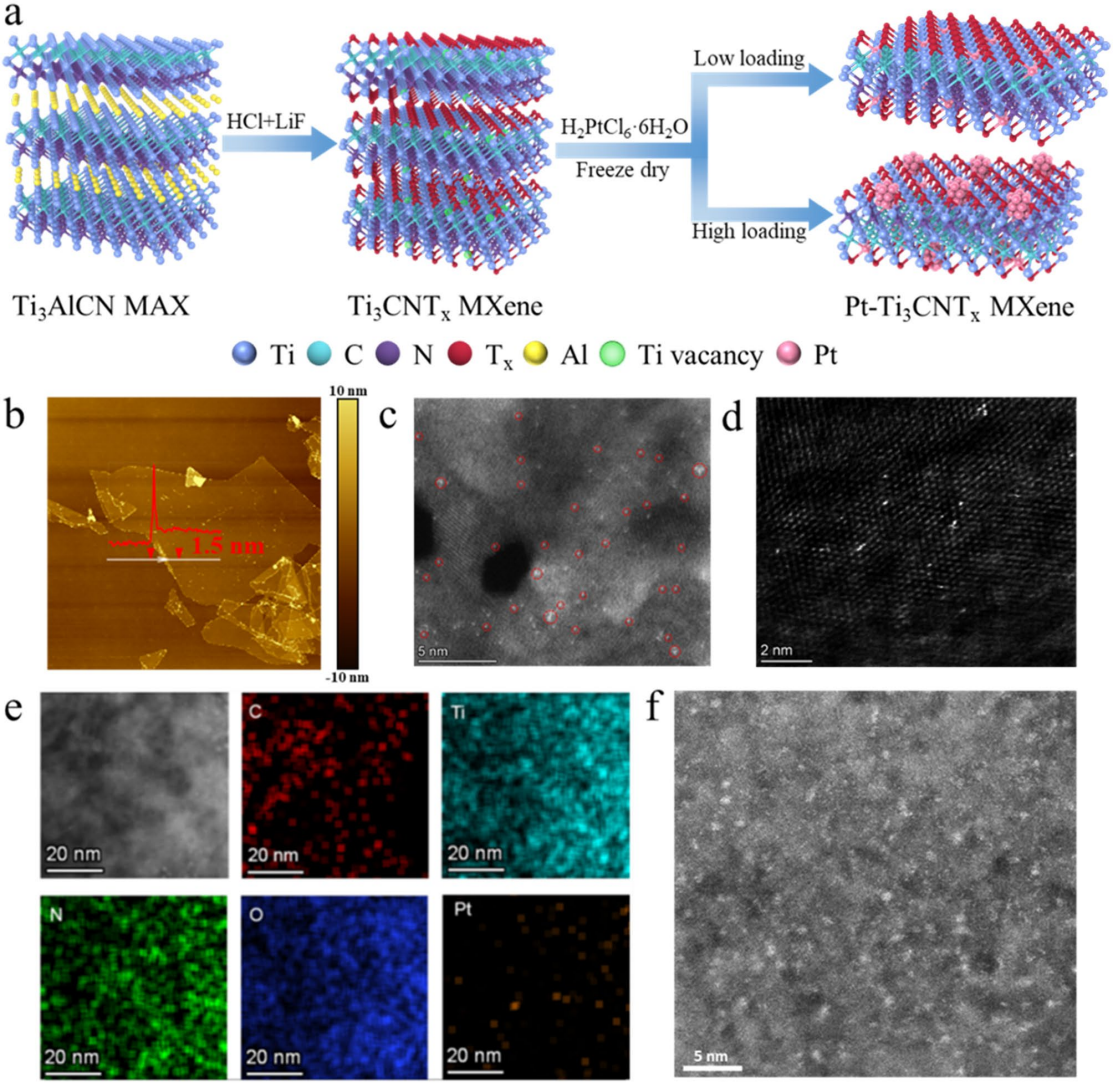
**a** Schematic illustration of the synthesis route of Pt-MXene. **b** AFM image of a typical single-layer MXene nanosheet. **c** HAADF-STEM image of Pt-10 sample with Pt single atoms on MXene nanosheets. **d** The magnified HAADF-STEM image of Pt-10 with Pt single atoms (The bright dots) on MXene nanosheets indicates that Pt single atoms are anchored on Ti vacancies of MXene. **e** High-resolution TEM image of Pt-12.5 sample and the corresponding EDS mapping. **f** HAADF-STEM image of Pt-12.5 sample, showing the co-existence of major Pt clusters and minor Pt single atoms

As illustrated in Fig. 1a, mono-layered or few-layered Ti_3_CNT_x_ MXene nanosheets were prepared through a mild etching strategy in a mixed solution of LiF and HCl. The in-situ formation of HF in the mixed solution acts as the actual etchant, effectively dissolving the Al layer. Meanwhile, the spontaneous intercalation of Li^+^ into the interlayer increases the interlayer spacing of the multilayer MXene to convert into single-layered or few-layered structures during the repeated procedures of washing, centrifuging, and hand-shaking. The as-obtained MXene nanosheets are terminated with abundant surface functional groups, including –O, –OH, and –F. Along with the breaking of Ti–Al bonds, some adjacent Ti atoms on the surface layers are unavoidably removed, leaving reduced Ti vacancies [20, 37]. Besides, numerous low-valence-state Ti (II and III) atoms with highly reducing properties exist in the Ti_3_CNT_x_ MXene. Therefore, when diluted H_2_PtCl_6_·6H_2_O solution is slowly added into the MXene solution, the Pt^4+^ species can undergo through a self-reduction process to settle on the surface of MXene, thus forming Pt single atoms or Pt clusters [23].

Figure S1 displays the XRD patterns of Ti_3_AlCN and Ti_3_CNT_x_. The downshift of the peak from 2θ≈9.5° of Ti_3_AlCN to 7.2° of Ti_3_CNT_x_ and the disappearance of the strongest characteristic peak of Ti_3_AlCN at 2θ≈39° indicates the successful removal of the Al layer and the enlarged interlayer distance [38]. The morphology of exfoliated Ti_3_CNT_x_ ultrathin nanoflakes was characterized by SEM and atomic force microscopy (AFM). The SEM image (Fig. S2a) reveals the well-dispersed large Ti_3_CNT_x_ nanosheets, confirming successful etching via the mild method. The summarized lateral size distribution of the nanoflakes (Fig. S2b) demonstrates a mean lateral size of 2 ± 1 µm. AFM analysis in Fig. 1b presents that the thickness of a typical monolayer Ti_3_CNT_x_ MXene nanosheet is around 1.5 nm, and Fig. S3 further verifies that the synthesized Ti_3_CNT_x_ nanosheets have a uniform and atomically thin thickness of ~ 2 nm according to the marked height profiles. The TEM image in Fig. S4a displays the stacked few-layered Ti_3_CNT_x_ nanosheets after freeze-drying, and the electro-transparent feature indicates the ultrathin thickness. According to the corresponding EDS mapping of the TEM image, it is seen that widely spread Ti, C, and N elements exist over the Ti_3_CNT_x_ nanosheets. Besides, the rich O and F elements represent the rich –F and –O terminations on the surface of Ti_3_CNT_x_. The high-resolution TEM image of Ti_3_CNT_x_ nanosheets in Fig. S4b shows the typical MXene structure with the inset confirming the magnified lattice fringe of Ti_3_CNT_x,_ showing a (100) crystal plane. Furthermore, the XPS survey spectrum of Ti_3_CNT_x_ (Fig. S5) shows the disappearance of Al 2*s* and Al 2*p* peaks, but the presence of O and F peaks, representing the successful exfoliation and rich surface terminations. This is consistent with the EDS mapping results.

SEM images (Fig. S6) reveal the 2D paper-scrap-like structure of Pt-MXene samples after freeze-drying, suggesting that the addition of H_2_PtCl_6_·6H_2_O solution does not have a significant influence on the morphologies of MXene nanosheets. Notably, the nanosheets were stacked together during the freeze-drying, thus resulting in a larger lateral size. To demonstrate the Pt species at the atomic scale, high-angle annular dark-field scanning transmission electron microscopy (HAADF-STEM) images, together with EDS mapping, were taken to study the configuration and dispersion of Pt in all Pt-MXene samples. The TEM images and corresponding EDS elemental mapping (Figs. 1e and S7, S8) reveal the widespread and uniform distribution of Ti, C, N, O, and Pt species over the catalysts. It is evidenced by HAADF imaging that when introducing a low-content Pt precursor, well-dispersed Pt single atoms were observed over the MXene surface (Figs. 1c and S7b, d, f). It is widely accepted that the etching-induced Ti vacancies on the surface of MXene can serve as the anchoring sites to accommodate the Pt atoms [20, 23, 39]. Figure 1d clearly shows that Pt atoms are located in the Ti lattice. With the increase of Pt precursors, Pt clusters gradually emerge and present a homogeneous distribution over the MXene surface (Fig. 1f). A clear transition is observed between Pt-MXene-10 and Pt-MXene-12.5 samples, where the former mainly contains Pt single atoms, while the latter exhibits Pt clusters with an average size of less than 1 nm in diameter (Fig. 1f). As to the specimens with a higher Pt loading, the characteristic (111), (200), and (220) planes of tiny Pt nanocrystallites are also observed through HRTEM images (Fig. S8d). Inductively coupled plasma-mass (ICP) spectrometer and X-ray Fluorescence (XRF) analyses confirm that the Pt loading amount is 2.21, 3.19, 4.2, and 6.25 wt% for Pt-MXene-5, Pt-MXene-10, Pt-MXene-12.5, and Pt-MXene-15, respectively.

**Fig. 2:**
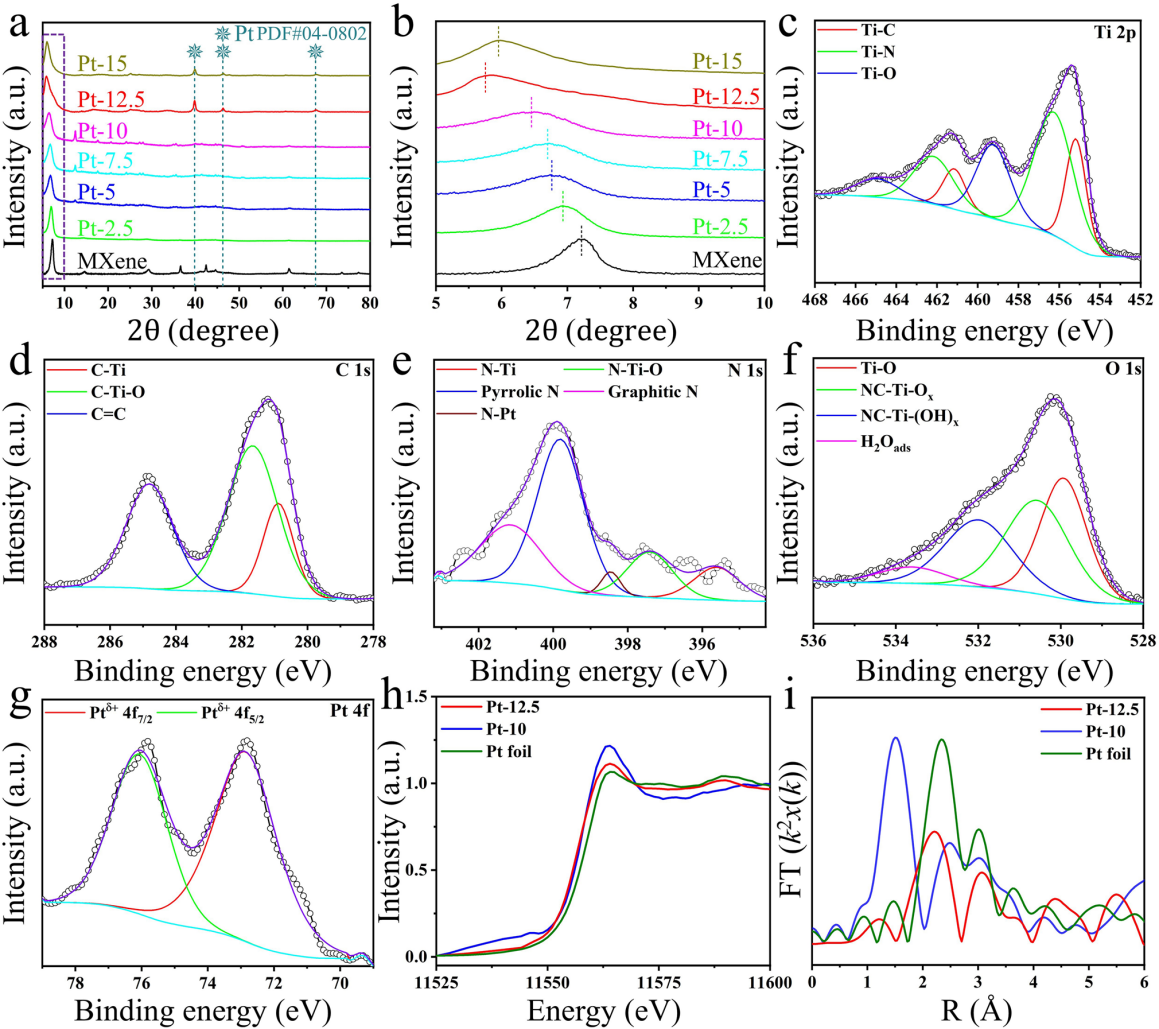
**a** XRD patterns of pure MXene and all Pt-MXene samples with different Pt loadings. **b** Magnified XRD patterns showing the 2θ region from 5° to 10°. **c** High-resolution Ti 2*p* spectra of Pt-12.5 sample. **d** High-resolution C 1*s* spectra of Pt-12.5 sample. **e** High-resolution N 1*s* spectra of Pt-12.5 sample. **f** High-resolution O 1*s* spectra of Pt-12.5 sample. **g** High-resolution Pt 4*f* spectra of Pt-12.5 sample. **h** Normalized XANES spectra at the Pt L_3_-edge of Pt foil, Pt-10, and Pt -12.5 samples. **i** FT-EXAFS spectra derived from EXAFS of Pt L_3_-edge of Pt foil, Pt-10, and Pt-12.5 samples

As shown in Fig. 2a, the XRD patterns of all Pt-MXene samples indicate that till Pt-MXene-10, the characteristic peaks of Pt crystalline cannot be detected. However, with the increasing Pt concentration from Pt-MXene-10 to Pt-MXene-12.5, the characteristic peaks of Pt (2θ≈39.7°, 46.2°, 67.5°) start to emerge, indicating the formation of Pt clusters [40]. Besides, the increase of Pt concentration results in the left shift of the characteristic (002) peak from 7.2° of pure MXene toward lower angles (Fig. 2b), which are 6.93°, 6.76°, 6.7°, 6.45°, 5.75°, and 5.95° for Pt-MXene-2.5, 5, 7.5, 10, 12.5, and 15, respectively. This suggests a strong interaction of Pt with the MXene that results in the further enlargement of interplanar spacing [23]. It is noted that when the Pt concentration increases from Pt-MXene-12.5 to Pt-MXene-15, the (002) diffraction suddenly shifts toward a higher angle, which could be attributed to large amount of Pt clusters formation, which makes the Pt atoms away from lattice [33]. To conclude, the Pt-MXene-12.5 sample shows the largest interplanar spacing, which benefits the penetration of electrolyte ions and the exposure of accessible active sites during the electrocatalytic reaction.

XPS spectra for all Pt-MXene samples were measured to analyze the elemental composition, coordination, and oxidation state of Pt species. In the Ti 2*p* region (Fig. 2c), three Ti 2*p*_3/2_ peaks at 455.2, 456.3, and 459.2 eV for Pt-MXene-12.5 can be assigned to Ti–C, Ti–N, and Ti– O bonds, respectively. Furthermore, it could be found that these peaks in Ti_3_CNT_x_ assigned to Ti–C, Ti–N, and Ti– O bonds (454.9, 456, and 458.7 eV) shifted to higher energy in Pt-MXene-2.5 (455, 456.4, and 458.9 eV), Pt-MXene-5 (455.5, 456.7, and 459.3 eV), Pt-MXene-7.5 (455.2, 456.6, and 458.9 eV) and Pt-MXene-10 (455.4, 456.8, and 459 eV), which validated that the oxidation state of Ti increased due to the addition of Pt atoms. Importantly, peaks at higher eV values (after 460 eV) also contributed to low-valence Ti [35, 41]. The high-resolution C 1*s* spectra (Fig. 2d) exhibits three typical peaks at 280.9, 281.7, and 284.8 eV, representing C–Ti, C–Ti–O, and C=C bonds, respectively [24, 41]. The high-resolution N 1*s* spectra can be deconvoluted into five characteristic peaks at 395.6, 397.4, 398.5, 399.8, and 401.1 eV, which are attributed to N–Ti, N–Ti–O, N–Pt, pyrrolic N, and graphitic N, respectively (Fig. 2e) [24, 28, 42]. To note, the pyrrolic N and graphitic N are only observed in Pt-MXene samples, indicating the formation of Pt–N bonds after the anchoring of Pt species. This confirms the enhanced synergy and stabilization of Pt in N-based MXene. Besides, similar characteristic peaks are also observed in the Ti 2*p*, C 1*s*, and N 1*s* spectra for pure MXene and other Pt-MXene samples (Figs. S9–S14), which implies the structure of MXene is preserved well after the incorporation of Pt. As for the O 1*s* spectra (Fig. 2f), the signals at 529.9, 530.6, 532.0, and 533.6 eV demonstrate the existence of Ti–O, NC–Ti–O_x_, NC–Ti–(OH)_x_ bonds and chemically absorbed H_2_O molecules [41, 43]. Analogous peaks are also observed in the O 1*s* spectra of other Pt-MXene samples (Fig. S14c). As shown in Fig. 2g, the Pt 4*f* spectra of Pt-12.5 sample can be deconvoluted to the corresponding 4*f*_7/2_ and 4*f*_5/2_ peaks at 72.9 and 76.1 eV, respectively. Compared to Pt^0^ and Pt^2+^ with their 4*f*_7/2_/4*f*_5/2_ peaks indexed to 71.2/74.5 and 73.4/76.7 eV [44], it can be seen that the oxidation state of the Pt atoms in Pt-MXene-12.5 sample is between 0 and + 2. This indicates that the Pt^4+^ in its [PtCl_6_]^2–^ precursor was reduced by the Ti and other vacancies, and a strong electronic interaction was established between Pt and MXene substrate [37, 44]. Likewise, the Pt 4*f* spectra in other Pt-MXene samples also display similar peaks corresponding to the partially positive charged Pt atoms (Fig. S15). However, minor 4*f*_7/2_/4*f*_5/2_ peaks for Pt^0^ are only revealed in Pt-MXene-15 sample (Fig. S15e), which is caused by the high-loading Pt content that results in the formation of large Pt particles as identified in high-resolution TEM analysis. X-ray absorption spectroscopy (XAS) was implemented to further investigate the coordination environment of Pt atoms in Pt-MXene samples. Figure 2h shows the X-ray absorption near-edge structure (XANES) of Pt foil, Pt-MXene-10, and Pt-MXene-12.5 samples, respectively. The prepared Pt-MXene-12.5 sample shows a stronger white-line intensity than Pt foil, suggesting that Pt atoms in Pt-MXene-12.5 carry a partial positive charge, consistent with the XPS results [24]. Nevertheless, it is worth mentioning that the Pt-MXene-10 sample exhibits a higher white-line intensity than the Pt-MXene-12.5 sample. This is because Pt single atoms predominate in the Pt-MXene-10 sample, while the Pt cluster is the major species in the Pt-MXene-12.5 sample. Furthermore, this confirms that Pt in the single atom state has a higher valence than the cluster. The literature revealed that low-valence Pt active sites are desirable for HER performance, as they facilitate the enhanced adsorption and desorption of reaction intermediates [45, 46]. Fourier transform extended X-ray absorption fine structure (FT-EXAFS) analysis in Fig. 2i displays that the Pt-MXene-10 sample exhibits a dominant peak at ~ 1.5 Å and a secondary peak at ~ 2.5 Å, which can be indexed to Pt–N/C/O coordination and Pt–Pt bonds, respectively, due to the presence of a small fraction of clusters [47–49]. It is hard to identify the Pt–N, Pt–C, and Pt–O bonds due to the similar back-scattering phases or bond lengths when they exist simultaneously [47–49]. As for the Pt-MXene-12.5 sample, an obvious peak at ~ 2.2 Å from Pt–Pt contribution is observed owing to the existence of massive Pt clusters, and a weak peak at ~ 1.25 Å can be ascribed to Pt–N/C/O scattering since there exist a small quantity of Pt single atoms.

**Fig. 3:**
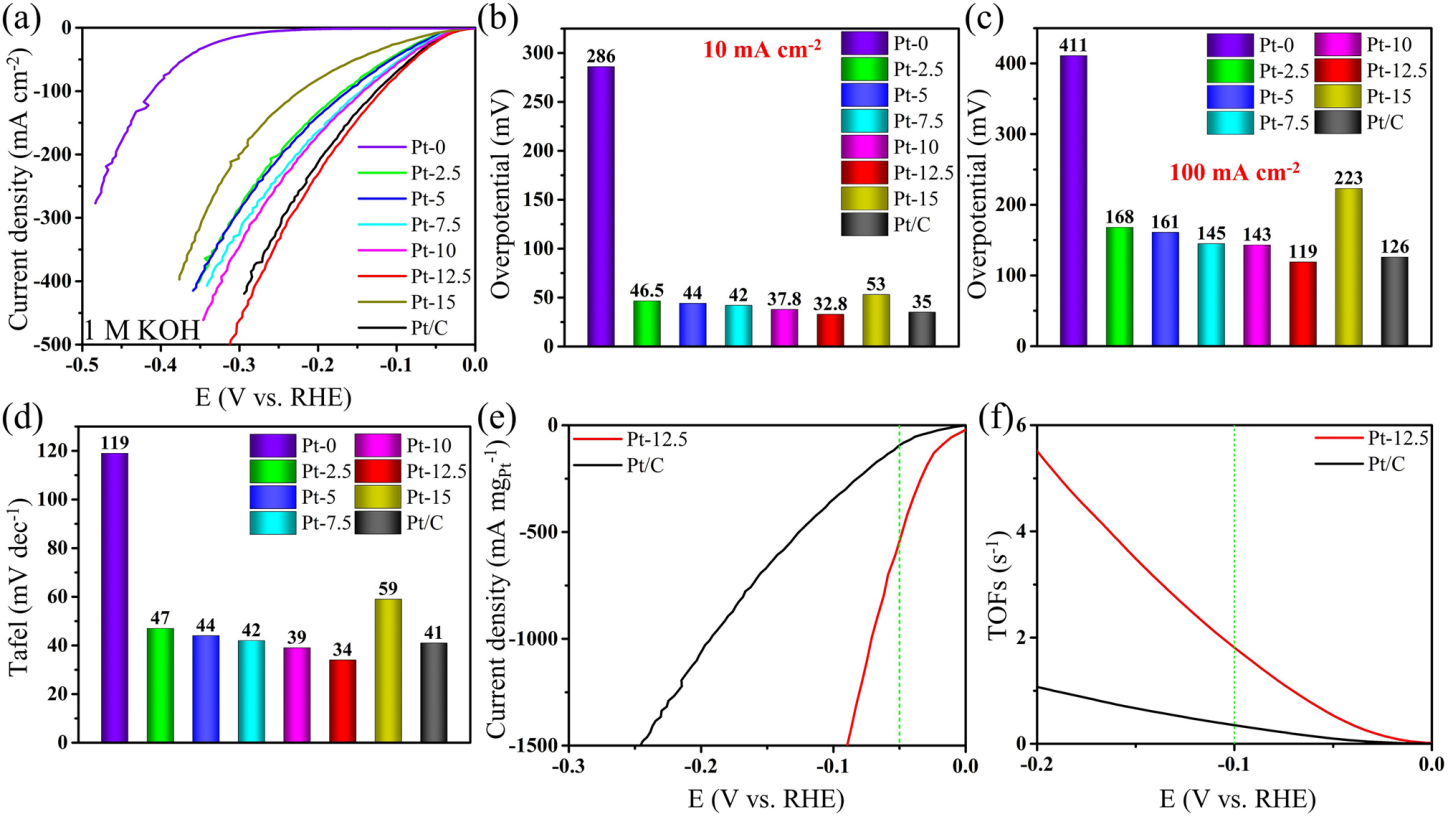
**a** LSV polarization curves in 1 M KOH electrolyte for MXene (denoted as Pt-0), Pt/C, and all Pt-MXene samples. **b** The corresponding overpotential at 10 mA cm^−2^. **c** The corresponding overpotential at 100 mA cm^−2^. **d** Tafel slope plots for MXene, Pt/C and all Pt-MXene samples. **e** Mass activity of Pt-MXene-12.5 and Pt/C. **f** TOF plots of Pt-MXene-12.5 and Pt/C

The HER electrocatalytic performance was first measured via a standard three-electrode device with 1.0 M KOH as the electrolyte. Among all Pt-MXene samples (Fig. 3a, b), Pt-MXene-12.5 exhibits the lowest overpotential of 32.8 mV to realize 10 mA cm^–2^ current density for HER, which greatly surpasses the 286 mV of pure MXene and slightly outperforms that of the state-of-the-art commercial Pt/C catalyst (35 mV). Noticeably, with the increase in Pt loading, the overpotentials of Pt-MXene samples first exhibit a decreasing trend and then increase (46.5, 44, 42, 37.8, 32.8, and 53 mV for Pt-MXene-2.5, 5, 7.5, 10, 12.5, and 15, respectively), which indicates the Pt-MXene-12.5 sample has the optimal Pt loading for HER. The first decrease in the overpotential with the increase of Pt content could be ascribed to the enhanced active site number and synergy between Pt and MXene. However, after Pt-MXene-12.5, the increase in overpotential is due to the agglomeration of Pt sites and restacking of MXene nanosheets that result in the low exposure of active sites. Moreover, at a higher current density of 100 mA cm Pt-MXene^–2^, the overpotential of Pt-MXene-12.5 was 119 mV, which is still lower than the commercial Pt/C catalyst of 126 mV (Fig. 3c). Figure 3a also shows that Pt-MXene-12.5 only requires an overpotential of 312 mV to achieve a high current density of 500 mA cm^−2^, representing its potential for practical applications. The Tafel slope, which reflects the logarithmic relationship between the reaction rate and overpotential, serves as an indicator of catalytic performance. As shown in Figs. 3d and S16a, b, the optimal Tafel slope belongs to the Pt-MXene-12.5 with only 34 mV dec^−1^. In comparison, the commercial Pt/C catalyst and pure MXene deliver lower Tafel slopes of 41 and 119 mV dec^−1^, respectively, indicating that Pt-MXene-12.5 achieves a faster HER kinetics than the Pt/C catalyst and pure MXene due to the strong interaction between MXene and Pt clusters. Likewise, the Tafel slopes of all Pt-MXene samples first decrease and then increase with Pt addition (47, 44, 42, 39, 34, and 59 mV for Pt-MXene-2.5, 5, 7.5, 10, 12.5, and 15, respectively). This improved HER kinetics for Pt-MXene-12.5 could be ascribed to the cluster formation that facilitates hydrogen adsorption and diffusion. Adjacent hydrogen adsorption sites in clusters increase the Heyrovsky and Tafel steps of the HER mechanism [50, 51]. To more objectively compare the catalytic activity of Pt-MXene-12.5 and Pt/C, their current densities are normalized to the loading amount of Pt to obtain an intuitive comparison of their mass activities. As displayed in Fig. 3e, Pt-MXene-12.5 exhibits a more favorable HER performance in mass activity than the commercial Pt/C catalyst. Specifically, at the overpotential of 50 mV, Pt-MXene-12.5 reaches a mass activity of 545 mA mg_Pt_^−1^, which is six times larger than that of Pt/C (89 mA mg_Pt_^−1^). This confirms the improvement in the intrinsic catalytic activity of MXene after Pt addition. Electrochemical impedance spectroscopy (EIS) was conducted to investigate the charge transfer kinetics for HER of Pt-MXene, MXene, and a commercial Pt/C. The evident semicircular shape of the Nyquist plot proves the electrode process as a control step (Fig. S16c). As displayed in Fig. S16d, e, Pt-MXene-12.5 possesses the lowest charge transfer resistance (*R*_ct_) (1.78 Ω), which is much lower than that of the commercial Pt/C (2.91 Ω) and MXene (101 Ω), indicating the better electronic conductivity and ultrafast electron transport behavior. Furthermore, it is found that all Pt-MXene samples possess much lower R_ct_ compared to individual MXene, demonstrating that the incorporation of Pt in MXene greatly enhances the electronic conductivity and lower resistance to fast-electron transfer. To compare with the MXene without nitrogen content, we prepared Ti_3_C_2_T_x_ nanosheets doped with different Pt contents. The optimal sample (12.5 at% Pt) LSV curve is shown in Fig. S16f, which shows much lower performance.

Double-layer capacitance (*C*_dl_) was also measured to assess the electrochemical behavior of Pt-MXene samples and MXene counterparts. Figures S17 and S18 display the cyclic voltammogram curves of different Pt-MXene samples under various scan rates from 10 to 20, 40, 60, 80, and 100 mV s^–1^. The calculated *C*_dl_ (Fig. S18d) is 43.51, 76.49, 78.04, 84.39, 87.33, 92.15, and 63.65 mF cm^–2^ for MXene, Pt-MXene-2.5, 5, 7.5, 10, 12.5, and 15, respectively. Therefore, Pt-MXene-12.5 owns the largest electrochemically active surface area (ECSA), which is ~ 2.12 times higher than the value of MXene. This can be attributed to the introduction of Pt species, which greatly improves the number of active sites exposed on the surface of MXene and simultaneously enlarges the interplanar spacing that facilitates the contact between electrolyte and catalyst. Besides, the turnover frequency (TOF) was calculated to further evaluate the intrinsic hydrogen-production activity of the electrocatalysts. Figure 3f shows that Pt-MXene-12.5 achieves an outstanding TOF of 1.83 s^−1^ at an overpotential of 100 mV, which significantly surpasses that of the commercial Pt/C (0.33 s^−1^). This verifies the improved synergy between Pt and MXene that contributes to enhancing intrinsic and extrinsic catalytic activity for high HER performance. The catalytic stability is also a crucial factor in evaluating the feasibility of the electrocatalyst in practical usage. At a constant current density of 10 mA cm^−2^, Pt-MXene-12.5 shows a little degradation after 12 h, almost comparable to the commercial Pt/C catalyst (Fig. S19a). This superior stability is attributed to the strong synergy and interaction of Pt moieties with high N-content MXenes. Additionally, we also performed the stability at the current density of 200 mA cm^−2^ up to 23 h, as shown in the inset of Fig. S19a. No obvious degradation is observed, indicating outstanding stability. This clarifies the excellent long-term stability of Pt-MXene-12.5. Thanks to the abundant Pt clusters, Pt-MXene-12.5 almost becomes one of the most outstanding HER catalysts compared to most recently reported MXene-based catalysts with 1 M KOH as the electrolyte (Fig. S19b and Table S1).

**Fig. 4:**
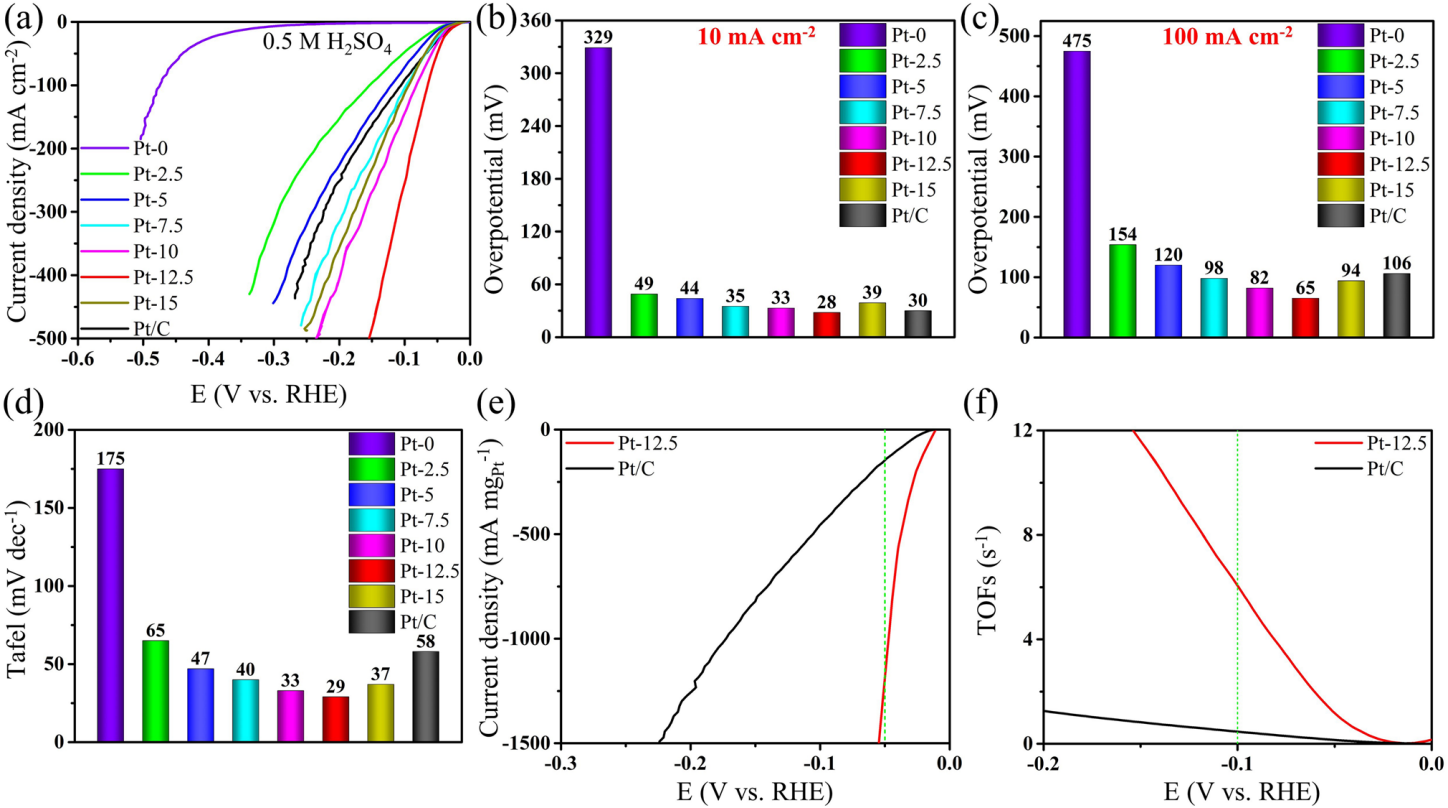
**a** LSV polarization curves and (b, c) the corresponding overpotential @ 10 mA cm^−2^ **b** and @ 100 mA cm^−2^ **c** Plots in 0.5 M H_2_SO_4_ electrolyte for MXene, Pt/C and all Pt-MXene samples. **d** Tafel slope plots of MXene, Pt/C and all Pt-MXene samples in 0.5 M H_2_SO_4_ solution. **e** Mass activity of Pt-MXene-12.5 and Pt/C. **f** TOF plots of Pt-MXene-12.5 and Pt/C

To further investigate the pH universality of Pt-MXene samples, 0.5 M H_2_SO_4_ was also used as the electrolyte to measure the HER activity. In Fig. 4a, b, Pt-MXene-12.5 only requires a small overpotential of 28 mV to realize a current density of 10 mA cm^–2^, which overmatches the value of commercial Pt/C (30 mV) and dramatically surpasses that of MXene (329 mV). In addition, with the increase of Pt concentration, the overpotential shows a gradual decrease and then increases again, confirming the optimal loading of Pt-MXene-12.5. Notably, at a higher current density of 100 mA cm^–2^, the overpotential of the Pt-MXene-12.5 sample is only 65 mV, much lower than that of the commercial Pt/C (106 mV) and pure MXene without nitrogen integrated with Pt catalysts (Fig. S20f), illustrating the superiority of Pt-MXene-12.5 (Fig. 4c). Even to achieve a current density of 500 mA cm^–2^, Pt-MXene-12.5 only requires an overpotential of 154 mV, which greatly exceeds the commercial Pt/C catalyst and confirms its promising applicability in large-scale electrolyzers (Fig. 4a). The Tafel slope of Pt-MXene-12.5, commercial Pt/C, and MXene are 29, 58, and 175 mV dec^–1^ (Figs. 4d and S20a, b), validating the ultrafast HER kinetics of Pt-MXene-12.5 due to the optimized interplay between Pt moieties and MXene support. Meanwhile, the Tafel slopes of all Pt-MXene samples show a similar trend with the overpotential, which first goes down and then ascends in accompany with the increase of Pt loading (65, 47, 40, 33, 29, and 37 mV dec^–1^ for Pt-MXene-2.5, 5, 7.5, 10, 12.5, and 15, respectively). Besides, the mass activity of Pt-MXene-12.5 reaches up to 1203 mA mg_Pt_^−1^ at an overpotential of 50 mV, which is 8 times higher than that of the commercial Pt/C catalyst (148 mA mg_Pt_^−1^), as shown in Fig. 4e. This result further confirms the superiority of Pt-MXene-12.5 in realizing high HER performance compared to the Pt/C catalyst in both alkaline and acidic electrolytes. Figure 4f shows that the TOF of Pt-MXene-12.5 is 6.1 s^−1^ at an overpotential of 100 mV, nearly 13 times higher than the values of the commercial Pt/C catalyst (0.47 s^−1^). This demonstrates the full utilization of Pt clusters in Pt-MXene-12.5, thereby leading to remarkable intrinsic activity.

In accordance with the electrochemical impedance spectroscopy (EIS) results (Fig. S20c), the fitted *R*_ct_ in Fig. S20d, e demonstrates that Pt-MXene-12.5 exhibits the optimal charge transfer kinetics with a small value of 1.14 Ω, outperforming the commercial Pt/C (4.36 Ω) and MXene (500 Ω). Moreover, other Pt-MXene samples also show much smaller R_ct_ than MXene, which reveals that the introduction of Pt species can serve as the active centers to accelerate the electron transfer during HER. Besides, after the stability test at 200 mA cm^–2^ for ~ 22.3 h, Pt-MXene-12.5 still exhibits very good stability without observable degradation (Fig. S21a). The initial variation may result from the reconstruction of catalysts under such a high current [52]. Pt-MXene-12.5 has shown extremely low overpotential and Tafel slope, which differ from other MXene samples with either low overpotential but high Tafel slope or vice versa (Fig. S21b and Table S2).

The HER activity of Pt-MXene samples was also studied in a neutral electrolyte (0.5 M K_2_SO_4_). To achieve a current density of 10 mA cm^−2^, Pt-MXene-12.5 requires the lowest overpotential of 366.4 mV among all Pt-MXene samples, which is better than the value of commercial Pt/C (373.9 mV), while MXene demands 590.3 mV (Fig. S22a, b). The Tafel slope of Pt-MXene-12.5 is the lowest at 83 mV dec^−1^ among all Pt-MXene samples, which is also superior to the commercial Pt/C catalyst (86 mV dec^−1^) (Fig. S22c–e). Moreover, all Pt-MXene samples showed more advantageous Tafel slopes than MXene (148 mV dec^−1^) (Fig. S22c–e). This indicates that the association of Pt and MXene can accelerate the reaction kinetics, thus boosting the HER catalytic performance. As displayed in Fig. S23a, Pt-MXene-12.5 delivers a much more favorable mass activity of 480 mA mg_Pt_^−1^ at an overpotential of 400 mV compared to that of the commercial Pt/C catalyst (89 mA mg_Pt_^−1^). Moreover, the TOF values of Pt-MXene-12.5 is 0.48 s^−1^, which is over 5 times higher than that of the commercial Pt/C (0.09 s^−1^) (Fig. S23b). According to the experimental EIS and the corresponding fitting results (Fig. S23c, d), Pt-MXene-12.5 exhibits the lowest *R*_ct_ of 79.7 Ω, which is lower than that of the commercial Pt/C catalyst (86.6 Ω) and MXene (234.3 Ω). This reveals that introducing Pt into MXene can facilitate the Faradaic reaction and the HER catalytic kinetics. Furthermore, the stability test indicates that at a constant current density of 10 mA cm^−2^, Pt-MXene-12.5 shows a nearly zero potential fluctuations after 16-h galvanostatic measurement, which is on a par with the performance of the commercial Pt/C catalyst (Fig. S24).

**Fig. 5:**
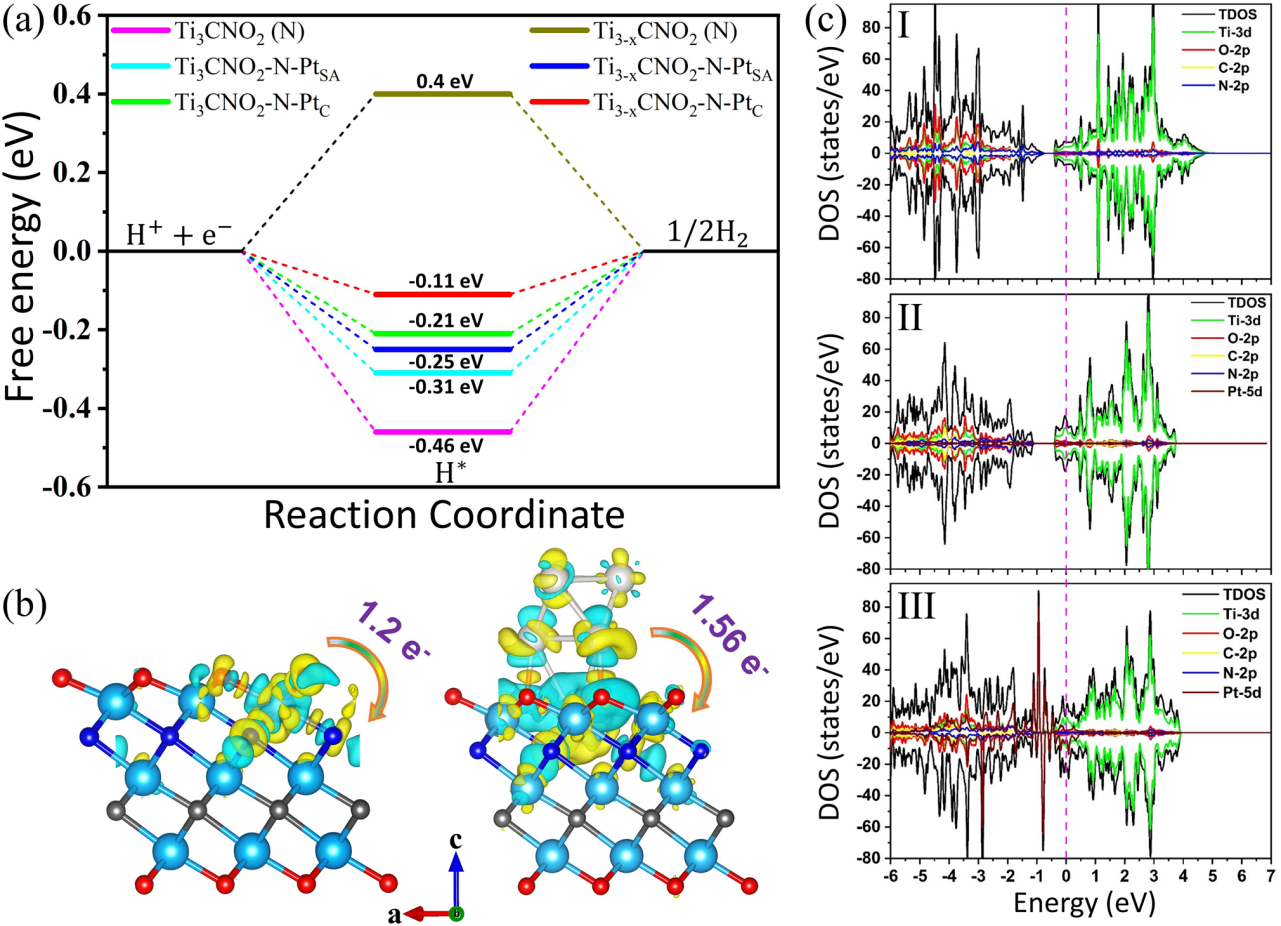
**a** Calculated free energies for hydrogen evolution on Ti_3_CNO_2_ (N) MXene, Ti_3_CNO_2_-N-Pt_SA_, Ti_3_CNO_2_-N-Pt_C_, Ti_3-x_CNO_2_ (N) MXene, Ti_3-x_CNO_2_-N-Pt_SA_, and Ti_3-x_CNO_2_-N-Pt_C_. **b** Charge density difference plots of Ti_3-x_CNO_2_-N-Pt_SA_ (left) and Ti_3-x_CNO_2_-N-Pt_C_ (right). The blue, gray, dark blue, red and white atoms represent Ti, C, N, O, and Pt, respectively. **c** DOS plots of Ti_3-x_CNO_2_ (N) MXene (I), Ti_3-x_CNO_2_-N-Pt_SA_ (II), and Ti_3-x_CNO_2_-N-Pt_C_ (III)

Density functional theory (DFT) calculations were conducted to unveil the mask of the underlying interaction between Pt species and Ti_3_CNT_x_ MXene. According to the above experimental results, MXene-supported Pt clusters with the optimized Pt loading show a better HER performance than MXene-supported Pt single atoms. Therefore, twelve different models were constructed to investigate the Pt catalysts on the HER performance and to understand the mechanism of better performance of Pt clusters rather than Pt single atoms, as well as to clarify the different Pt bonding states on the catalytic activity for hydrogen production (Table S3). Due to the rich N in this MXene, we first consider the effect of the formation of Pt–N bonding on the HER performance. Pt–N bonding has been identified in the previous XPS and XAS results. Consequently, the constructed six models are pristine Ti_3_CNO_2_ (N) MXene, Ti_3_CNO_2_-N-Pt_SA_, Ti_3_CNO_2_-N-Pt_C_, Ti_3-x_CNO_2_ (N) MXene, Ti_3-x_CNO_2_-N-Pt_SA_, and Ti_3-x_CNO_2_-N-Pt_C_. It should be noted that for these models without Ti vacancies, the Pt species are immobilized by the surface O atoms. These structural models are displayed in Fig. S25. Generally, the Gibbs free energy ($\Delta {G}_{{H}^{*}}$) for hydrogen adsorption is regarded as a key descriptor to evaluate the HER activity of the catalyst [19, 32]. Moreover, the best HER catalyst should have the smallest value of $\Delta {G}_{{H}^{*}}$, which is ideally close to zero. As shown in Fig. 5a, Ti_3_CNO_2_ (N) MXene, Ti_3_CNO_2_-N-Pt_SA_, Ti_3_CNO_2_-N-Pt_C_, Ti_3-x_CNO_2_ (N) MXene, Ti_3-x_CNO_2_-N-Pt_SA_ and Ti_3-x_CNO_2_-N-Pt_C_ has a value of $\Delta {G}_{{H}^{*}}$ –0.46, –0.31, –0.21, 0.40, –0.25, and –0.11 eV, respectively (Table S3). Ti_3-x_CNO_2_-N-Pt_C_ has the smallest value of –0.11 eV, which highlights that Ti_3-x_CNO_2_-N-Pt_C_ possesses the optimal hydrogen absorption–desorption behavior and the lowest energy barrier to form H_2_, thus contributing to the superior HER catalytic activity [28]. According to the charge density difference calculations in Figs. 5b and S26a, 1.56 electrons are transferred from Pt cluster to MXene substrate in Ti_3-x_CNO_2_-N-Pt_C_, while only 0.1, 0.3, and 1.2 electrons are donated to MXene substrates in Ti_3_CNO_2_-N-Pt_SA_, Ti_3_CNO_2_-N-Pt_C_, Ti_3-x_CNO_2_-N-Pt_SA_, respectively. This indicates stronger electronic interaction between the Pt cluster and MXene in Ti_3-x_CNO_2_-N-Pt_C_ compared to other models, which is beneficial to weaken the Pt–H bonding and resulting in favorable hydrogen absorption/desorption [21, 23]. This explains the optimal Gibbs free energies ($\Delta {G}_{{H}^{*}}$) of Ti_3-x_CNO_2_-N-Pt_C_. In addition, from the densities of states (DOS) of the catalysts in Figs. 5c and S26b, it can be concluded that introducing Pt clusters into MXene support can endow the catalyst with a distinguished metallic characteristic and enhanced electronic conductivity, which are not observed in individual MXene and MXene-supported Pt_SA_ [28]. Furthermore, Ti_3-x_CNO_2_-N-Pt_C_ exhibits a higher total density of states (TDOS) near the Fermi level than those of Ti_3_CNO_2_ (N) MXene, Ti_3_CNO_2_-N-Pt_SA_, Ti_3_CNO_2_-N-Pt_C_, Ti_3-x_CNO_2_ (N) MXene and Ti_3-x_CNO_2_-N-Pt_SA_, illustrating that Ti_3-x_CNO_2_-N-Pt_C_ possesses an enhanced carrier density and capability to supply electrons, consistent with experimental results [19, 28, 32]. The fast transfer of electrons favors improved electrocatalytic HER. Based on the projected densities of states (PDOS) of Ti_3-x_CNO_2_ (N) MXene, Ti_3-x_CNO_2_-N-Pt_SA_, and Ti_3-x_CNO_2_-N-Pt_C_, the DOS of Ti_3-x_CNO_2_ (N) MXene and Ti_3-x_CNO_2_-N-Pt_SA_ near the Fermi level primarily originate from the d orbitals of Ti. However, a tiny contribution from d-orbitals of Pt is observed in Ti_3-x_CNO_2_-N-Pt_SA_, while that of Ti_3-x_CNO_2_-N-Pt_C_ is donated by the d-orbitals of Ti and Pt. This signifies that Pt clusters significantly enhance the capability to donate d-electrons near the Fermi level, thus contributing to an improved HER catalytic activity.

Given that Pt-C bonds are also formed during the preparation of catalysts, we construct six other models as counterparts to illustrate the influence of C atoms on HER performance when they are bonded with the Pt species (Fig. S27). Besides, the Pt species are anchored by the surface O atoms for these models without Ti vacancies. As shown in Fig. S28a, Ti_3-x_CNO_2_-C-Pt_C_ exhibits the best Gibbs free energies ($\Delta {G}_{{H}^{*}}$) of –0.15 eV, which indicates that the existence of Ti vacancies and Pt clusters are beneficial to optimize the hydrogen absorption–desorption behavior and lower the energy barrier to form H_2_, thus contributing to the superior HER catalytic activity. The charge density difference calculations in Fig. S28b–e confirm that more electrons are transferred between Pt clusters and MXene support in Ti_3-x_CNO_2_-C-Pt_C_ (1.51 electrons), compared with Ti_3-x_CNO_2_-C-Pt_SA_ (0.83 electrons), Ti_3_CNO_2_-C-Pt_C_ (0.54 electrons) and Ti_3_CNO_2_-C-Pt_SA_ (0.13 electrons). This means a stronger electronic interaction exists between the Pt clusters and MXene in Ti_3-x_CNO_2_-C-Pt_C_ compared to other models, thus facilitating HER activity. According to the DOS of the catalysts in Fig. S29, it can be concluded that although all six catalysts exhibit superior metallic behavior, the introduction of Pt clusters still significantly elevates the total densities of states (TDOS) near the Fermi level, which highlights an improved carrier density and capability to supply electrons, thus boosting the HER performance. Especially for Ti_3-x_CNO_2_-C-Pt_C_, its DOS near the Fermi level is primarily contributed by the d orbitals of Ti and Pt. In contrast, less electron density transfers from the d orbitals of Pt are observed in the rest of the models, which suggests that Pt clusters enhance the catalyst’s capability to donate electronic density near the Fermi level, thus reinforcing the HER catalytic activity. To conclude, regardless of whether Pt is bonded with N or C, the association of Pt clusters and Ti_3-x_CNO_2_ MXene can always strengthen the electronic conductivity and optimize the $\Delta {G}_{{H}^{*}}$ to accomplish the improvement of HER catalytic capability, well consistent with our experiment results. However, it is worth mentioning that the existence of C atoms can ensure better electronic conductivity, while the N atoms can contribute to more favorable electronic interactions with the anchored Pt species, as Ti_3-x_CNO_2_-N-Pt_C_ delivers a more favorable $\Delta {G}_{{H}^{*}}$ of –0.11 eV than that of Ti_3-x_CNO_2_-C-Pt_C_ (–0.15 eV). In addition, N atoms may enhance the electronic structure of the MXene surface, thus improving the HER. Therefore, the abundant N atoms in MXene play a crucial role in enhancing HER catalytic activity.

## Conclusion

In summary, ultrathin Ti_3_CNT_x_ MXene nanosheets have been successfully synthesized using a mild etching strategy and employed as support materials for anchoring Pt single atoms and clusters via a facile self-reduction reaction by varying the contents of Pt precursors. The optimized catalyst, with a low Pt loading amount of 4.2 wt% (Pt-MXene-12.5), exhibits exceptional HER catalytic performance across all pH values, outperforming the commercial Pt/C catalyst. It achieves Tafel slopes of 34, 29, and 83 mV dec^–1^ and low overpotentials of 32.8, 28, and 366.4 mV to reach a current density of 10 mA cm^–2^ in 1 M KOH, 0.5 M H_2_SO_4_, and 0.5 M K_2_SO_4_, respectively. Additionally, it demonstrates an impressive mass activity of 1203 mA mg_Pt_^−1^ at an overpotential of 50 mV and high TOF of 6.1 s^−1^ at an overpotential of 100 mV in acidic electrolyte, exceeding those of the commercial Pt/C catalyst by over 8 times and nearly 13 times, respectively. DFT calculations reveal that the synergy between Pt, C, and N atoms in MXene creates a strong electronic interaction between the anchored Pt species and the MXene support. Moreover, the incorporation of Pt clusters can further enhance the metallic behavior of the MXene and simultaneously accumulate more electrons near the Fermi level, which not only brings an improved electronic structure modulation but also optimizes hydrogen absorption/desorption behaviors, thereby lowering the Gibbs free energy toward a fast HER kinetics. This work highlights the potential of the unexplored MXene family, including metal carbonitrides and other MXenes rich in non-metallic elements, as promising support materials for single-atom or cluster catalysts, enabling high-performance electrocatalytic reactions.

## Supplementary Information

Below is the link to the electronic supplementary material.

Supplementary file1 (DOCX 8651 KB)

## References

[1] Z. Lei, J.M. Lee, G. Singh, C.I. Sathish, X. Chu et al., Recent advances of layered-transition metal oxides for energy-related applications. Energy Storage Mater. **36**, 514–550 (2021). 10.1016/j.ensm.2021.01.004

[2] Z. Lei, C. Sathish, Y. Liu, A. Karokoti, J. Wang et al., Single metal atoms catalysts: Promising candidates for next generation energy storage and conversion devices. EcoMat **4**, e12186 (2022). 10.1002/eom2.12186

[3] G. Yasin, S. Ali, S. Ibraheem, A. Kumar, M. Tabish et al., Simultaneously engineering the synergistic-effects and coordination-environment of dual-single-atomic iron/cobalt-sites as a bifunctional oxygen electrocatalyst for rechargeable zinc-air batteries. ACS Catal. **13**, 2313–2325 (2023). 10.1021/acscatal.2c05654

[4] S. Ali, S. Ali, P.M. Ismail, H. Shen, A. Zada et al., Synthesis and bader analyzed cobalt-phthalocyanine modified solar UV-blind β-Ga_2_O_3_ quadrilateral nanorods photocatalysts for wide-visible-light driven H_2_ evolution. Appl. Catal. B Environ. Energy **307**, 121149 (2022). 10.1016/j.apcatb.2022.121149

[5] M. Li, P. Selvarajan, S. Wang, T. Wan, S. Xi et al., Thermostable 1T-MoS_2_ nanosheets achieved by spontaneous intercalation of Cu single atoms at room temperature and their enhanced HER performance. Small Struct. **4**, 2300010 (2023). 10.1002/sstr.202300010

[6] X. Chu, C.I. Sathish, J.-H. Yang, X. Guan, X. Zhang et al., Strategies for improving the photocatalytic hydrogen evolution reaction of carbon nitride-based catalysts. Small **19**, e2302875 (2023). 10.1002/smll.202302875 37309270 10.1002/smll.202302875

[7] X. Guan, M. Fawaz, R. Sarkar, C.-H. Lin, Z. Li et al., S-doped C_3_N_5_ derived from thiadiazole for efficient photocatalytic hydrogen evolution. J. Mater. Chem. A **11**, 12837–12845 (2023). 10.1039/d3ta00318c

[8] C. Li, Z. Chen, H. Yi, Y. Cao, L. Du et al., Polyvinylpyrrolidone-coordinated single-site platinum catalyst exhibits high activity for hydrogen evolution reaction. Angew. Chem. Int. Ed. **59**, 15902–15907 (2020). 10.1002/anie.202005282 10.1002/anie.202005282 PMC7539980 32436325

[9] G. Singh, K. Ramadass, V.D.B.C. DasiReddy, X. Yuan, Y. Sik Ok et al., Material-based generation, storage, and utilisation of hydrogen. Prog. Mater. Sci. **135**, 101104 (2023). 10.1016/j.pmatsci.2023.101104

[10] H. Yin, S. Zhao, K. Zhao, A. Muqsit, H. Tang et al., Ultrathin platinum nanowires grown on single-layered nickel hydroxide with high hydrogen evolution activity. Nat. Commun. **6**, 6430 (2015). 10.1038/ncomms7430 25728293 10.1038/ncomms7430

[11] X. Li, Z. Huang, C.E. Shuck, G. Liang, Y. Gogotsi et al., MXene chemistry, electrochemistry and energy storage applications. Nat. Rev. Chem. **6**, 389–404 (2022). 10.1038/s41570-022-00384-8 37117426 10.1038/s41570-022-00384-8

[12] M. Naguib, M. Kurtoglu, V. Presser, J. Lu, J. Niu et al., Two-dimensional nanocrystals produced by exfoliation of Ti_3_AlC_2_. Adv. Mater. **23**, 4248–4253 (2011). 10.1002/adma.201102306 21861270 10.1002/adma.201102306

[13] H. Yin, Y. Dou, S. Chen, Z. Zhu, P. Liu et al., 2D electrocatalysts for converting earth-abundant simple molecules into value-added commodity chemicals: recent progress and perspectives. Adv. Mater. **32**, e1904870 (2020). 10.1002/adma.201904870 31573704 10.1002/adma.201904870

[14] A. VahidMohammadi, J. Rosen, Y. Gogotsi, The world of two-dimensional carbides and nitrides (MXenes). Science **372**, eabf1581 (2021). 10.1126/science.abf1581 34112665 10.1126/science.abf1581

[15] S. Ali, Z. Xie, H. Xu, Stability and catalytic performance of single-atom supported on Ti_2_CO_2_ for low-temperature CO oxidation: a first-principles study. ChemPhysChem **22**, 2352–2361 (2021). 10.1002/cphc.202100436 34390308 10.1002/cphc.202100436

[16] Y. Zou, S.A. Kazemi, G. Shi, J. Liu, Y. Yang et al., Ruthenium single-atom modulated Ti_3_C_2_T_*x*_ MXene for efficient alkaline electrocatalytic hydrogen production. EcoMat **5**, e12274 (2023). 10.1002/eom2.12274

[17] S.Y. Pang, Y.T. Wong, S. Yuan, Y. Liu, Y. Liu et al., Universal strategy for HF-free facile and rapid synthesis of two-dimensional MXenes as multifunctional energy materials. J. Am. Chem. Soc. **141**, 9610–9616 (2019). 10.1021/jacs.9b02578 31117483 10.1021/jacs.9b02578

[18] M. Li, J. Lu, K. Luo, Y. Li, K. Chang et al., Element replacement approach by reaction with lewis acidic molten salts to synthesize nanolaminated MAX phases and MXenes. J. Am. Chem. Soc. **141**, 4730–4737 (2019). 10.1021/jacs.9b00574 30821963 10.1021/jacs.9b00574

[19] X. Peng, S. Zhao, Y. Mi, L. Han, X. Liu et al., Trifunctional single-atomic Ru sites enable efficient overall water splitting and oxygen reduction in acidic media. Small **16**, e2002888 (2020). 10.1002/smll.202002888 32662944 10.1002/smll.202002888

[20] S. Zhou, Y. Zhao, R. Shi, Y. Wang, A. Ashok et al., Vacancy-rich MXene-immobilized Ni single atoms as a high-performance electrocatalyst for the hydrazine oxidation reaction. Adv. Mater. **34**, e2204388 (2022). 10.1002/adma.202204388 35839429 10.1002/adma.202204388

[21] W. Peng, M. Luo, X. Xu, K. Jiang, M. Peng et al., Spontaneous atomic ruthenium doping in Mo_2_CT_X_ MXene defects enhances electrocatalytic activity for the nitrogen reduction reaction. Adv. Energy Mater. **10**, 2001364 (2020). 10.1002/aenm.202001364

[22] J. Zhang, Y. Zhao, X. Guo, C. Chen, C.-L. Dong et al., Single platinum atoms immobilized on an MXene as an efficient catalyst for the hydrogen evolution reaction. Nat. Catal. **1**, 985–992 (2018). 10.1038/s41929-018-0195-1

[23] Y. Wu, W.-Q. Wei, R. Yu, L. Xia, X. Hong et al., Anchoring sub-nanometer Pt clusters on crumpled paper-like MXene enables high hydrogen evolution mass activity. Adv. Funct. Mater. **32**, 2110910 (2022). 10.1002/adfm.202110910

[24] W. Peng, J. Han, Y.-R. Lu, M. Luo, T.-S. Chan et al., A general strategy for engineering single-metal sites on 3D porous N, P co-doped Ti_3_C_2_T_X_ MXene. ACS Nano **16**, 4116–4125 (2022). 10.1021/acsnano.1c09841 35187929 10.1021/acsnano.1c09841

[25] X. Wang, J. Ding, W. Song, X. Yang, T. Zhang et al., Cation vacancy clusters in Ti_3_C_2_T_*x*_ MXene induce ultra-strong interaction with noble metal clusters for efficient electrocatalytic hydrogen evolution. Adv. Energy Mater. **13**, 2300148 (2023). 10.1002/aenm.202300148

[26] R. Xing, T. Zhou, Y. Zhou, R. Ma, Q. Liu et al., Creation of triple hierarchical micro-meso-macroporous N-doped carbon shells with hollow cores toward the electrocatalytic oxygen reduction reaction. Nano-Micro Lett. **10**, 3 (2018). 10.1007/s40820-017-0157-1 10.1007/s40820-017-0157-1 PMC6199056 30393652

[27] G. Qian, J. Chen, T. Yu, L. Luo, S. Yin, N-doped graphene-decorated NiCo alloy coupled with mesoporous NiCoMoO nano-sheet heterojunction for enhanced water electrolysis activity at high current density. Nano-Micro Lett. **13**, 77 (2021). 10.1007/s40820-021-00607-5 10.1007/s40820-021-00607-5 PMC8187493 34138320

[28] V. Ramalingam, P. Varadhan, H.-C. Fu, H. Kim, D. Zhang et al., Heteroatom-mediated interactions between ruthenium single atoms and an MXene support for efficient hydrogen evolution. Adv. Mater. **31**, e1903841 (2019). 10.1002/adma.201903841 31621970 10.1002/adma.201903841

[29] Y.-L. Zhang, B. Liu, Y. Dai, Y.-F. Xia, P. Guo et al., Electronic delocalization regulates the occupancy and energy level of Co 3d_z2_ orbitals to enhance bifunctional oxygen catalytic activity. Adv. Funct. Mater. **32**, 2209499 (2022). 10.1002/adfm.202209499

[30] H. Gu, W. Yue, J. Hu, X. Niu, H. Tang et al., Asymmetrically coordinated Cu–N_1_C_2_ single-atom catalyst immobilized on Ti_3_C_2_T_*x*_ MXene as separator coating for lithium–sulfur batteries. Adv. Energy Mater. **13**, 2204014 (2023). 10.1002/aenm.202204014

[31] D.A. Kuznetsov, Z. Chen, P.V. Kumar, A. Tsoukalou, A. Kierzkowska et al., Single site cobalt substitution in 2D molybdenum carbide (MXene) enhances catalytic activity in the hydrogen evolution reaction. J. Am. Chem. Soc. **141**, 17809–17816 (2019). 10.1021/jacs.9b08897 31540549 10.1021/jacs.9b08897

[32] H. Liu, Z. Hu, Q. Liu, P. Sun, Y. Wang et al., Single-atom Ru anchored in nitrogen-doped MXene (Ti_3_C_2_T_x_) as an efficient catalyst for the hydrogen evolution reaction at all pH values. J. Mater. Chem. **8**, 24710–24717 (2020). 10.1039/D0TA09538A

[33] H. Zong, S. Gong, K. Yu, Z. Zhu, Ni-doped Ti_3_CNT_x_-coated nanoporous covalent organic frameworks to accelerate hydrogen diffusion for enhanced hydrogen evolution. ACS Appl. Nano Mater. **5**, 15042–15052 (2022). 10.1021/acsanm.2c03218

[34] T.A. Le, Q.V. Bui, N.Q. Tran, Y. Cho, Y. Hong et al., Synergistic effects of nitrogen doping on MXene for enhancement of hydrogen evolution reaction. ACS Sustain. Chem. Eng. **7**, 16879–16888 (2019). 10.1021/acssuschemeng.9b04470

[35] J. Zhu, M. Wang, M. Lyu, Y. Jiao, A. Du et al., Two-dimensional titanium carbonitride MXene for high-performance sodium ion batteries. ACS Appl. Nano Mater. **1**, 6854–6863 (2018). 10.1021/acsanm.8b01330

[36] L. Pu, J. Zhang, N.K.L. Jiresse, Y. Gao, H. Zhou et al., N-doped MXene derived from chitosan for the highly effective electrochemical properties as supercapacitor. Adv. Compos. Hybrid Mater. **5**, 356–369 (2022). 10.1007/s42114-021-00371-5

[37] D. Zhao, Z. Chen, W. Yang, S. Liu, X. Zhang et al., MXene (Ti_3_C_2_) vacancy-confined single-atom catalyst for efficient functionalization of CO_2_. J. Am. Chem. Soc. **141**, 4086–4093 (2019). 10.1021/jacs.8b13579 30699294 10.1021/jacs.8b13579

[38] L. Gao, H. Chen, A.V. Kuklin, S. Wageh, A.A. Al-Ghamdi et al., Optical properties of few-layer Ti_3_CN MXene: from experimental observations to theoretical calculations. ACS Nano **16**, 3059–3069 (2022). 10.1021/acsnano.1c10577 35048704 10.1021/acsnano.1c10577

[39] J. Zhu, L. Xia, R. Yu, R. Lu, J. Li et al., Ultrahigh stable methanol oxidation enabled by a high hydroxyl concentration on Pt clusters/MXene interfaces. J. Am. Chem. Soc. **144**, 15529–15538 (2022). 10.1021/jacs.2c03982 35943197 10.1021/jacs.2c03982

[40] D. Cao, L. Zheng, Y. Wang, Y. Dong, Q. Li et al., Ultraviolet-assisted construction of low-Pt-loaded MXene catalysts for high-performance Li−O_2_ batteries. Energy Storage Mater. **51**, 806–814 (2022). 10.1016/j.ensm.2022.07.026

[41] J. Halim, K.M. Cook, M. Naguib, P. Eklund, Y. Gogotsi et al., X-ray photoelectron spectroscopy of select multi-layered transition metal carbides (MXenes). Appl. Surf. Sci. **362**, 406–417 (2016). 10.1016/j.apsusc.2015.11.089

[42] M. Fan, J. Cui, J. Zhang, J. Wu, S. Chen et al., The modulating effect of N coordination on single-atom catalysts researched by Pt-N_x_-C model through both experimental study and DFT simulation. J. Mater. Sci. Technol. **91**, 160–167 (2021). 10.1016/j.jmst.2021.01.093

[43] Y. Wu et al., Boosting hydrogen evolution in neutral medium by accelerating water dissociation with Ru clusters loaded on Mo_2_CT_x_ MXene. Adv. Funct. Mater. **33**, 2214375 (2023). 10.1002/adfm.202214375

[44] C. Cui, R. Cheng, H. Zhang, C. Zhang, Y. Ma et al., Ultrastable MXene@Pt/SWCNTs’ nanocatalysts for hydrogen evolution reaction. Adv. Funct. Mater. **30**, 2000693 (2020). 10.1002/adfm.202000693

[45] E.P. Beaumier, A.J. Pearce, X.Y. See, I.A. Tonks, Modern applications of low-valent early transition metals in synthesis and catalysis. Nat. Rev. Chem. **3**, 15–34 (2019). 10.1038/s41570-018-0059-x 30989127 10.1038/s41570-018-0059-x PMC6462221

[46] S. Zhang, M.-B. Li, Repurposing HER catalysis toward metal hydride-mediated electro-reductive transformations. Tetrahedron Chem **11**, 100080 (2024). 10.1016/j.tchem.2024.100080

[47] M. Ma, G. Li, W. Yan, Z. Wu, Z. Zheng et al., Single-atom molybdenum engineered platinum nanocatalyst for boosted alkaline hydrogen oxidation. Adv. Energy Mater. **12**, 2103336 (2022). 10.1002/aenm.202103336

[48] H. Jin, M. Ha, M.G. Kim, J.H. Lee, K.S. Kim, Engineering Pt coordination environment with atomically dispersed transition metal sites toward superior hydrogen evolution. Adv. Energy Mater. **13**, 2204213 (2023). 10.1002/aenm.202204213

[49] Z. Sun, Y. Yang, C. Fang, Y. Yao, F. Qin et al., Atomic-level Pt electrocatalyst synthesized *via* iced photochemical method for hydrogen evolution reaction with high efficiency. Small **18**, e2203422 (2022). 10.1002/smll.202203422 35871552 10.1002/smll.202203422

[50] D.-D. Qin, Y. Tang, G. Ma, L. Qin, C.-L. Tao et al., Molecular metal nanoclusters for ORR, HER and OER: achievements, opportunities and challenges. Int. J. Hydrog. Energy **46**, 25771–25781 (2021). 10.1016/j.ijhydene.2021.05.096

[51] K. Kwak, W. Choi, Q. Tang, D. Jiang, D. Lee, Rationally designed metal nanocluster for electrocatalytic hydrogen production from water. J. Mater. Chem. **6**, 19495–19501 (2021). 10.1039/C8TA06306K

[52] Y. Zhao, X. Tan, X. Tan, W. Yang, C. Jia et al., Surface reconstruction of ultrathin palladium nanosheets during electrocatalytic CO_2_ reduction. Angew. Chem. Int. Ed. **59**, 21493–21498 (2020). 10.1002/anie.202009616 10.1002/anie.202009616 32715613

