# Correction: Transition Metal Carbonitride MXenes Anchored with Pt Sub-nanometer Clusters to Achieve High-Performance Hydrogen Evolution Reaction at All pH Range

**DOI:** 10.1007/s40820-025-01690-8

**Published:** 2025-03-03

**Authors:** Zhihao Lei, Sajjad Ali, CI Sathish, MuhammadIbrar Ahmed, Jiangtao Qu, Rongkun Zheng, Shibo Xi, Xiaojiang Yu, M. B. H. Breese, Chao Liu, Jizhen Zhang, Shuai Qi, Xinwei Guan, Vibin Perumalsamy, Mohammed Fawaz, Jae-Hun Yang, Mohamed Bououdina, Kazunari Domen, Ajayan Vinu, Liang Qiao, Jiabao Yi

**Affiliations:** 1 Global Innovative Center of Advanced Nanomaterials, College of Engineering, Science and Environment, University of Newcastle, Callaghan, NSW 2308 Australia; 2 Energy, Water, and Environment Lab, College of Humanities and Sciences, Prince Sultan University, 11586 Riyadh, Saudi Arabia; 3 School of Physics, University of Sydney, Sydney, NSW 2000 Australia; 4 Institute of Chemical and Engineering Sciences, A*STAR, Singapore, 627833 Singapore; 5 Singapore Synchrotron Light Source, National University of Singapore, Singapore, 117603 Singapore; 6 Institute for Frontier Materials, Deakin University, Waurn Ponds, Victoria 3216 Australia; 7 Guangdong Provincial Key Laboratory of Natural Rubber Processing, Agricultural Products Processing Research Institute, Chinese Academy of Tropical Agricultural Sciences, Zhanjiang, 524001 People’s Republic of China; 8 College of Chemistry Environmental Engineering, Shenzhen University, Shenzhen, 518060 Guangdong People’s Republic of China; 9 Research Initiative for Supra-Materials Interdisciplinary Cluster for Cutting Edge Research, Shinshu University, 4-17-1, Wakasato, Nagano-shi, Nagano 380-8533 Japan; 10 School of Physics, University of Electronic Science and Technology of China, Chengdu, 610054 People’s Republic of China; 11 Department of Chemical Engineering and Interdisciplinary Research Center for Hydrogen Technologies and Carbon Management (IRC-HTCM), King Fahd University of Petroleum and Minerals, 31261 Dhahran, Saudi Arabia

**Correction to: Nano-Micro Letters (2025) 17:123** 10.1007/s40820-025-01654-y

Following publication of the original article [1], the authors reported that Dr. Mohamed Bououdina’s affiliation needed to be corrected from 1 to 2.

The correct author affiliation has been provided in this Correction and the original article [1] has been corrected.
