# Supplementary material for: Transition Metal Carbonitride MXenes Anchored with Pt Sub-Nanometer Clusters to Achieve High-Performance Hydrogen Evolution Reaction at All pH Range
Source: Nanomicro Lett. 2025 Jan 31;17:123. doi: 10.1007/s40820-025-01654-y (PMC11785901; doi:10.1007/s40820-025-01654-y)
Supplement: Supplementary file 1 — Supplementary file1 (DOCX 8651 KB) [file 40820_2025_1654_MOESM1_ESM.docx]

Supporting Information for

**Transition Metal Carbonitride MXenes Anchored with Pt Sub-Nanometer Clusters to Achieve High-Performance Hydrogen Evolution Reaction at All pH Range**

Zhihao Lei^1^, Sajjad Ali^2,3^ , CI Sathish^1^, Muhammad Ibrar Ahmed^1^, Jiangtao Qu^4^, Rongkun Zheng^4^, Shibo Xi^5^, Xiaojiang Yu^6^, M.B.H. Breese^6^, Chao Liu^7^, Jizhen Zhang^7,8^, Shuai Qi^9^, Xinwei Guan^1^, Vibin Perumalsamy^1^, Mohammed Fawaz^1^, Jae-Hun Yang^1^, Mohamed Bououdina ^3^, Kazunari Domen^10^, Ajayan Vinu^1^, Liang Qiao^2^*, Jiabao Yi^11^*

^1^ Global Innovative Center of Advanced Nanomaterials, College of Engineering, Science and Environment, University of Newcastle, Callaghan, 2308, NSW, Australia

^2^ School of Physics, University of Electronic Science and Technology of China, Chengdu 610054, P. R. China

^3^ Energy, Water, and Environment Lab, College of Humanities and Sciences, Prince Sultan University, Riyadh 11586, Saudi Arabia

^4^ School of Physics, University of Sydney, NSW, 2000, Australia

^5^ Institute of Chemical and Engineering Sciences, A*STAR, Singapore, 627833 Singapore

^6^ Singapore Synchrotron Light Source, National University of Singapore, Singapore 117603

^7^ Institute for Frontier Materials, Deakin University, Waurn Ponds, Victoria, 3216, Australia

^8^ Guangdong Provincial Key Laboratory of Natural Rubber Processing, Agricultural Products Processing Research Institute, Chinese Academy of Tropical Agricultural Sciences, Zhanjiang, 524001, P. R. China

^9^ College of Chemistry Environmental Engineering, Shenzhen University, Shenzhen, Guangdong, 518060, P. R. China

^10^Research Initiative for Supra-Materials Interdisciplinary Cluster for Cutting Edge Research, Shinshu University4-17-1, Wakasato, Nagano-shi, Nagano 380-8533, Japan

^11^Department of Chemical Engineering, King Fahd University of Petroleum & Minerals, Dhahran 31261, Saudi Arabia

*Corresponding authors. E-mail: [Jiabao.yi@kfupm.edu.sa](mailto:Jiabao.yi@kfupm.edu.sa) (Jiabao Yi); [liang.qiao@uestc.edu.cn](mailto:liang.qiao@uestc.edu.cn) (Liang Qiao)

**S1 Experimental Section**

**S1.1 Chemicals and Materials**

All the chemicals used in this work were purchased from suppliers without any further purification. Ti_3_AlCN MAX powder ($\geq$99 wt%, 200 mesh) was purchased from Nanjin Muke, China and all other chemicals including lithium fluoride (LiF, 99.9% metals basis), hydrochloric acid (HCl, 37% in aq. solution), chloroplatinic acid hexahydrate (H_2_PtCl_6_·6H_2_O, ACS, Pt 37.5% min), potassium hydroxide (KOH, 99.99% metals basis), potassium sulfate (K_2_SO_4_, 99.99% metals basis), sulfuric acid, polyvinylidene fluoride (PVDF, 99%), and platinum on graphitized carbon (Pt/C,20%) were purchased from Sigma Aldrich.

**S1.2 Synthesis of Ti_3_CNT_x_ MXene nanosheets**

1g of Ti_3_AlCN MAX powders were slowly added to the special etching solution that was obtained by mixing 1.6 g lithium fluoride (LiF, 99%, Sigma-Aldrich Pty Ltd) and 20 ml of 9 M hydrochloric acid (HCl). The etching was completed after stirring for 24 h at 35 °C. The resulting products were collected after washing and centrifuging several times with deionized water until the pH value of supernatant reached ~6. The as-obtained precipitate was then redispersed in deionized water to conduct self-delamination via continuous hand shaking. The dispersion was centrifuged to collect the precipitate and redispersed in deionized water again. After repeating the centrifugation and redispersion for several times, the dark supernatant colloidal suspension containing uniformly distributed ultrathin Ti_3_CNT_x_ nanosheets was procured. The concentration of this MXene suspension was approximately 10 mg/mL. A certain amount of MXene suspension were directly freeze-dried to obtain pure Ti_3_CNT_x_ nanosheets.

**S1.3 Preparation of Pt-Ti_3_CNT_x_ catalysts**

In a typical synthesis of Pt-Ti_3_CNT_x_, 0.25 mL of H_2_PtCl_6_·6H_2_O solution (10 mg/mL) was slowly dribbled into the aqueous solution containing 50 mg Ti_3_CNT_x_. The control experiment group was designed by only changing the content of H_2_PtCl_6_·6H_2_O into 0.5, 0.75, 1.0, 1.25 and 1.5 mL. The mixed solution was stirred for half an hour and sonicated for another half hour at low temperature. The as-produced precursor was stored at -20 °C for 48 h and then freeze-dried for 48 h to gain Pt-Ti_3_CNT_x_ samples. These Pt-Ti_3_CNT_x_ samples were denoted as Pt-MXene-2.5 (0.25 mL), Pt-MXene-5 (0.5 mL), Pt-MXene-7.5 (0.75 mL), Pt-MXene-10 (1 mL), Pt-MXene-12.5 (1.25 mL) and Pt-MXene-15 (1.5 mL), respectively.

**S1.4 Materials characterization**

The XRD patterns were collected using the instrument Empyrean from Panalytical, CuKα1, λ = 1.5406 Å and CuKα2, λ = 1.5444 Å, recorded in 2θ from 5° to 80°. The surface morphologies were taken under the SEM (JSM-7900 F, JEOL). It is to note that the MXene solution will be directly dropped on silicon substrate for SEM analysis. Hence, the morphologies will be different from that for electrode, where the MXene nanosheets agglomerate each other. The AFM images were obtained with the help of AIST-NT Smart SPM. The high-resolution TEM images and corresponding elemental mapping were measured using JEOL JEM-F200 Multi-Purpose FEG-S/TEM. Besides, the element content was recorded using different techniques like EDS attached to the SEM system and X-ray photoelectron spectroscopy. XPS (Thermo Scientific) was employed to investigate the composition of samples and elemental valence state via an Al Kα X-ray source (1486.7 eV) radiation source at high vacuum and room temperature. X-ray absorption near-edge structure (XANES) and extended X-ray absorption fine structure (EXAFS) analyses were carried out in Singapore Synchrotron Light Source (SSLS).

**S1.5 Electrochemical measurements**

A three-electrode system was employed to perform the electrochemical activity tests via an electrochemical station (Princeton, PARSTAT 4000 Rear Panel). The counter electrode (CE) and reference electrode (RE) were a graphite rod and a KCl-saturated Ag/AgCl electrode, respectively. The synthesized catalysts, together with Super P and binder (PVDF dissolved in N-Methyl pyrrolidone) were mixed in a mass ratio of 7:2:1 and ground to obtain a catalyst slurry. The working electrode (WE) was then made of 1 × 1 cm^2^ of nickel foam coated with the slurry (The loaded mass was approximately 1.5 mg cm^-2^). All the potentials in this work were converted relative to reversible hydrogen electrode (RHE) based on the Nernst equation, namely, E_RHE_ = E_Ag/AgCl_ + 0.198+ 0.0591 × pH. Linear sweep voltammetry (LSV) with the scan rate set at 5 mV S^-1^ was chosen to measure the polarization curves, which was subsequently compensated using iR. Electrochemical surface area (ECSA) was measured through cyclic voltammograms (CV) under different scan rates (10, 20, 40, 60, 80, 100 mV S^-1^) within the Faradaic potential window (0.42-0.62 V vs. RHE). The electrochemical impedance spectroscopy (EIS) was obtained with a frequency ranging from 0.1 to10^5^ kHz and an amplitude set at 10 mV. The HER activity testes were assessed in acidic, neutral and alkaline electrolytes, namely, 0.5 M H_2_SO_4_, 0.5 M K_2_SO_4_ and 1 M KOH.

**S2 Computational Methods**

The density functional theory (DFT) calculations were performed by using the projector-augmented wave (PAW) method via the Vienna ab initio simulation Package (VASP). The Perdew-Burke-Ernzerhof generalized gradient approximation (PBE-GGA) was employed to describe the exchange–correlation potentials [S1, S2]. For structure optimization, 3×3×1 k-points mesh and 450 eV energy cutoff have been used [S3]. The DFT-D3 type of van der Waals correction are considered in the DFT calculations [S4]. The energy convergence criteria were set to 1 × 10^−5^ eV to get accurate forces, and a force tolerance of -0.02 eV/Å was used in structural optimization.

The hydrogen evolution reaction (HER) proceeds via one-electron transfer mechanism and includes one proton-electron coupled elementary steps:

𝐻^+^ + 𝑒^−^+ ∗ → 𝐻* (S1)

𝐻* → $\frac{1(H2)}{2}$ + ∗ (S2)

where ∗ indicates active site, and 𝐻* is the H adsorption. The zero-point energy, enthalpy, and entropy corrections were added to 𝐻* to convert electronic energy of 𝐻* to free energy.

The Gibbs free energy (G) at U=0 V is modified by Equation (S3).

G = 𝐸_𝐷𝐹𝑇_ + 𝐸_𝑍𝑃𝐸_ – 𝑇𝑆 + ∫C_p_d𝑇 (S3)

where 𝐸_𝐷𝐹𝑇_, 𝐸_𝑍𝑃𝐸_, 𝑇𝑆, and ∫C_p_d𝑇 represent electronic energy, zero-point energy room temperature entropy and heat capacity (i.e., T=298.15 K). The Gibbs free energy was calculated using the computational hydrogen electrode (CHE) model. In CHE model, the reaction step proton-electron coupled and the G of electron-proton pairs (e^−^ + H^+^) was calculated as a function of applied potential (U) relative to a reversible hydrogen electrode (RHE) [S5], i.e., μ(H^+^) + μ(e^−^) = $\frac{1}{2}$ μ(H_2_) – eU. The G of the adsorbed hydrogen ∆(H*) is a key descriptor for the HER catalytic activity, and ∆𝐺(H*) at a given potential 𝑈_𝑅𝐻𝐸_ can be written as:

∆(H*) = 𝐺(H*) ‒ 𝐺(∗) ‒ $\frac{1G(H2)}{2}$ ‒ 𝑒𝑈_𝑅_ (S4)

**S3 TOF Calculations**

To calculate the turnover frequency (TOF) per Pt site of the Pt-MXene samples, the following equation is used:

$$TOF=\frac{number of the total hydrogen turnovers/\mathrm{cm}^{2} of geometric area}{number of active sites/\mathrm{cm}^{2} of geometric area}=\frac{i\times\frac{1H_{2}}{2e^{-}}\times\frac{1e^{-}}{q_{e}}}{N_{\mathrm{active}}}$$

The total number of hydrogen turnovers per geometric area (${H_{2}}^{*}$) can be calculated from the current density, as shown below:

$${H_{2}}^{*}=\left( j\frac{\mathrm{mA}}{\mathrm{cm}^{2}} \right)\left( \frac{1Cs^{-1}}{1000 mA} \right)\left( \frac{1 mol e^{-}}{96485 C} \right)\left( \frac{1 mol H_{2}}{2 mol e^{-}} \right)\left( \frac{6.022\times{10}^{23}\mathrm{sites}}{1 mol} \right)$$

$$=J\times3.12\times{10}^{15}\frac{{H_{2}}/s}{\mathrm{cm}^{2}}\mathrm{per}\frac{\mathrm{mA}}{\mathrm{cm}^{2}}$$

The Pt content of Pt-MXene-12.5 measured by ICP-MS is 4.2 wt%. The mass loading of the catalyst on the electrode is ~ 1.0 mg/cm^2^. Thus, the number of active sites ($N_{\mathrm{active}}$) is calculated according to the following equation:

$$N_{\mathrm{active}}=\left( \frac{4.2\%\times1.0\frac{\mathrm{mg}}{\mathrm{cm}^{2}}}{195.08\frac{g}{\mathrm{mol}}} \right)\left( \frac{6.022\times{10}^{23}\mathrm{sites}}{1 mol} \right)=1.3\times{10}^{17} Pt sites/\mathrm{cm}^{2}$$

$$TOF=\frac{\left| J \right|\times3.12\times{10}^{15}\frac{{H_{2}}/s}{\mathrm{cm}^{2}}\mathrm{per}\frac{\mathrm{mA}}{\mathrm{cm}^{2}}}{N_{\mathrm{active}}}$$

Likewise, the number of active sites and the corresponding turnover frequency per Pt site of the Pt/C (20%) samples is calculated as follows:

$$N_{\mathrm{active}}^{Pt/C}=\left( \frac{20\%\times1.0\frac{\mathrm{mg}}{\mathrm{cm}^{2}}}{195.08\frac{g}{\mathrm{mol}}} \right)\left( \frac{6.022\times{10}^{23}\mathrm{sites}}{1 mol} \right)=6.17\times{10}^{17} Pt sites/\mathrm{cm}^{2}$$

$$\mathrm{TOF}_{Pt/C}=\frac{\left| J \right|\times3.12\times{10}^{15}\frac{{H_{2}}/s}{\mathrm{cm}^{2}}\mathrm{per}\frac{\mathrm{mA}}{\mathrm{cm}^{2}}}{N_{\mathrm{active}}^{Pt/C}}$$

**Supplementary Figures and Tables**


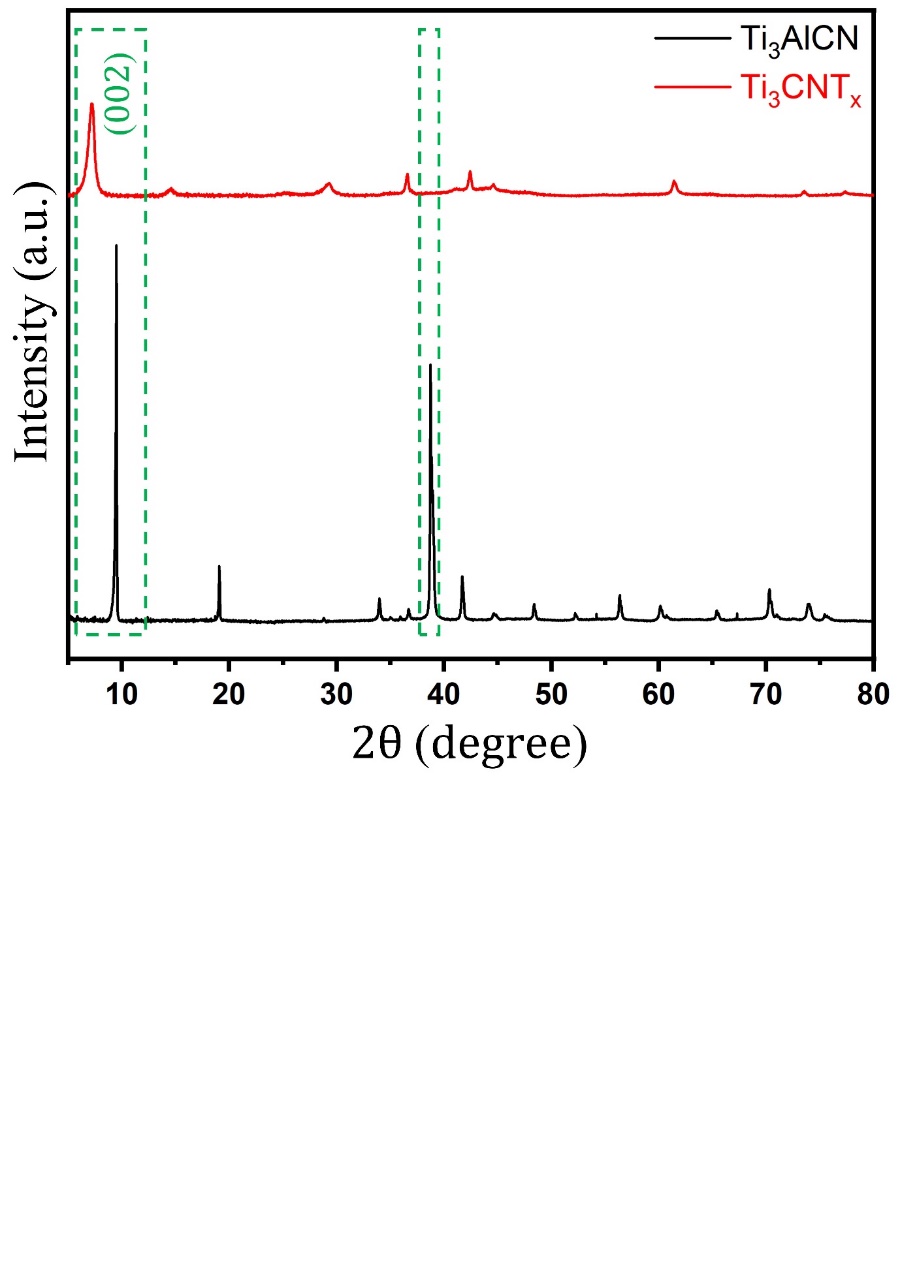


**Fig. S1** XRD patterns of Ti_3_AlCN and Ti_3_CNT_x_


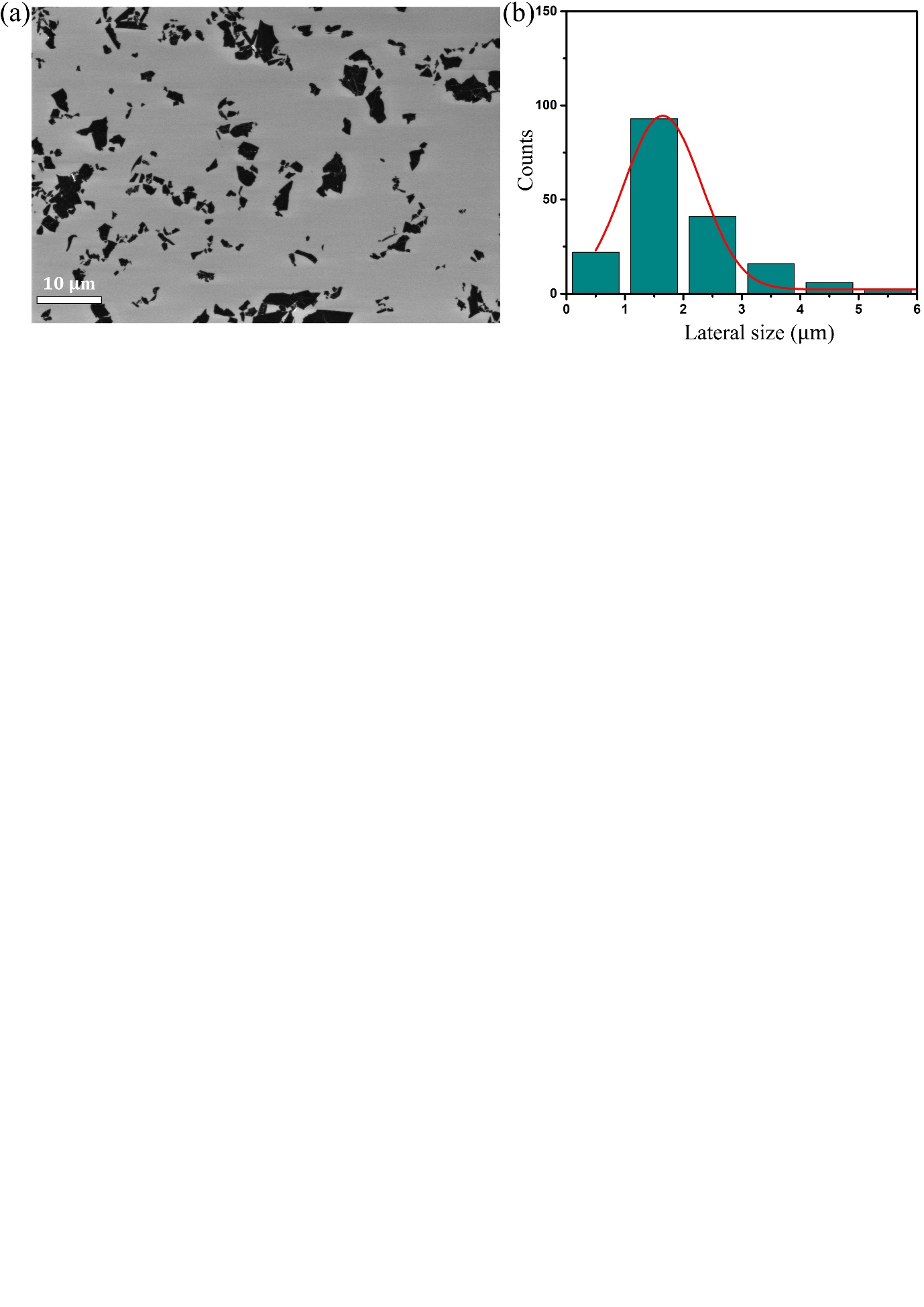


**Fig. S2** (**a**) SEM image of well-dispersed Ti_3_CNT_x_ nanosheets. (**b**) The summarized lateral size distribution of SEM image


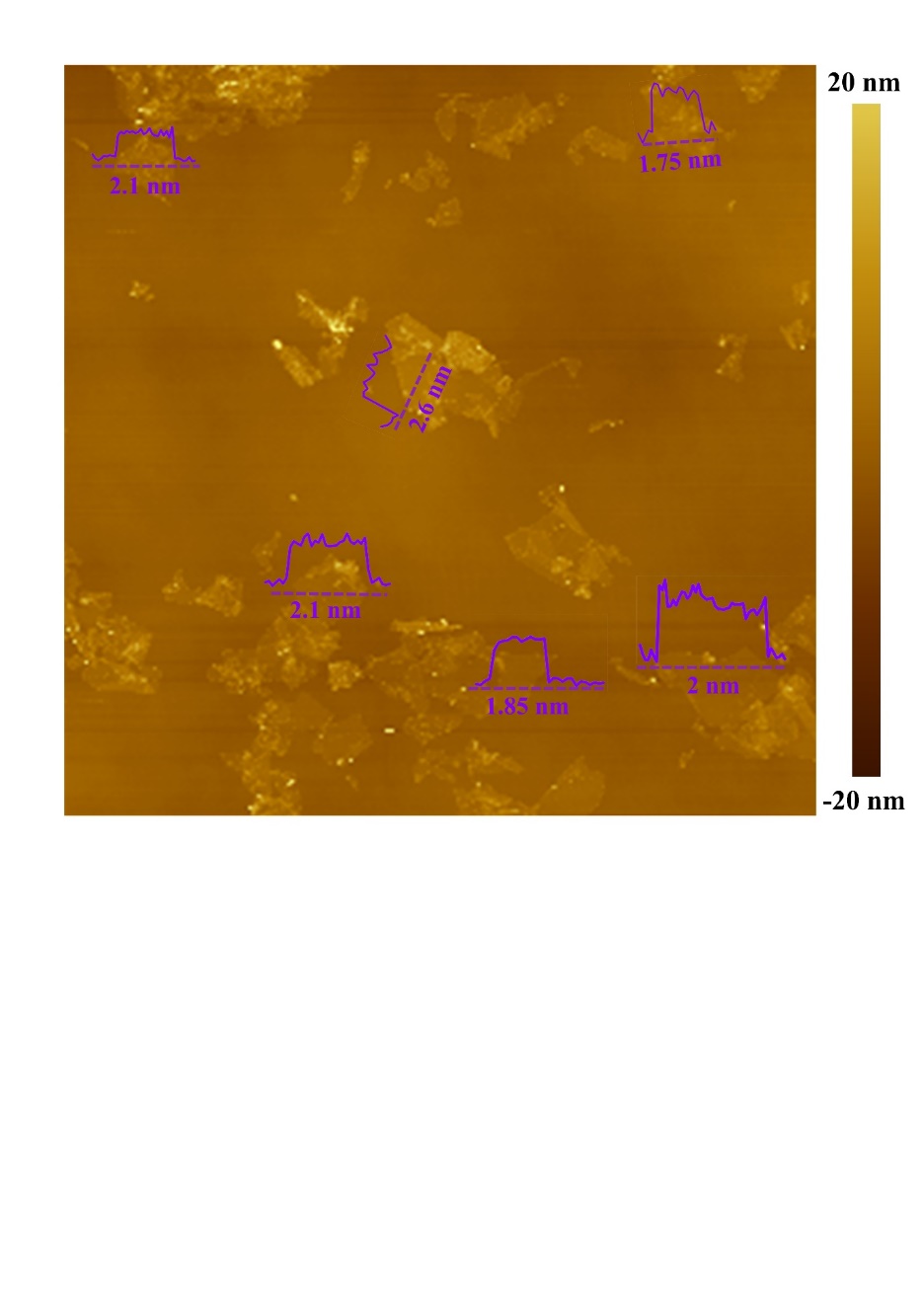


**Fig. S3** AFM image of the atomically thin Ti_3_CNT_x_ nanosheets


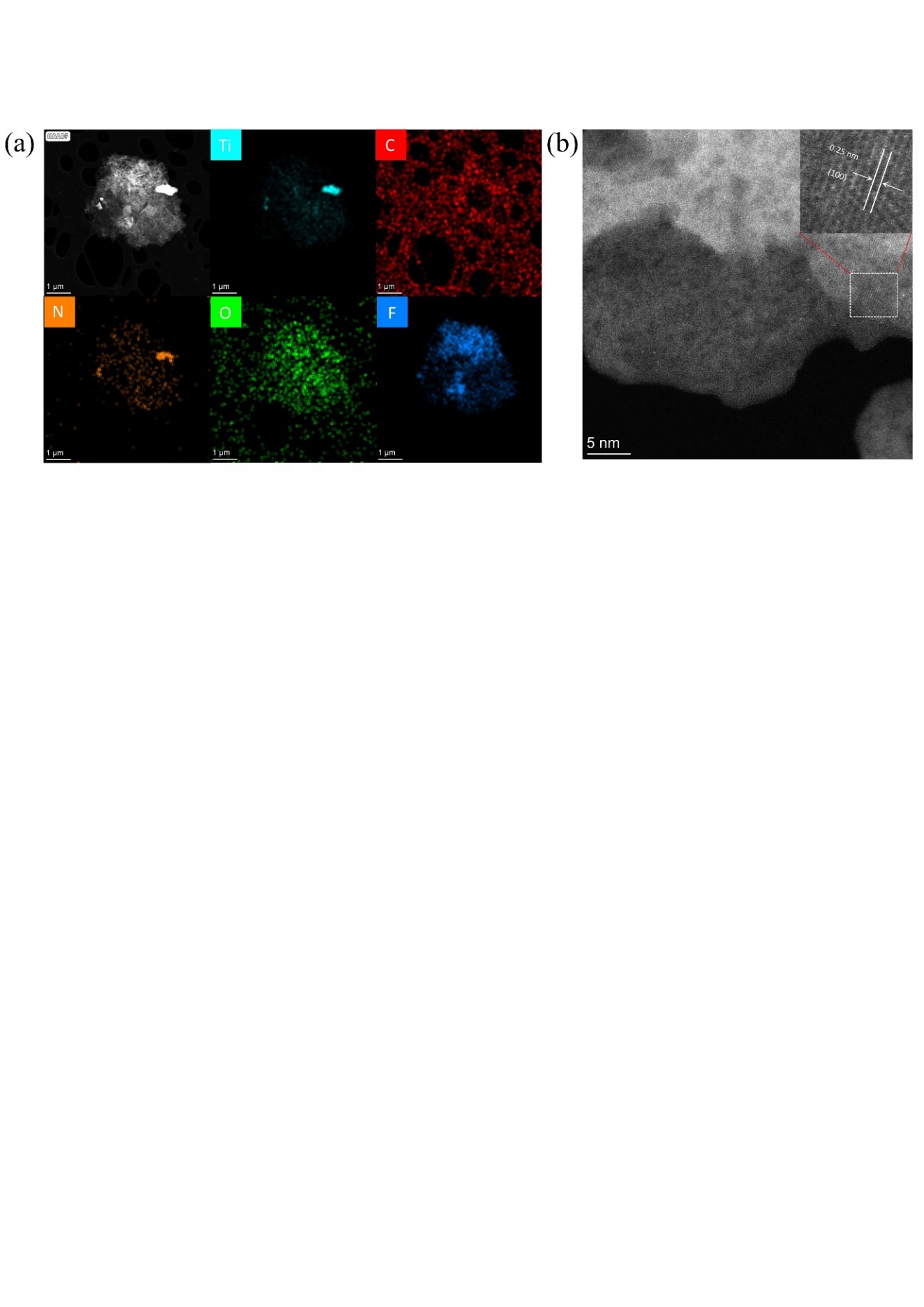


**Fig. S4** (**a**) TEM image of the stacked few-layer Ti_3_CNT_x_ nanosheets after freeze-drying and the corresponding EDS mapping. (**b**) The high-resolution TEM image of Ti_3_CNT_x_ nanosheets. Inset: the magnified lattice fringe of Ti_3_CNT_x_ showing (100) crystal plane


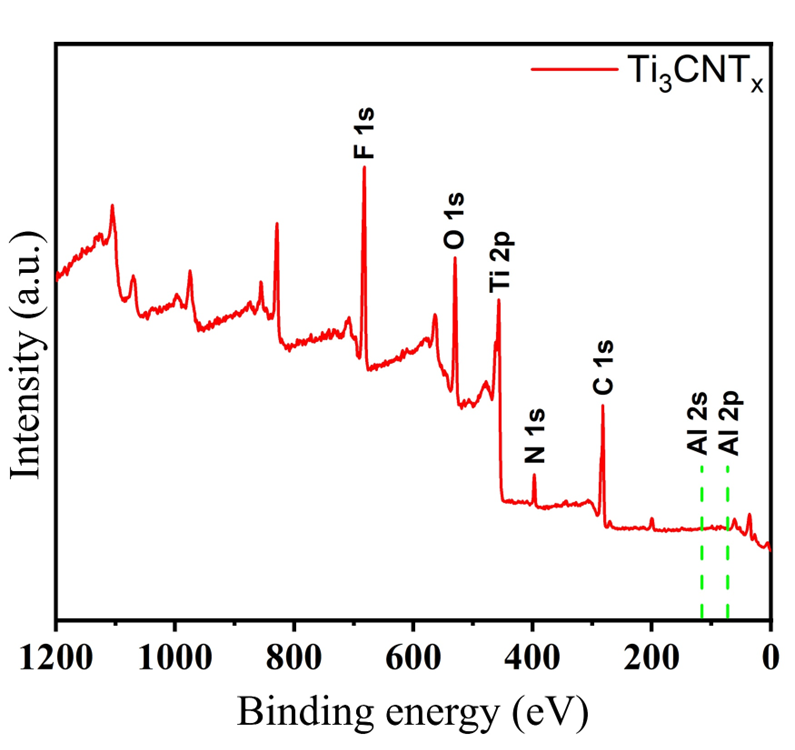


**Fig. S5** XPS survey spectrum of Ti_3_CNT_x_ MXene


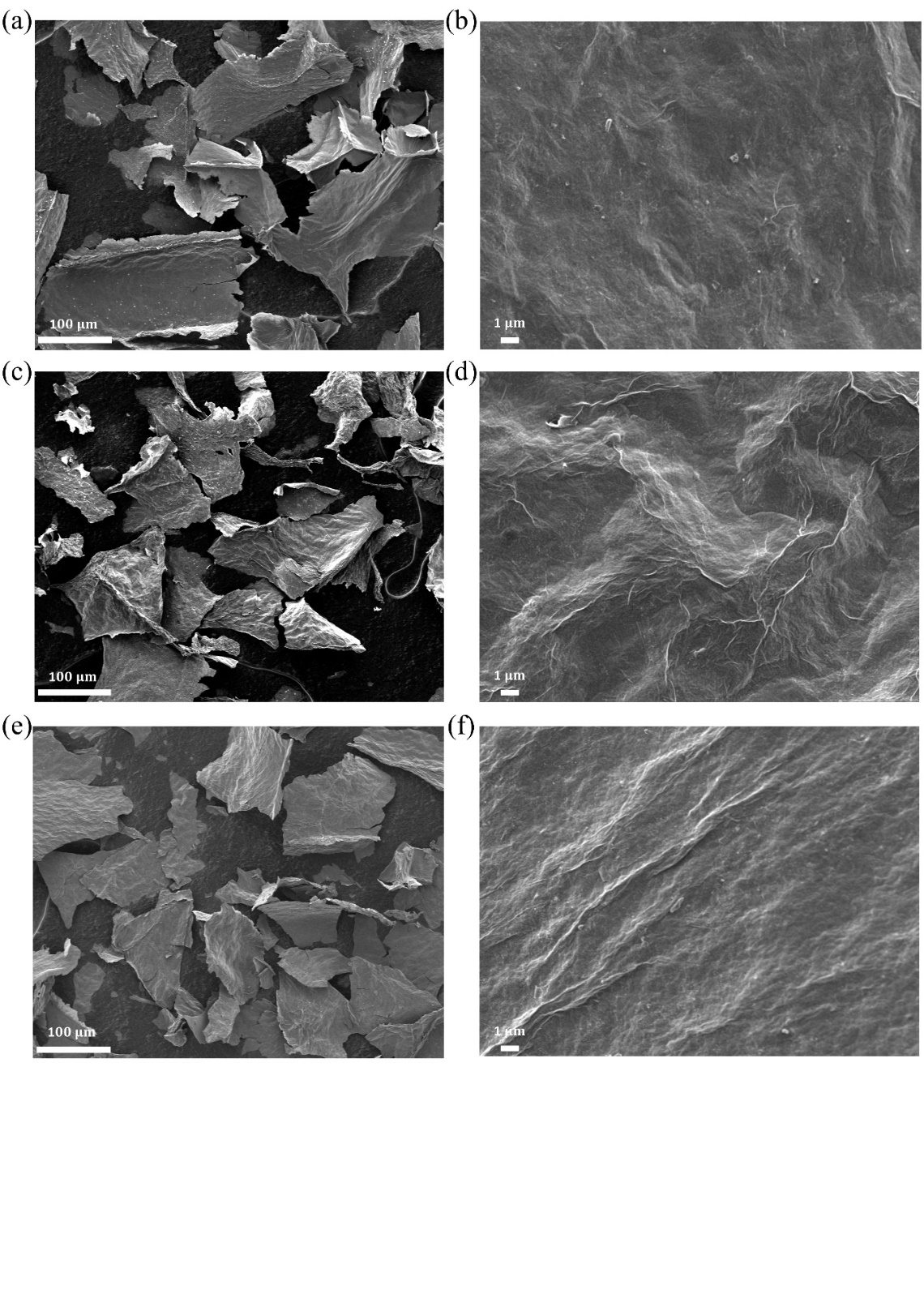


**Fig. S6** (**a, c, e**) Low-magnified and (**b, d, f**) high-magnified SEM images of Pt-MXene after freeze drying for the sample Pt-MXene-10, Pt-MXene-12.5 and Pt-MXene-15


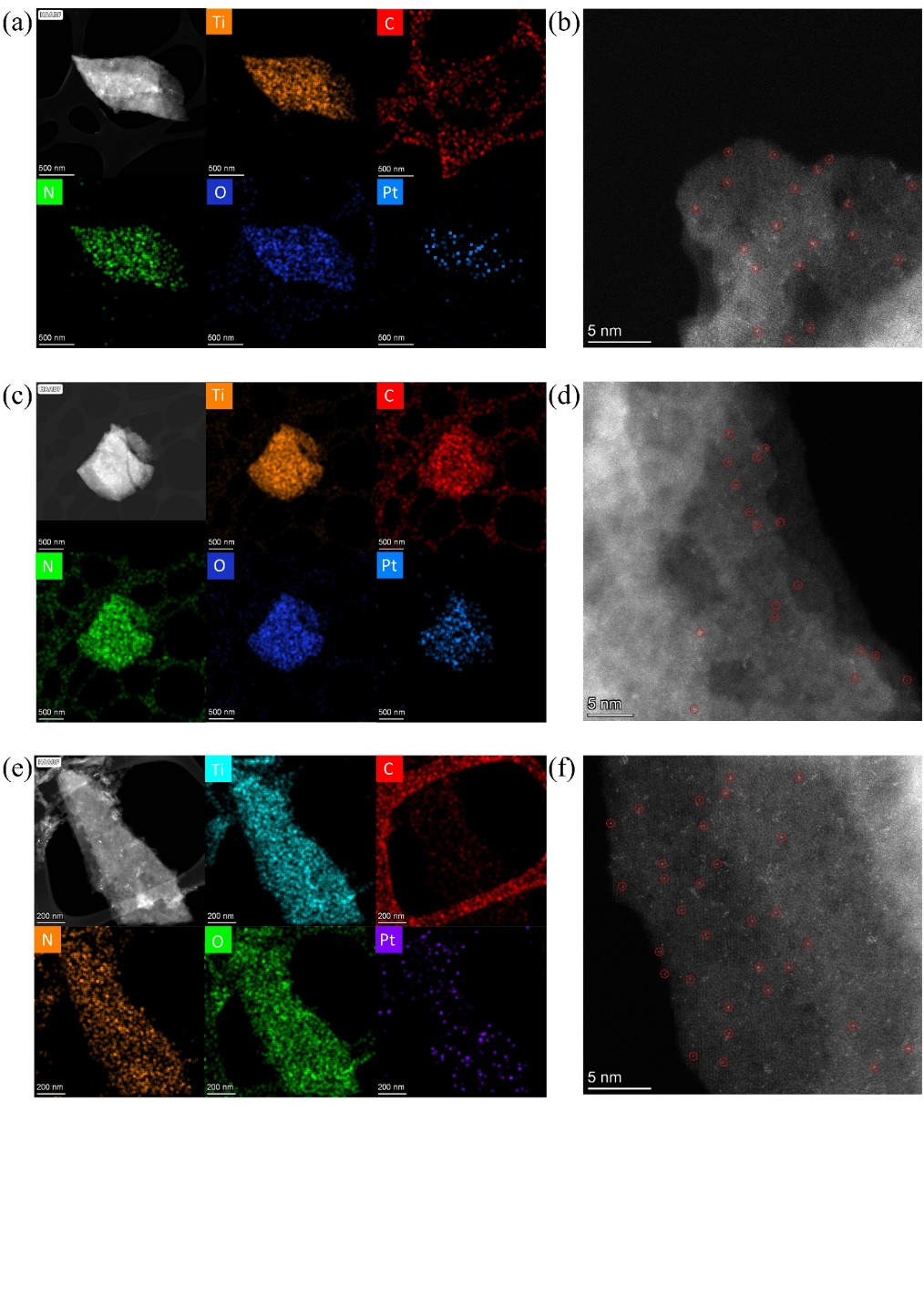


**Fig. S7.** TEM images, the corresponding EDS mapping and HR-TEM images of Pt-MXene-2.5 (**a, b**), Pt-MXene-5 (**c, d**), Pt-MXene-7.5 (**e, f**)


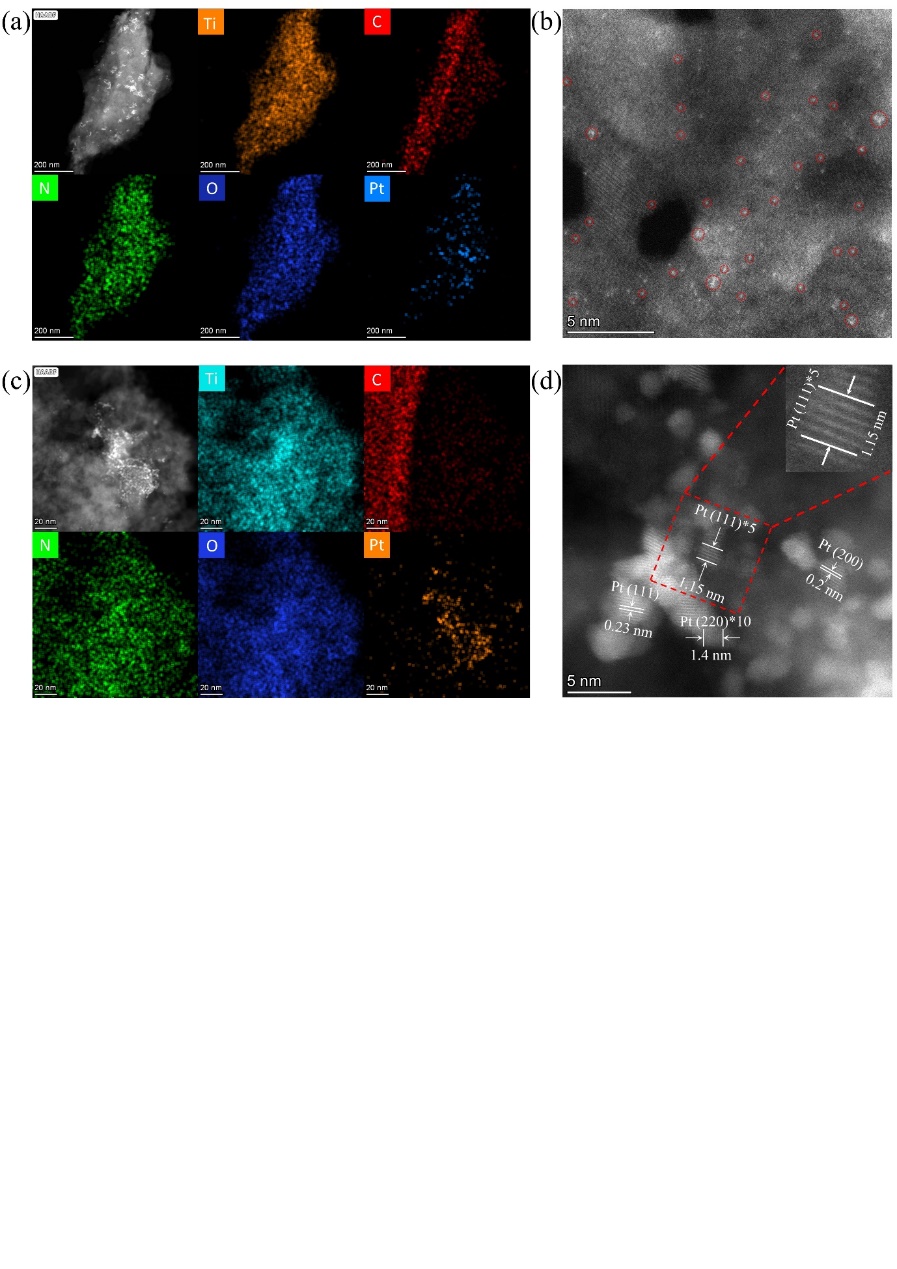


**Fig. S8** TEM images, the corresponding EDS mapping and HR-TEM images of Pt-MXene-10 (**a, b**) and Pt-MXene-15 (**c, d**)


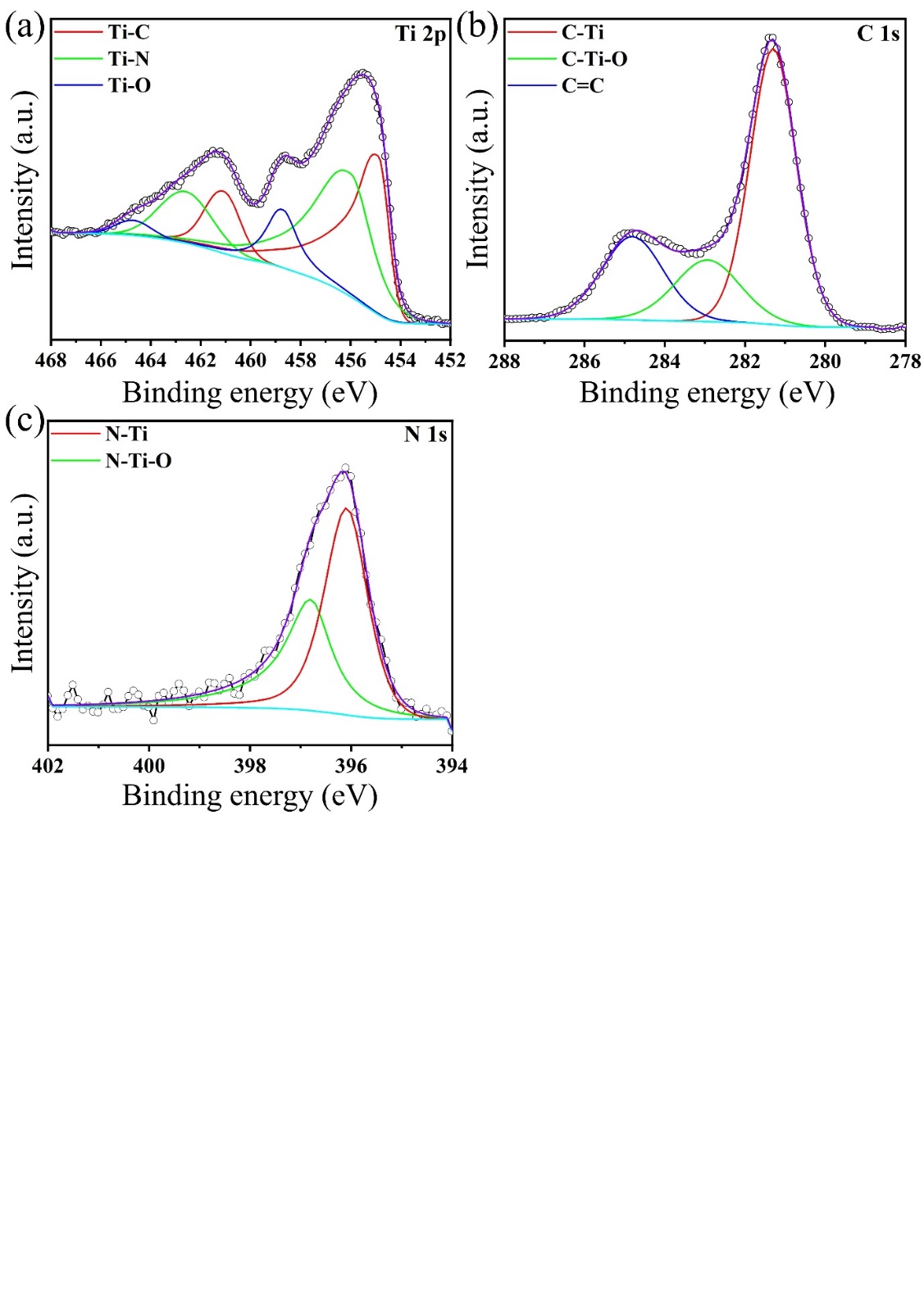


**Fig. S9** The high-resolution (a) Ti 2p, (b) C 1s and (c) N 1s spectra of pure MXene sample


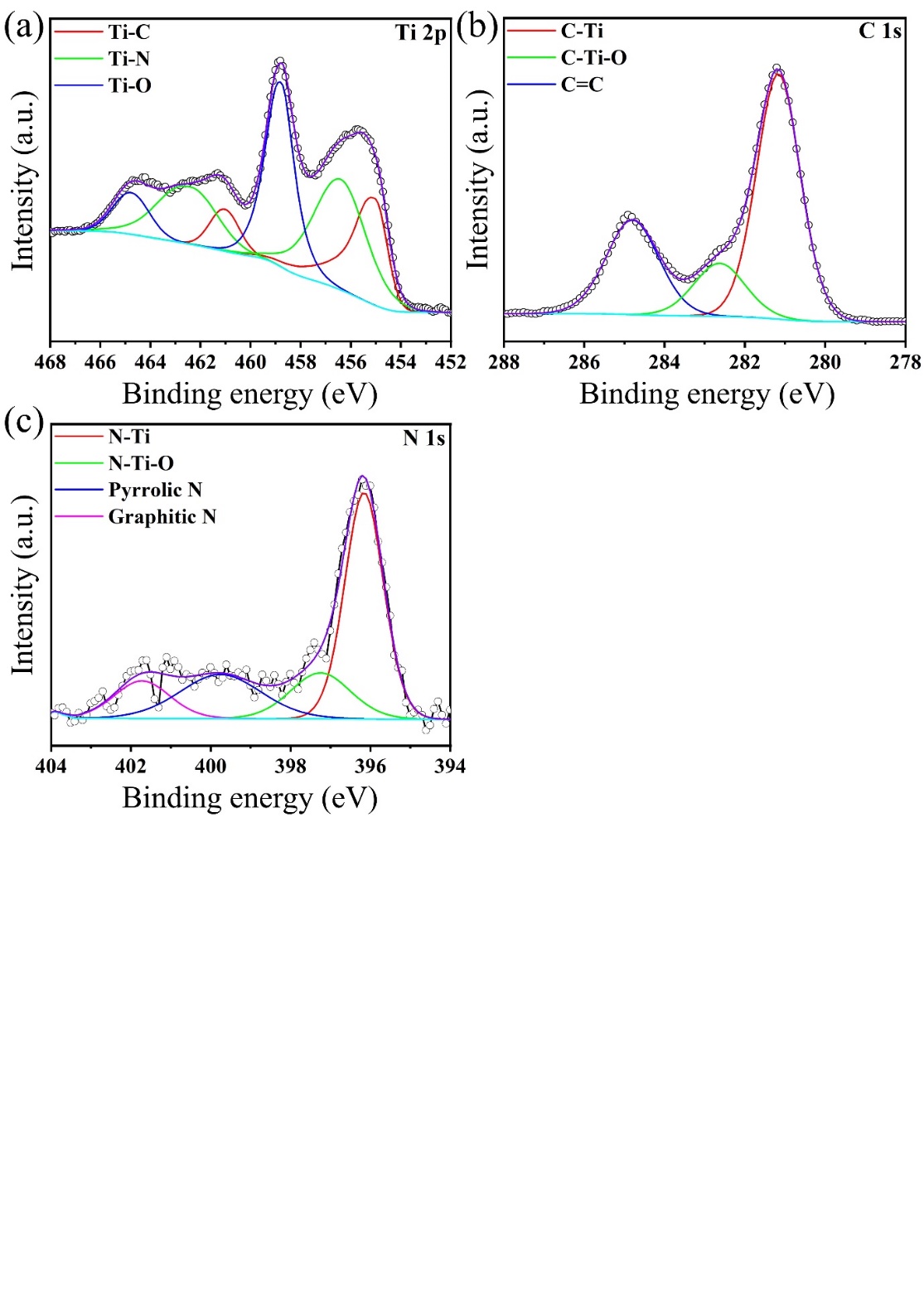


**Fig. S10** The high-resolution (a) Ti 2p, (b) C 1s and (c) N 1s spectra of Pt-MXene-2.5 sample


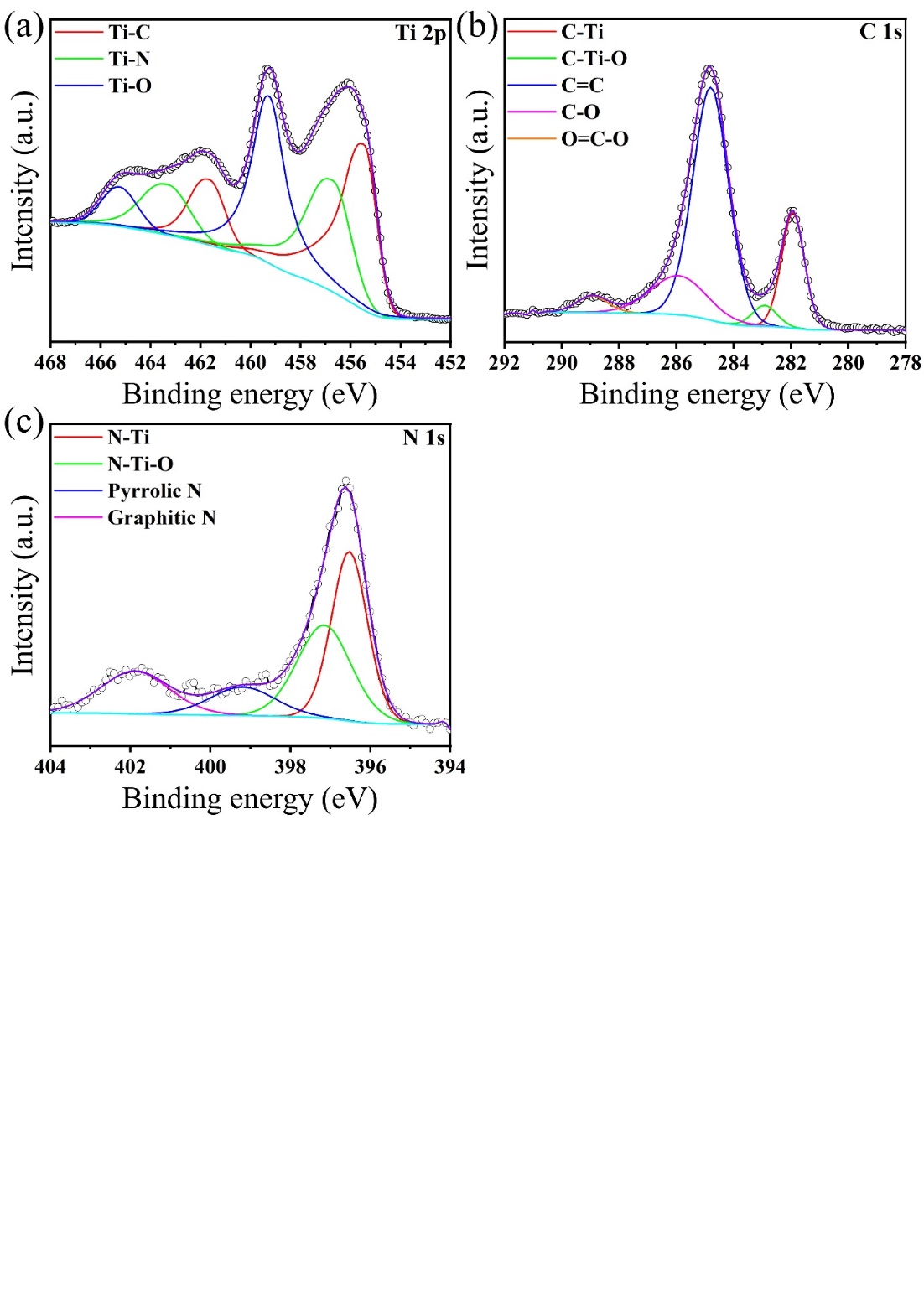


**Fig. S11** The high-resolution (**a**) Ti 2p, (**b**) C 1s and (**c**) N 1s spectra of Pt-MXene-5 sample


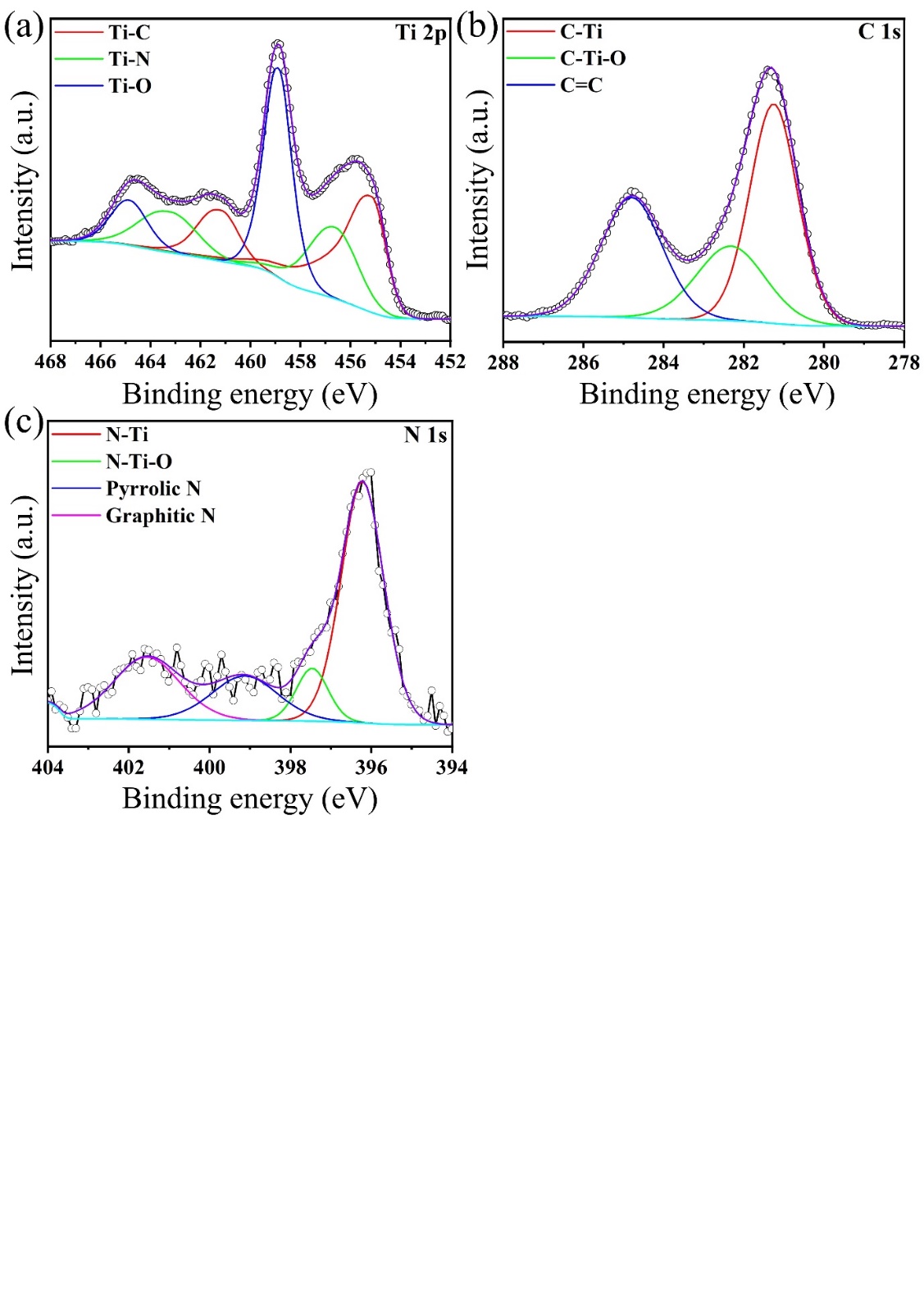


**Fig. S12** The high-resolution (**a**) Ti 2p, (**b**) C 1s and (**c**) N 1s spectra of Pt-MXene-7.5 sample


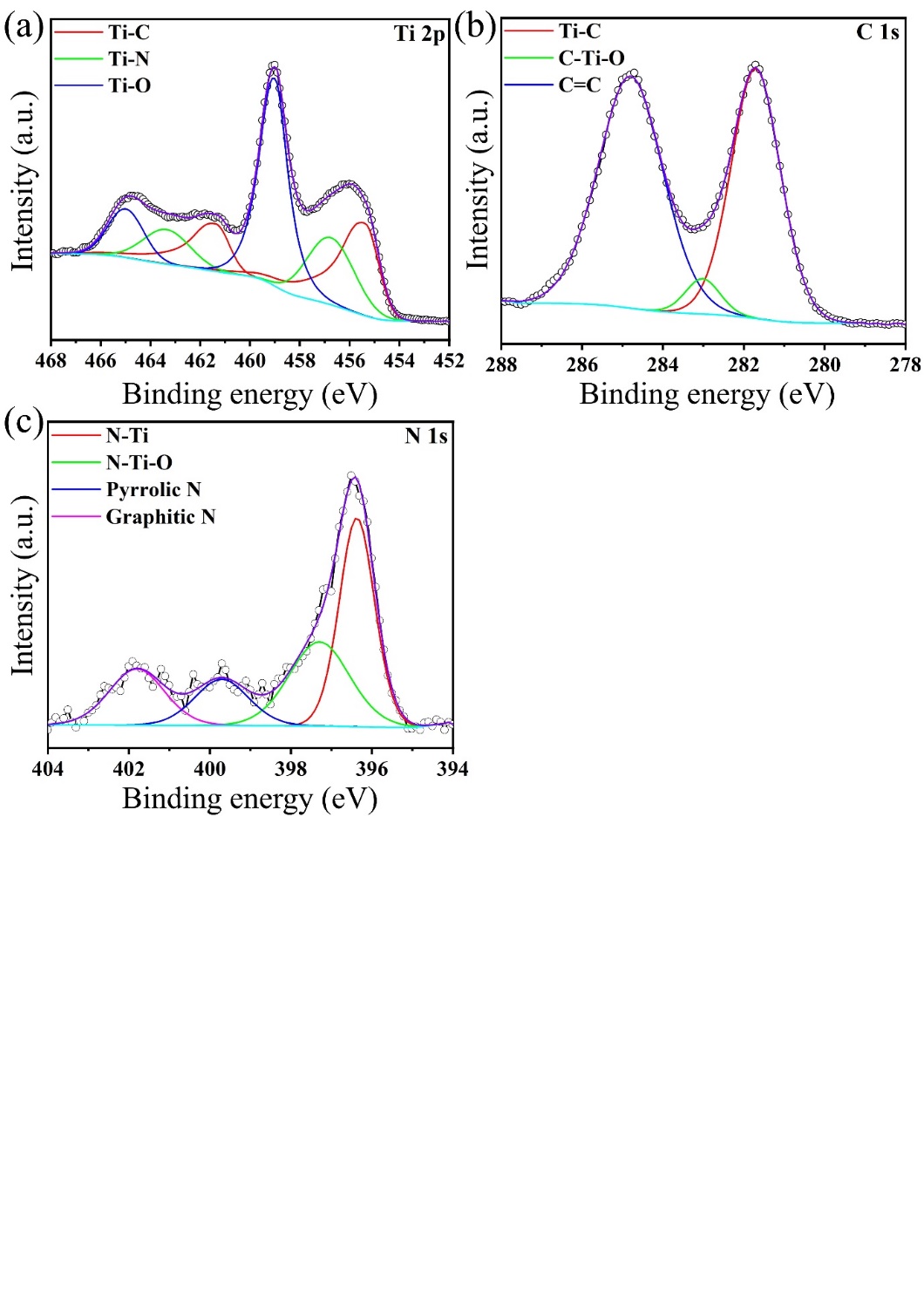


**Fig. S13** The high-resolution (**a**) Ti 2p, (**b**) C 1s and (**c**) N 1s spectra of Pt-MXene-10 sample


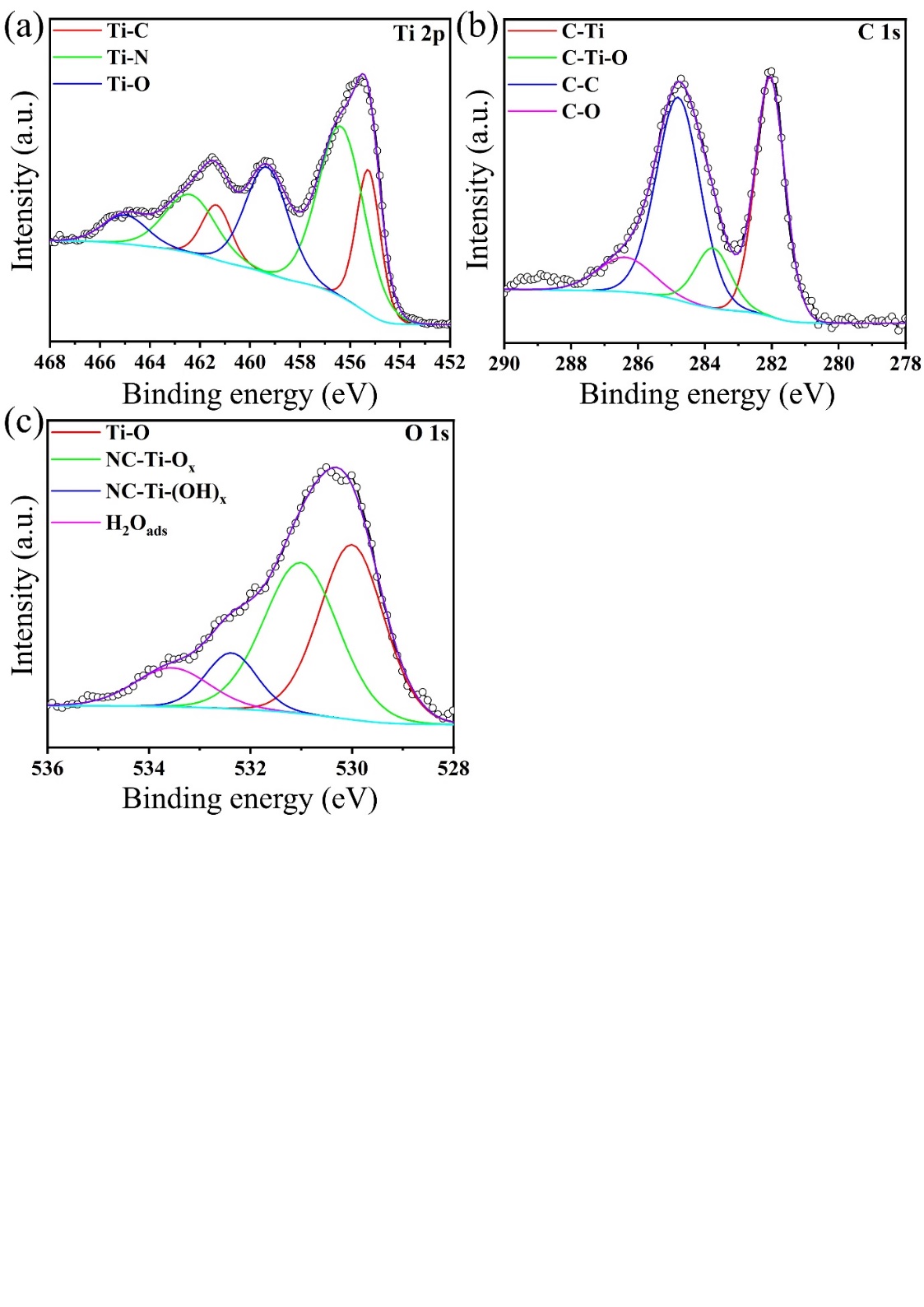


**Fig. S14** The high-resolution (**a**) Ti 2p, (**b**) C 1s and (**c**) O 1s spectra of Pt-MXene-15 sample


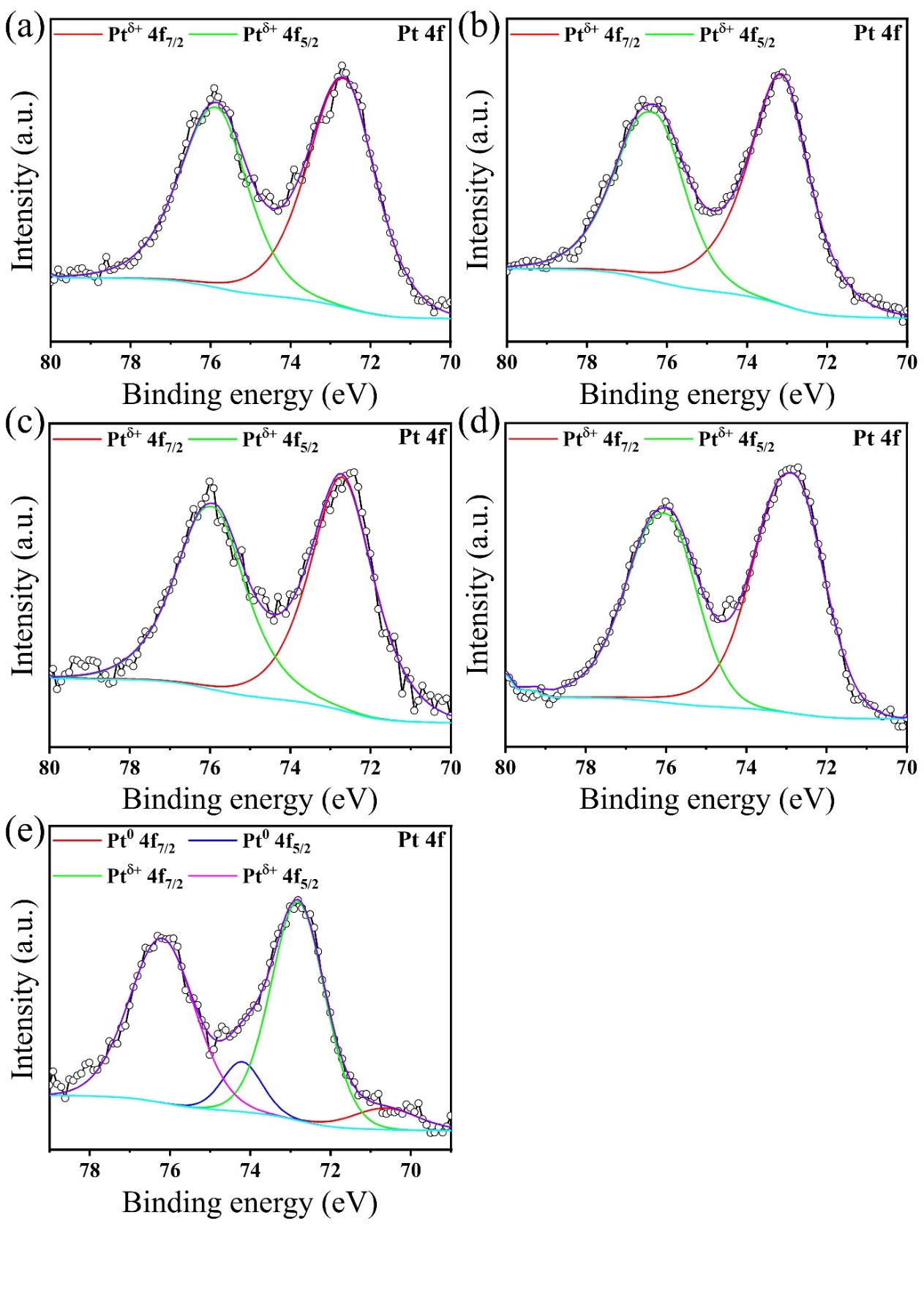


**Fig. S15** The high-resolution Pt 4f spectra of (**a**) Pt-MXene-2.5, (**b**) Pt-MXene-5, (**c**) Pt-MXene-7.5, (**d**) Pt-MXene-10 and (**e**) Pt-MXene-15 samples


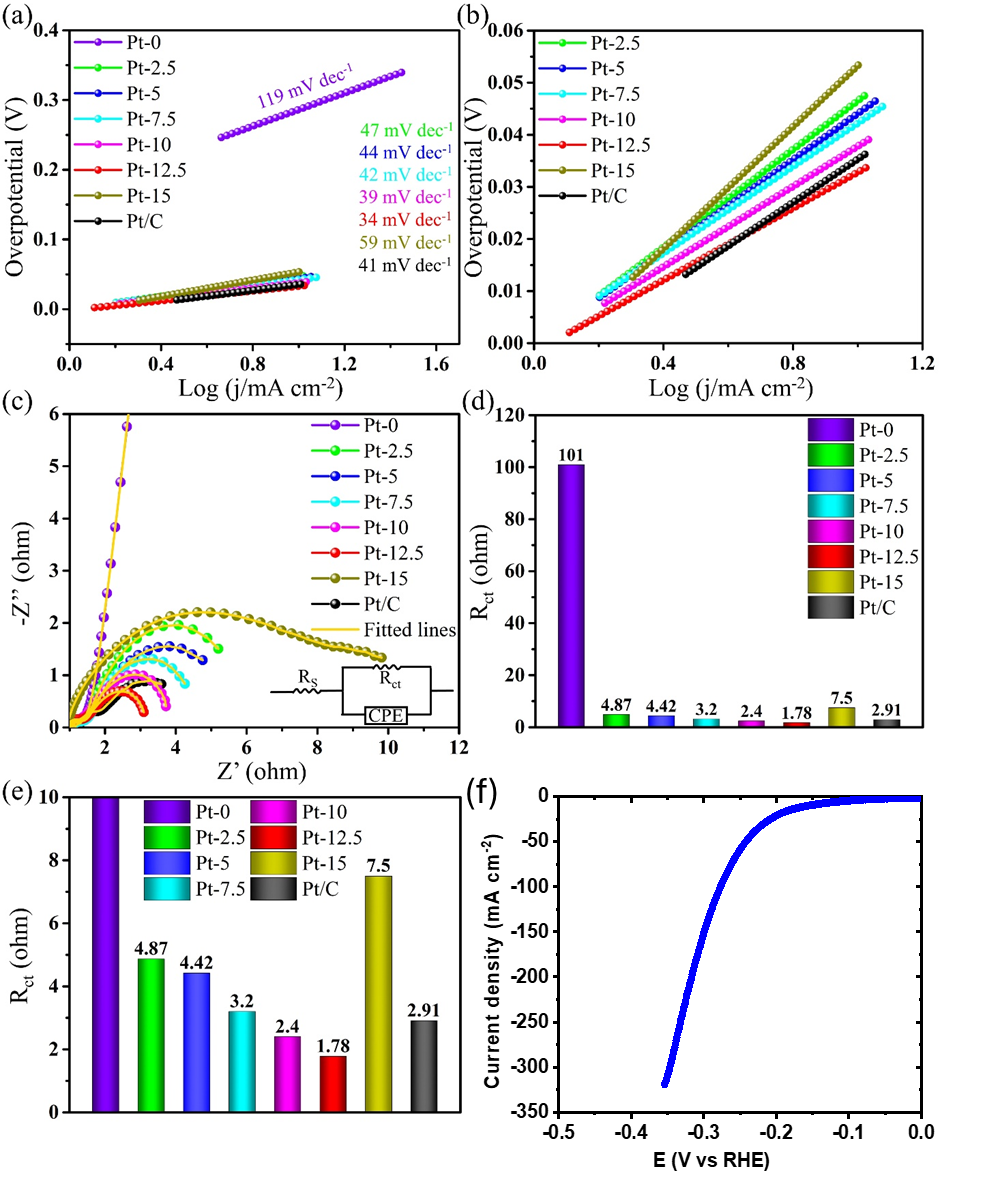


**Fig. S16** (**a**) The Tafel slopes of MXene, Pt/C and all Pt-MXene samples obtained from the HER data analyses in 1 M KOH, and (**b**) The enlarged Tafel slopes of Pt/C and all Pt-MXene samples. (**c**) EIS and the fitted curves for HER of MXene, Pt/C and all Pt-MXene samples. (**d**) The R_ct_ plots obtained from the fitted EIS results and (**e**) the enlarged R_ct_ plots of (d). (**f**) LSV curve of MXene without nitrogen in 1 M KOH


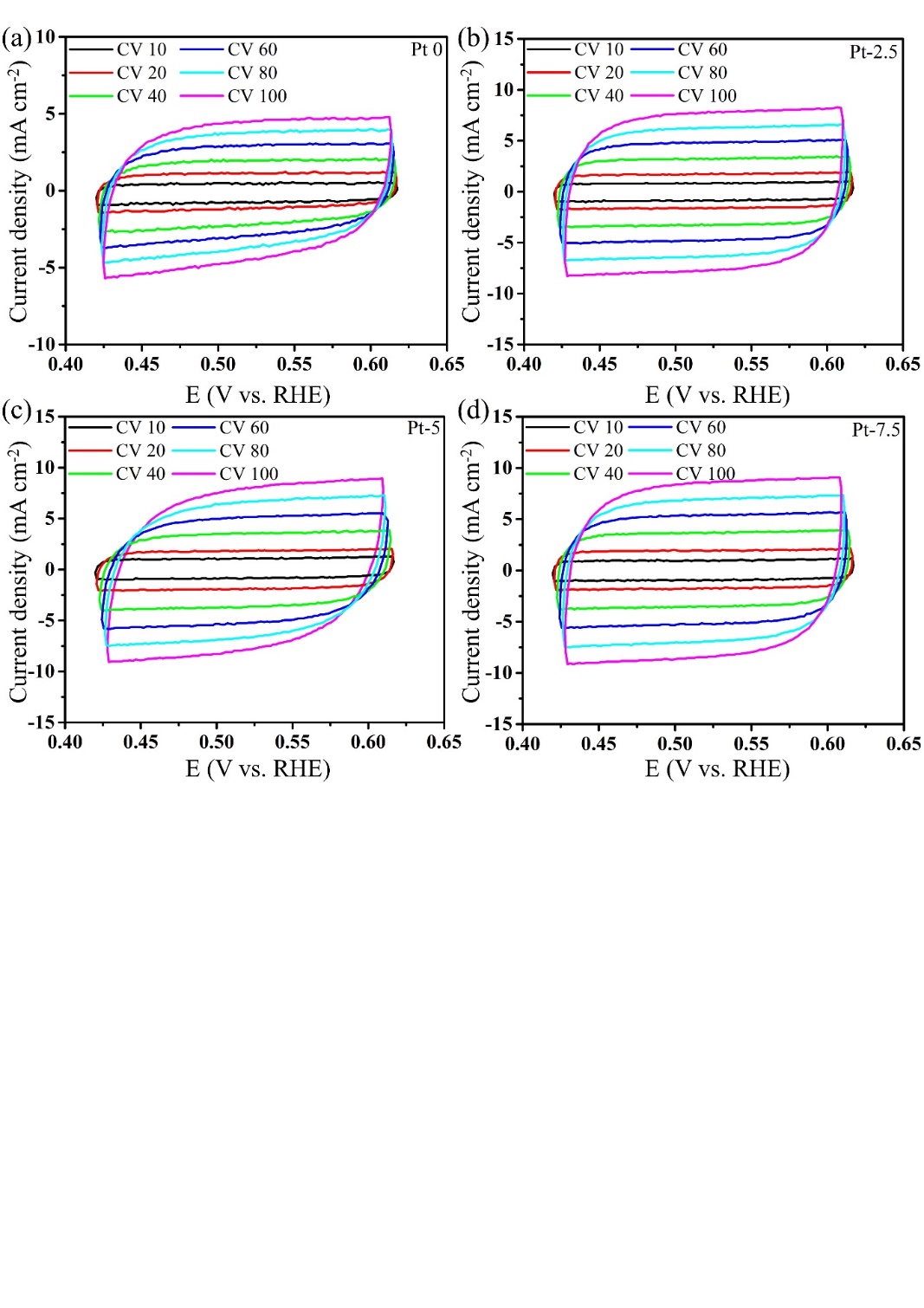


**Fig. S17** Cyclic voltammograms of (**a**) Pure MXene; (**b**) Pt-MXene-2.5; (**c**) Pt-MXene-5; and (**d**) Pt-MXene-7.5 at various scan rates (from 10 to 20, 40, 60, 80 and 100 mV s^-1^) under the potential from 0.42-0.62 V vs. RHE in 1 M KOH electrolyte


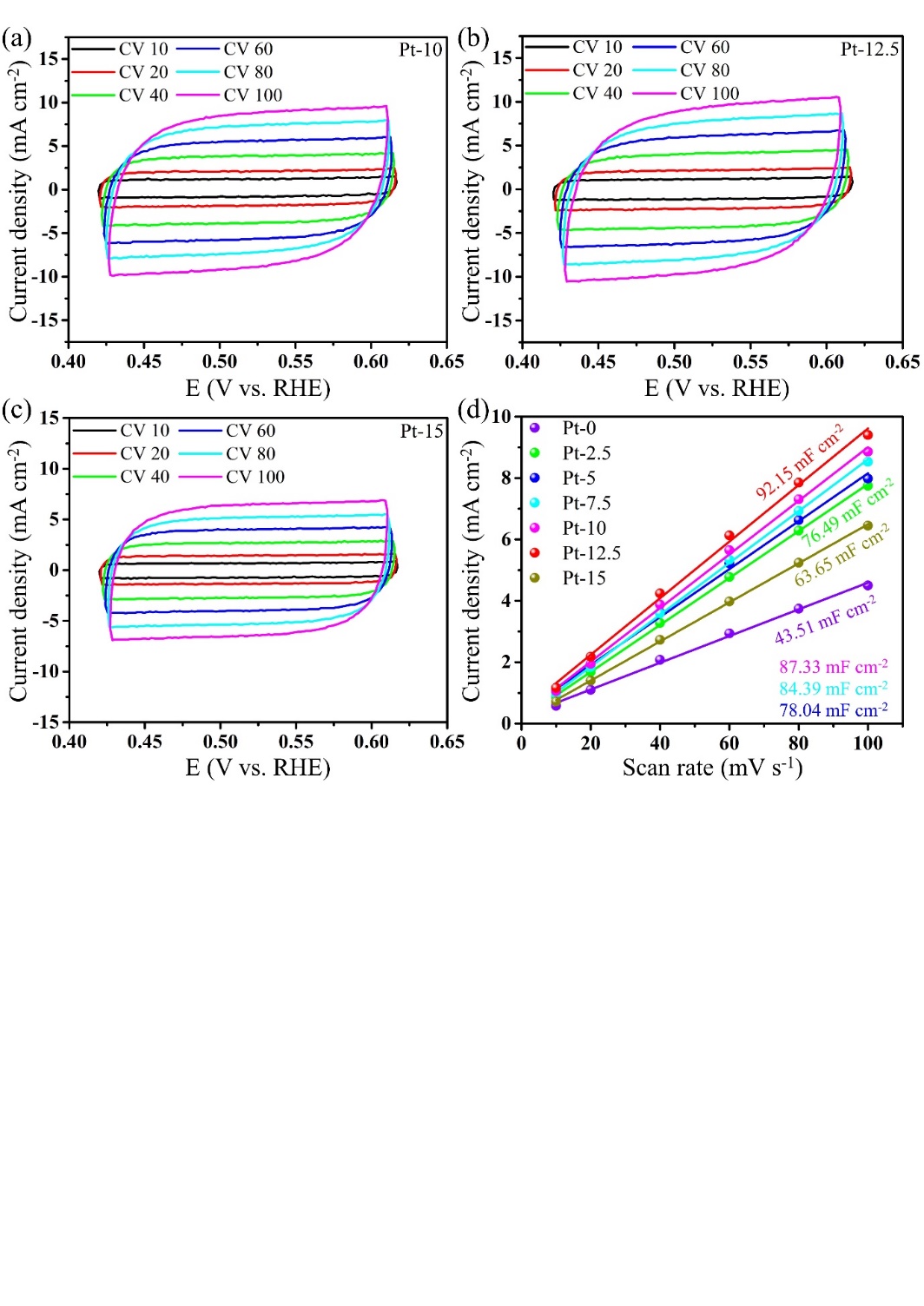


**Fig. S18** Cyclic voltammograms of (**a**) Pt-MXene-10; (**b**) Pt-MXene-12.5; (**c**) Pt-MXene-15 at various scan rate (from 10 to 20, 40, 60, 80 and 100 mV s^-1^) under the potential from 0.42-0.62 V vs. RHE in 1 M KOH electrolyte. (**d**) The corresponding linear fitting of the current density vs scan rate to calculate the double-layer capacitance (C_dl_)


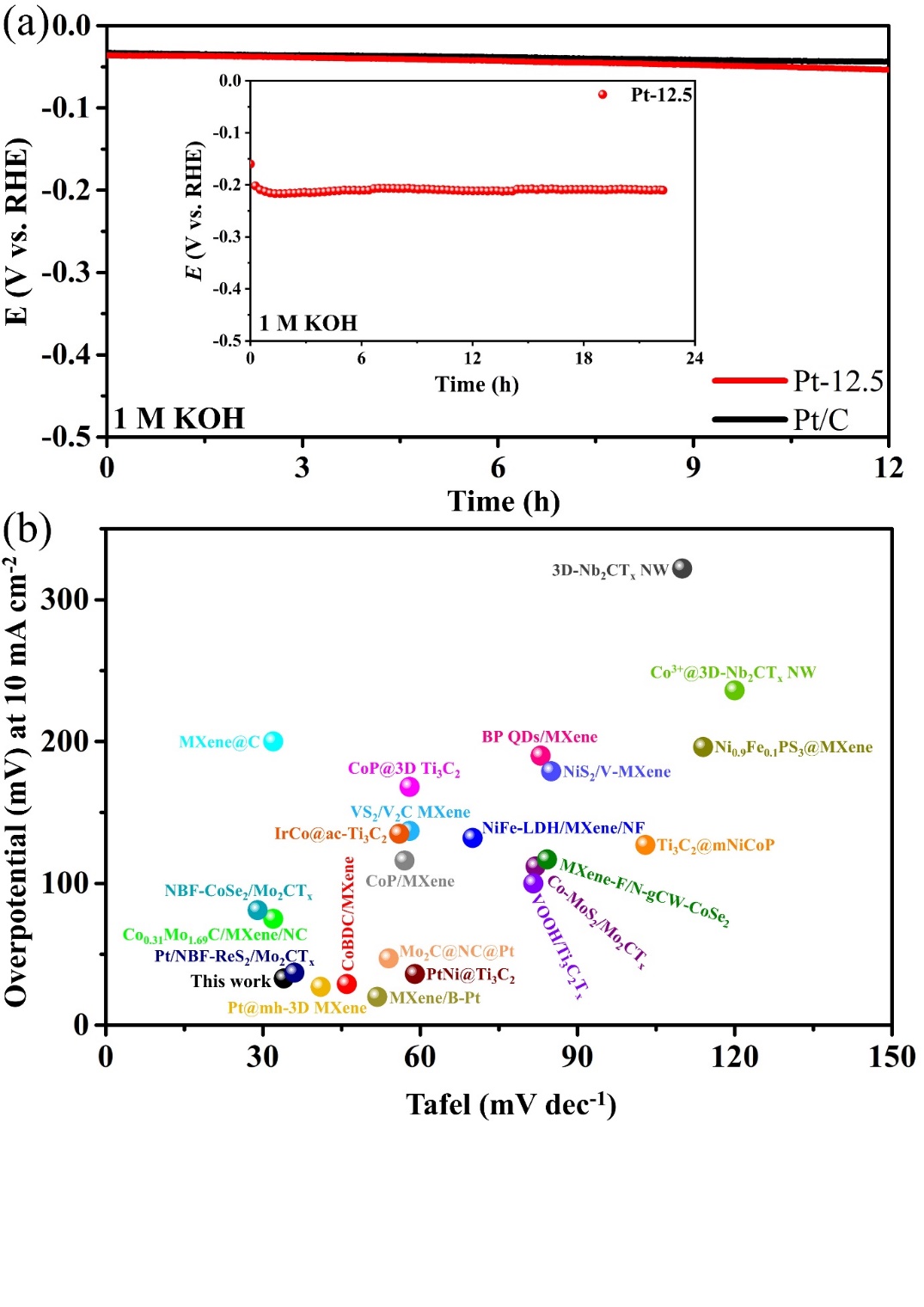


**Fig. S19** (**a**) The stability test of Pt-MXene-12.5 and Pt/C electrocatalysts at a constant current density of 10 mA cm^-2^ in 1 M KOH. The inset is the stability study with a constant current density of 200 mA cm^-2^. It is to note the peaks at 6.7 and 14.4 hr are due to the change of electrolyte. (**b**) Summary and comparison of the HER overpotentials at 10 mA cm^-2^ and the corresponding Tafel slope for Pt-MXene-12.5 and other previously reported catalyst in 1 M KOH


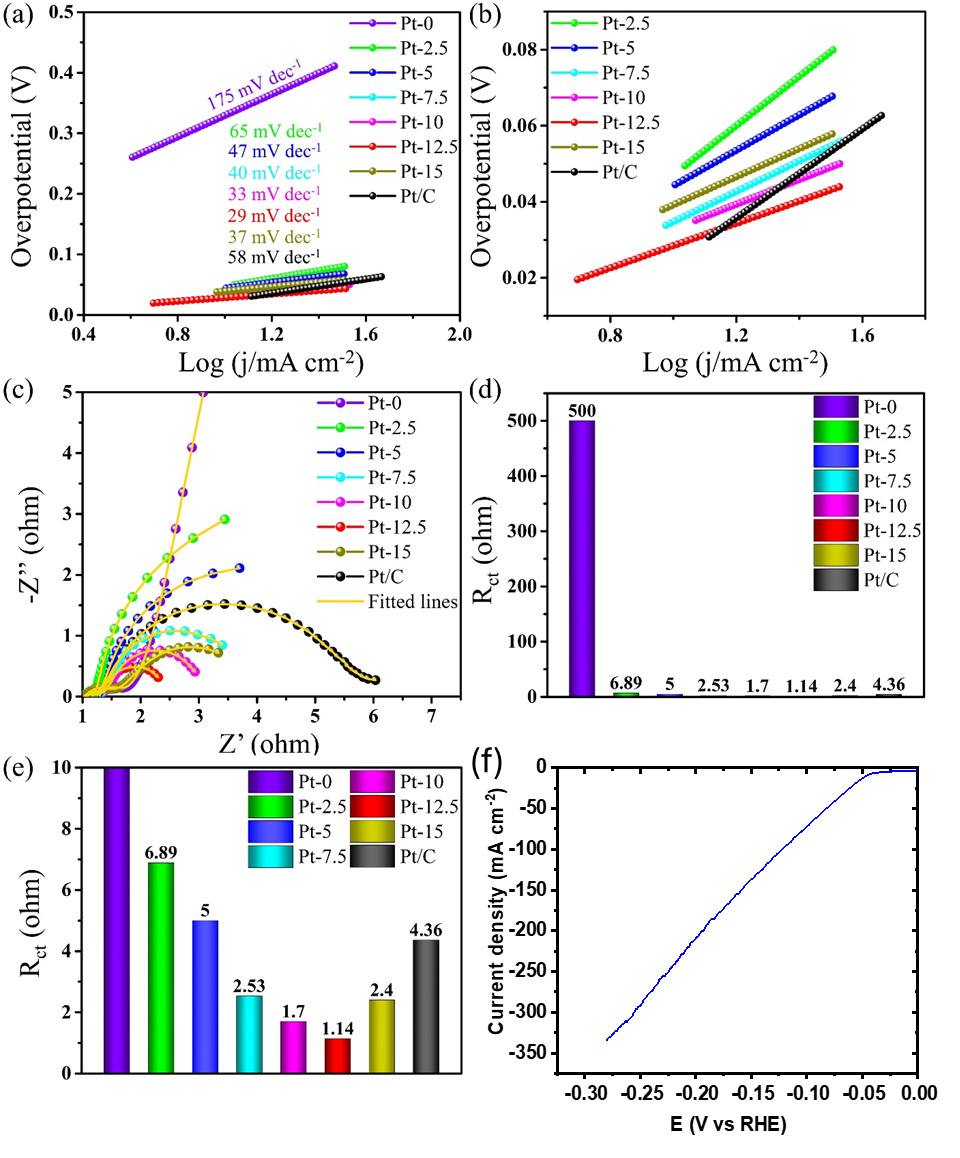


**Fig. S20** (**a**) The Tafel slopes of MXene, Pt/C and all Pt-MXene samples obtained from the HER data analysis in 0.5 M H_2_SO_4_, and (**b**) The enlarged Tafel slopes of Pt/C and all Pt-MXene samples. (**c**) EIS and fitted curves for HER of MXene, Pt/C and all Pt-MXene samples. (**d**) The corresponding Rct plots obtained from the fitted results for HER of MXene and all Pt-MXene samples, and (**e**) the enlarged Rct plots of (**d**). (**f**) LSV curve of MXene without N in 0.5 M H_2_SO_4_


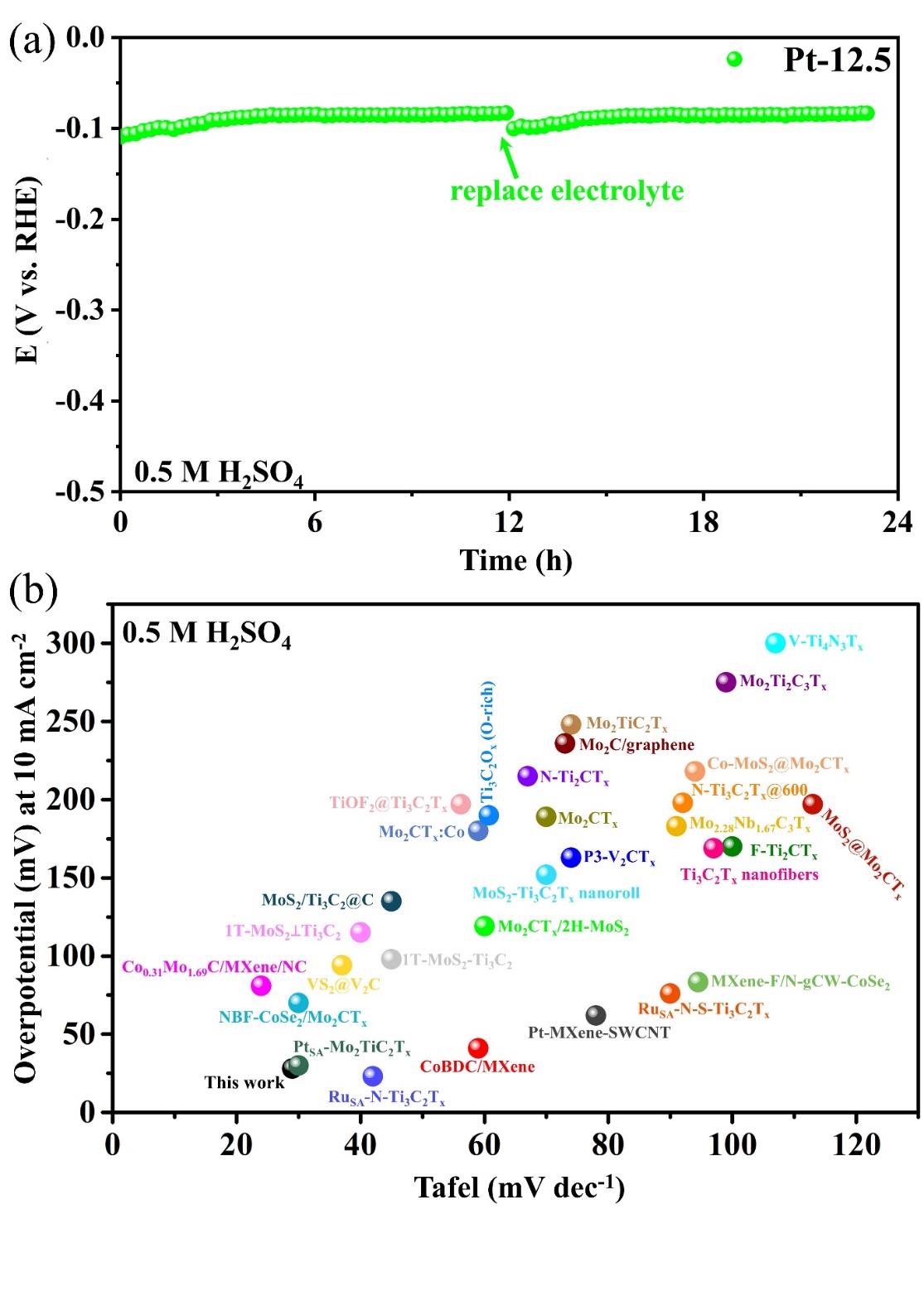


**Fig. S21** (**a**) The stability test of Pt-MXene-12.5 at a constant current density of 200 mA cm^-2^ in 0.5 M H_2_SO_4_. (**b**) Summary and comparison of the HER overpotentials at 10 mA cm^-2^ and the corresponding Tafel slope for Pt-MXene-12.5 and other previously reported catalyst in 0.5 M H_2_SO_4_


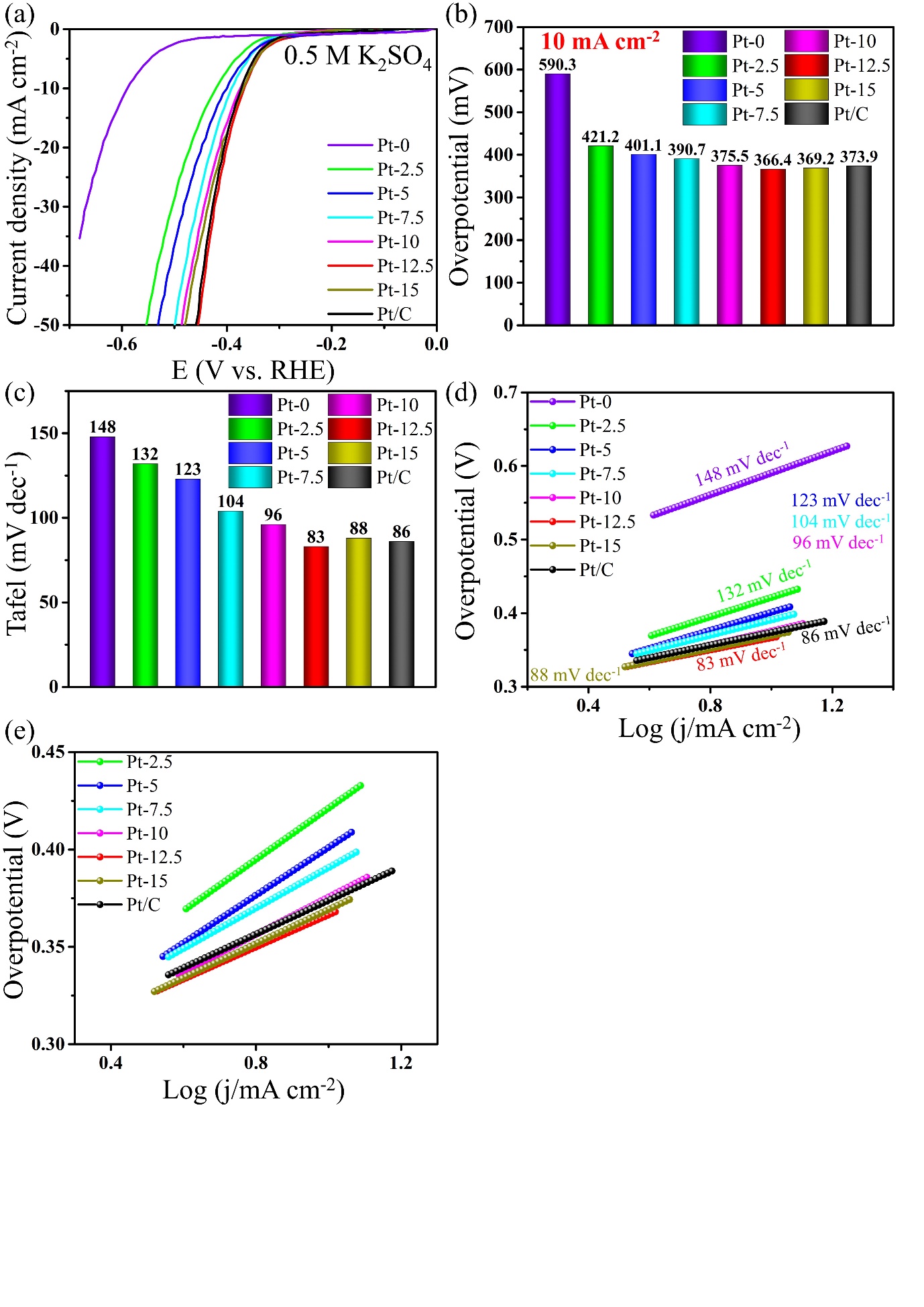


**Fig. S22** (**a**) LSV polarization curves and (**b**) the corresponding overpotential (@ 10 mA cm^-2^) plots in 0.5 M K_2_SO_4_ electrolyte for MXene, Pt/C and all Pt-MXene samples. (**c**) The Tafel slope plots for MXene, Pt/C and all Pt-MXene samples. (**d**) Tafel slopes of MXene, Pt/C and all Pt-MXene samples obtained from the HER data analysis in 0.5 M K_2_SO_4_, and (**e**) The enlarged Tafel slopes of Pt/C and all Pt-MXene samples


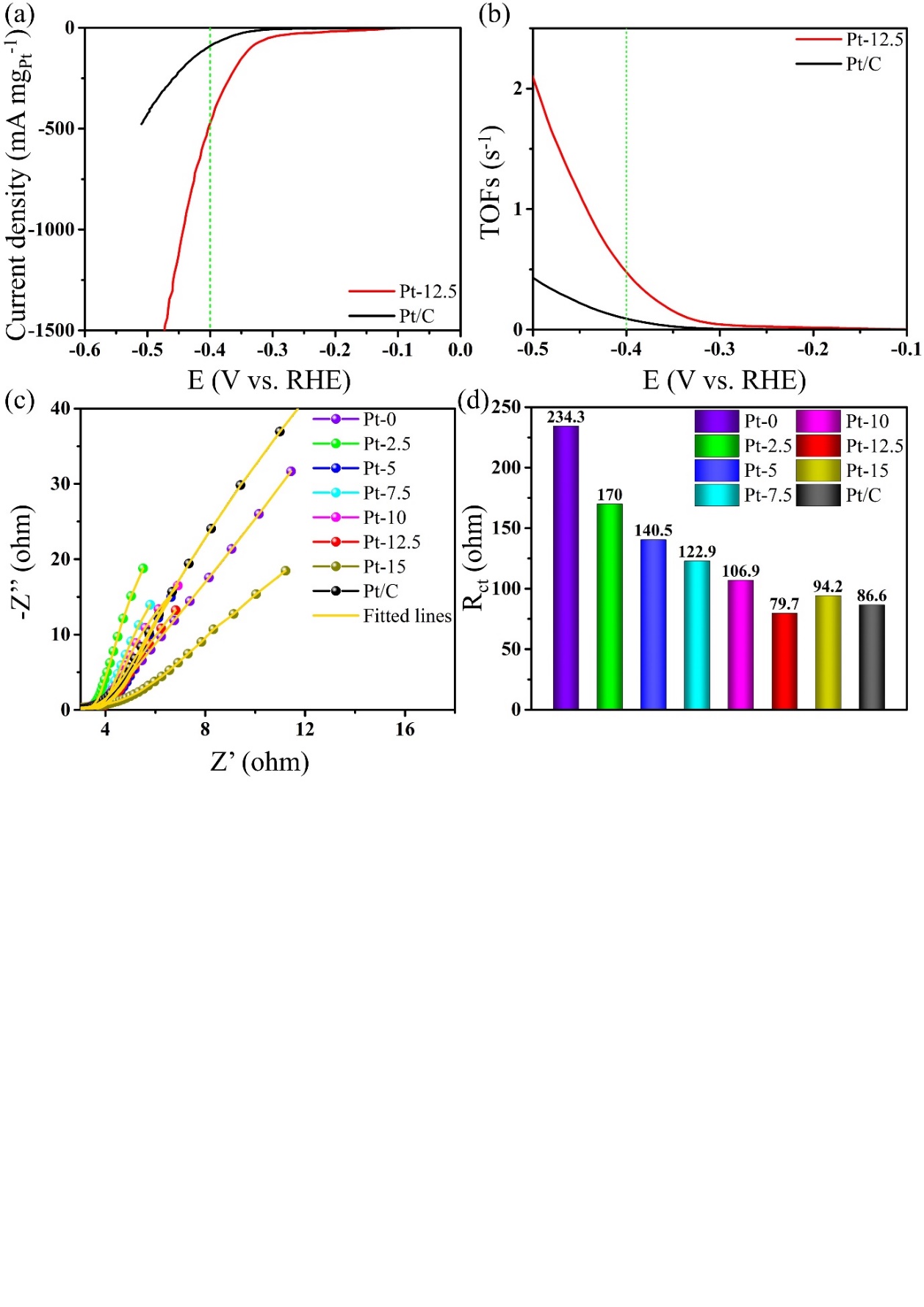


**Fig. S23** (**a**) Mass activity of Pt-MXene-12.5 and Pt/C. (**b**) TOF plots of Pt-MXene-12.5 and Pt/C. (**c**) EIS and the fitted curves for HER of MXene, Pt/C and all Pt-MXene samples. (**d**) The R_ct_ plots obtained from the fitted EIS results


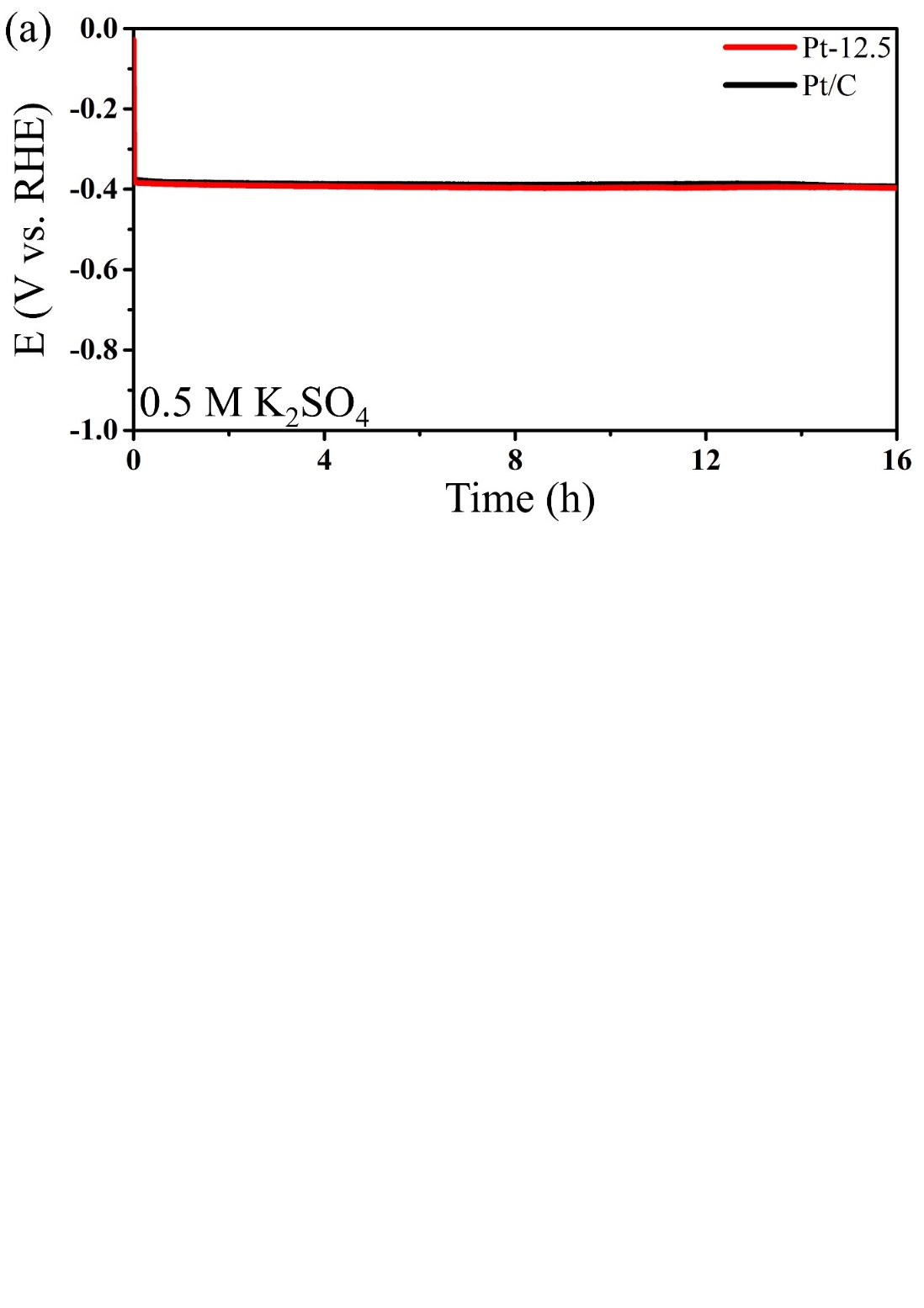


**Fig. S24** The stability test of Pt-MXene-12.5 and the commercial Pt/C electrocatalysts at a constant current density of 10 mA cm^-2^ in 0.5 M K_2_SO_4_


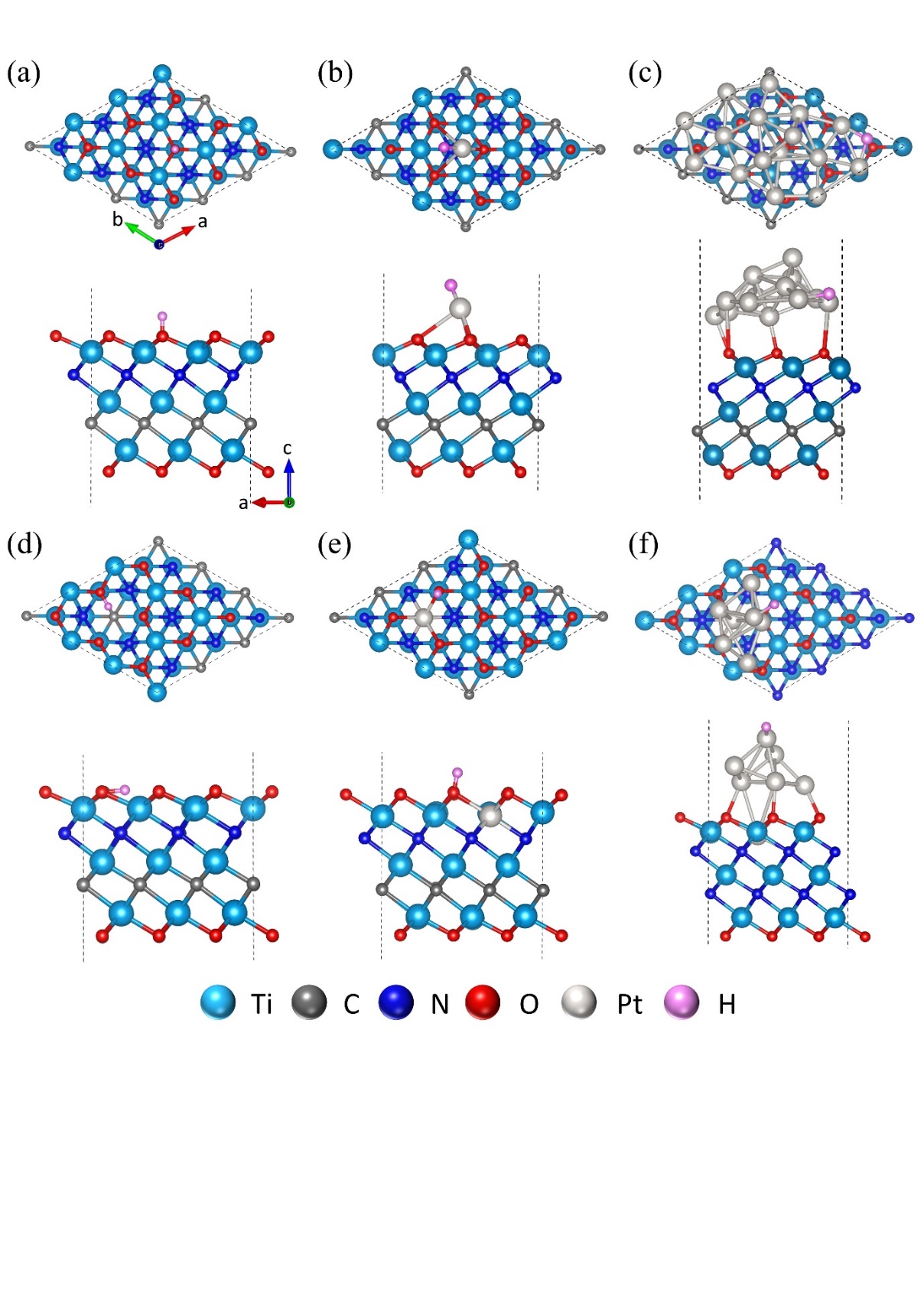


**Fig. S25** Top and side views of the structural models of (**a**) Ti_3_CNO_2_ (N) MXene, (**b**) Ti_3_CNO_2_-N-Pt_SA_, (**c**) Ti_3_CNO_2_-N-Pt_C_, (**d**) Ti_3-x_CNO_2_ (N) MXene, (e) Ti_3-x_CNO_2_-N-Pt_SA_ and (f) Ti_3-x_CNO_2_-N-Pt_C_


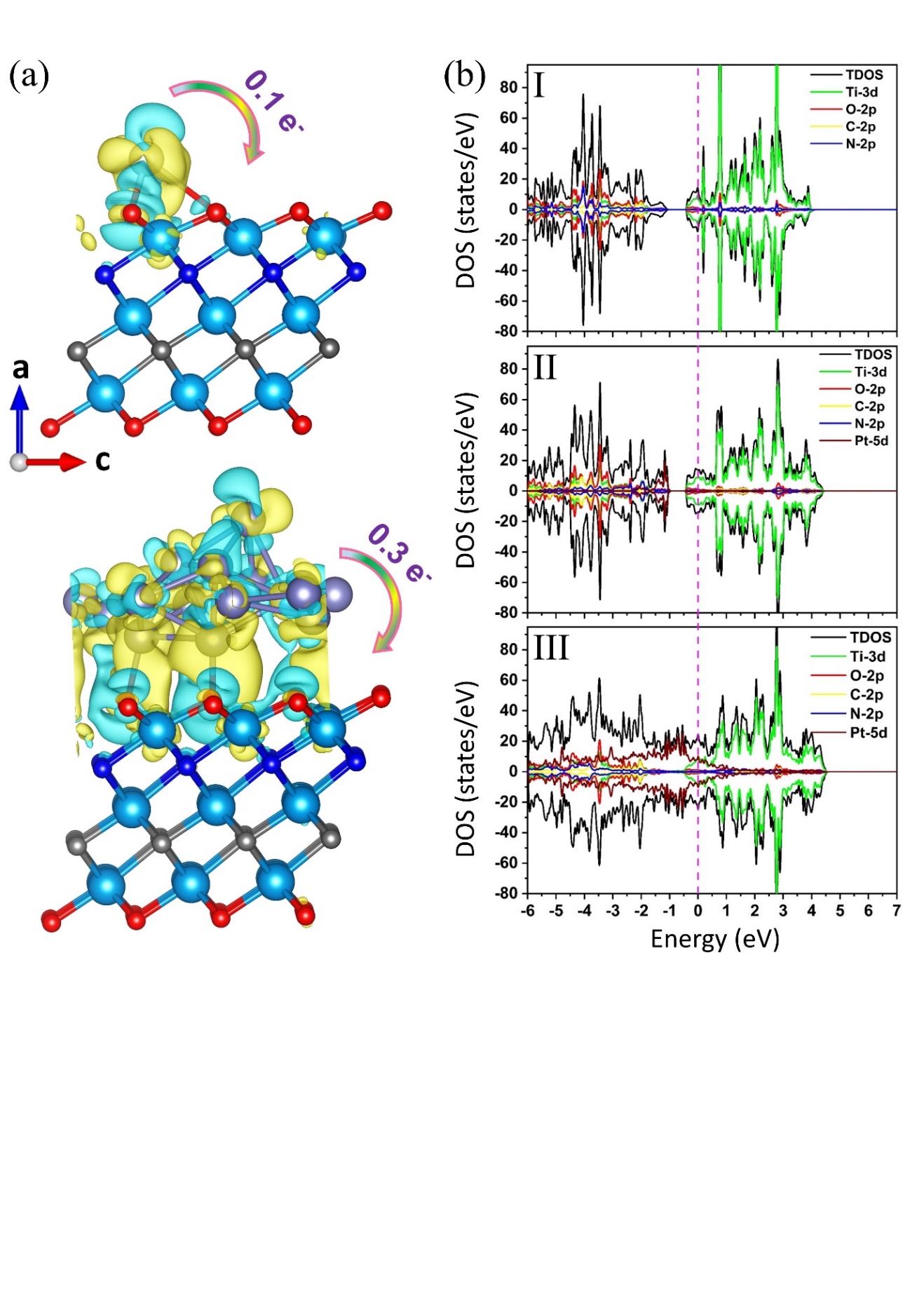


**Fig. S26** (**a**) Charge density difference plots of Ti_3_CNO_2_-N-Pt_SA_ (top) and Ti_3_CNO_2_-N-Pt_C_ (bottom). The yellow and sky-blue colour loops represent charge accumulation and depletion region, respectively. The blue, gray, dark blue, red and purple atoms represent Ti, C, N, O and Pt, respectively. (**b**) The DOS plots of Ti_3_CNO_2_ (N) MXene (I), Ti_3_CNO_2_-N-Pt_SA_ (II) and Ti_3_CNO_2_-N-Pt_C_ (III)

**
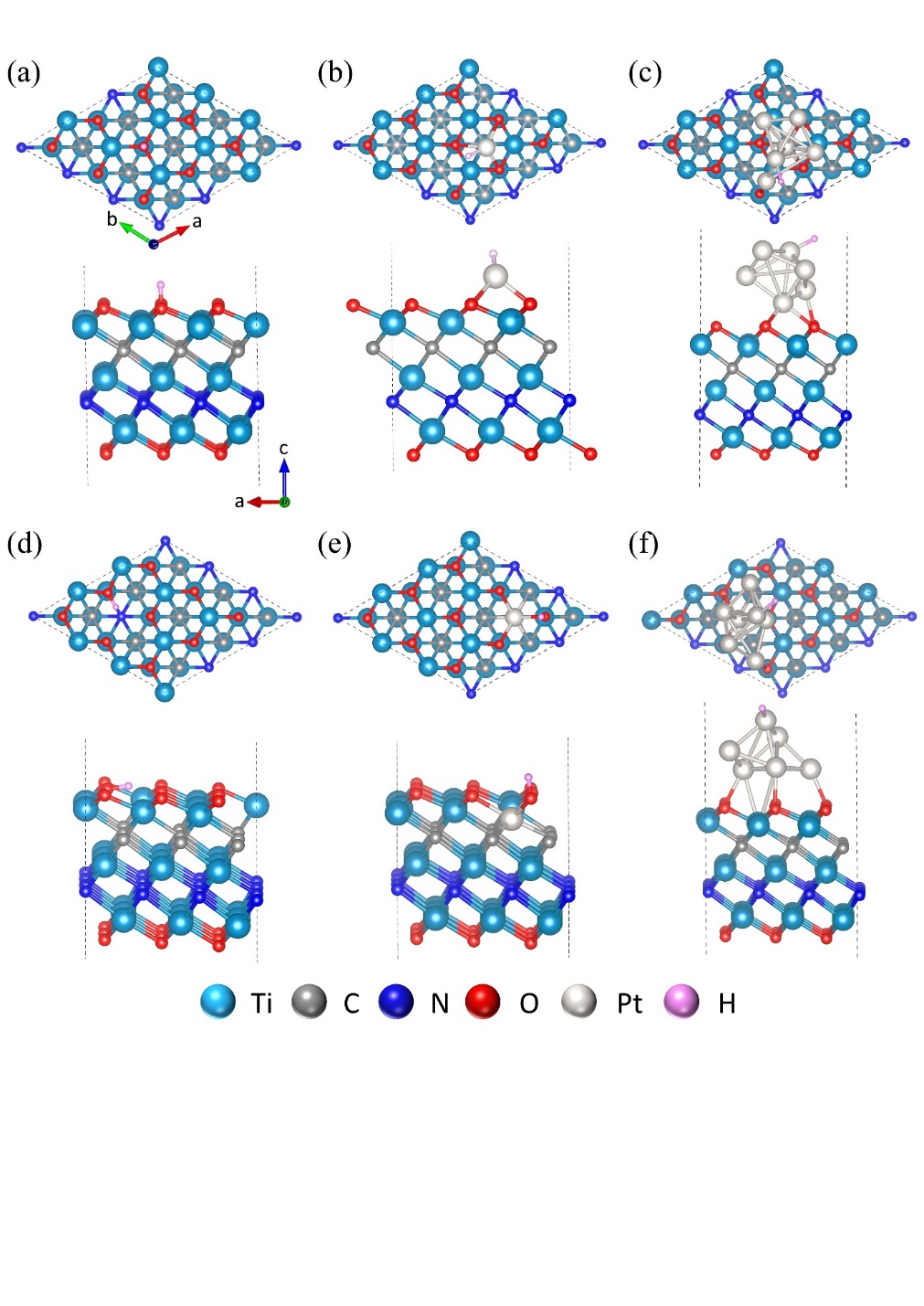
**

**Fig. S27** Top and side views of the structural models of (**a**) Ti_3_CNO_2_ (C) MXene, (**b**) Ti_3_CNO_2_-C-Pt_SA_, (**c**) Ti_3_CNO_2_-C-Pt_C_, (**d**) Ti_3-x_CNO_2_ (C) MXene, (**e**) Ti_3-x_CNO_2_-C-Pt_SA_ and (f) Ti_3-x_CNO_2_-C-Pt_C_

**
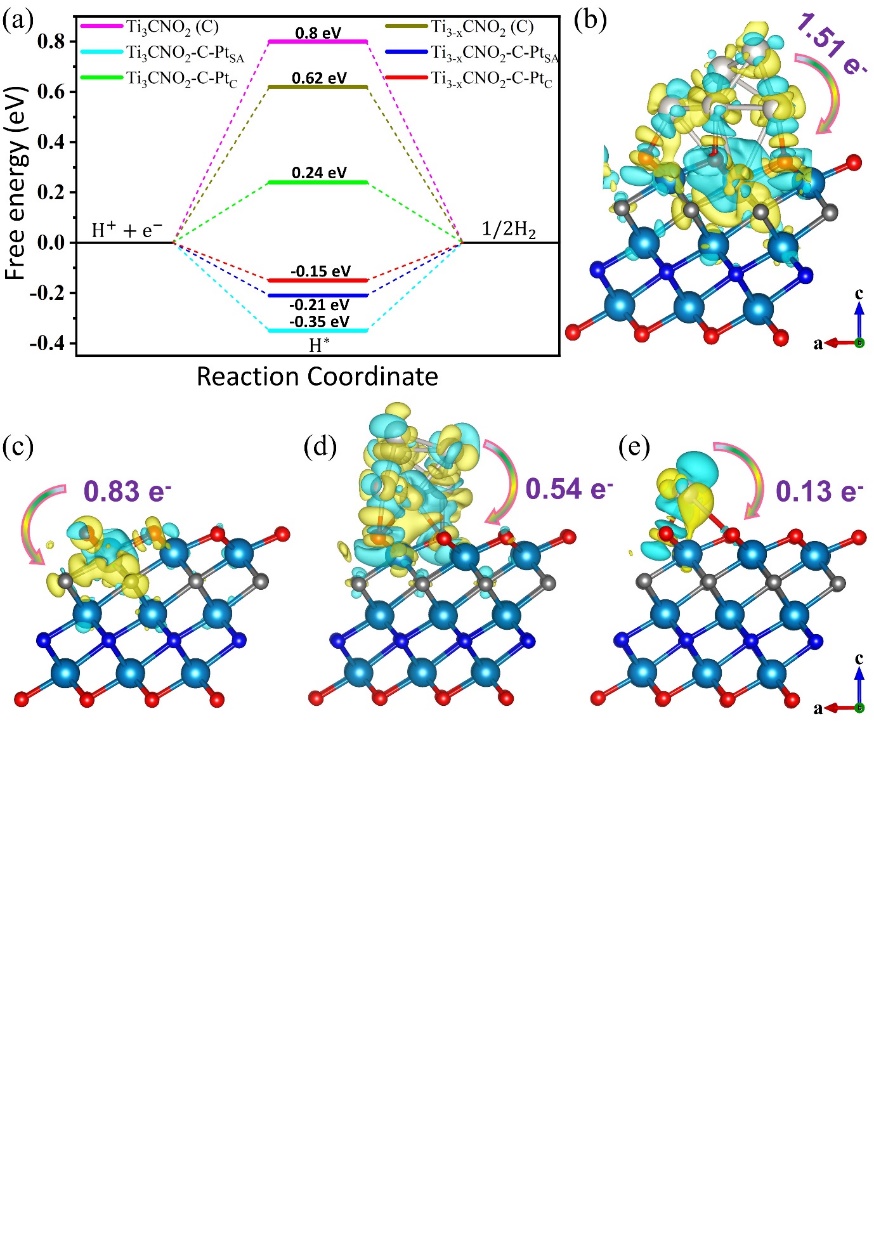
**

**Fig. S28** (**a**) The calculated free energies for hydrogen evolution on Ti_3_CNO_2_ (C) MXene, Ti_3_CNO_2_-C-Pt_SA_, Ti_3_CNO_2_-C-Pt_C_, Ti_3-x_CNO_2_ (C) MXene, Ti_3-x_CNO_2_-C-Pt_SA_ and Ti_3-x_CNO_2_-C-Pt_C_. Charge density plots of (**b**) Ti_3-x_CNO_2_-C-Pt_C_, (**c**) Ti_3-x_CNO_2_-C-Pt_SA_, (**d**) Ti_3_CNO_2_-C-Pt_C_ and (**e**) Ti_3_CNO_2_-C-Pt_SA_

**
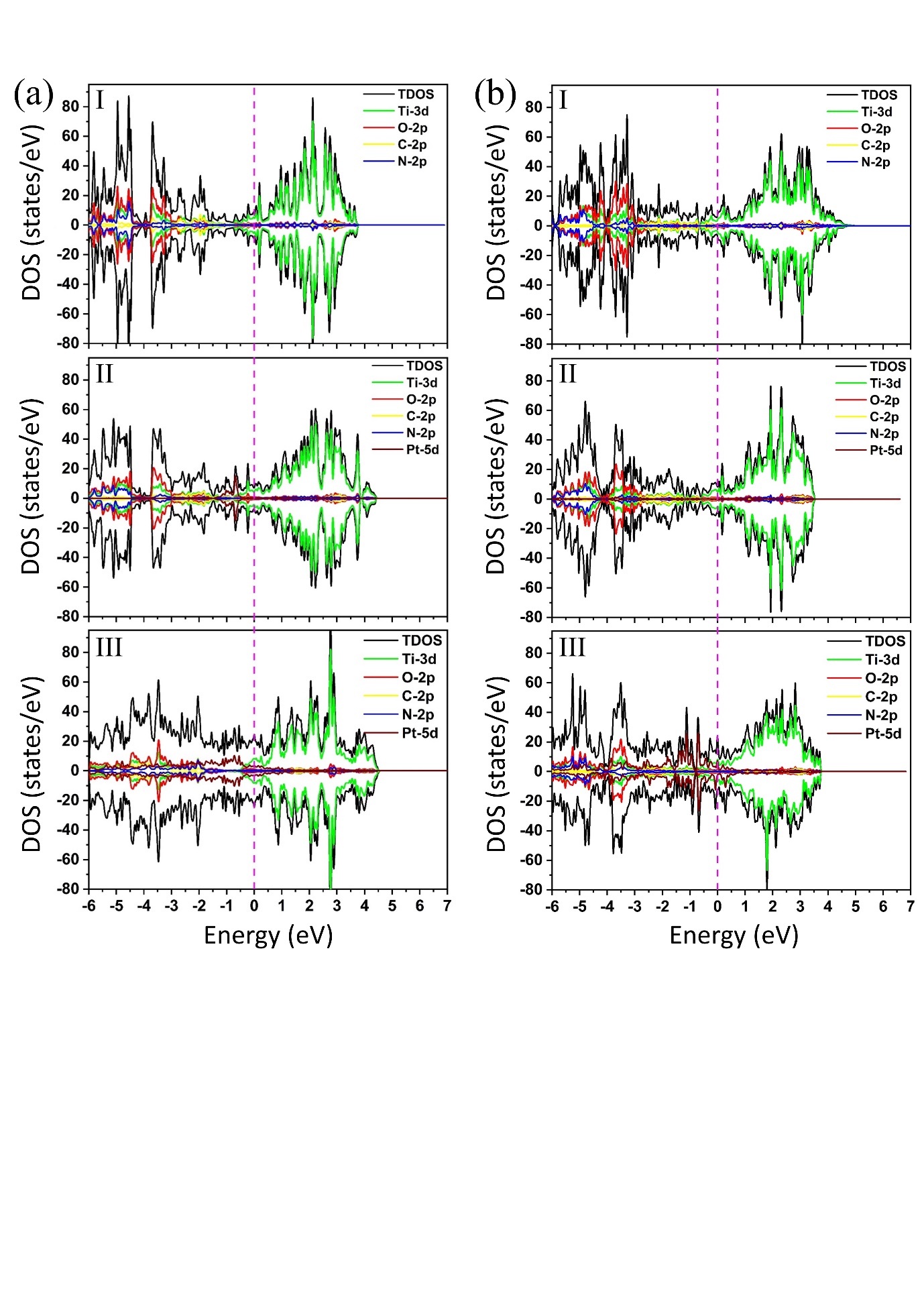
**

**Fig. S29** (**a**) The DOS plots of Ti_3_CNO_2_ (C) MXene (I), Ti_3_CNO_2_-C-Pt_SA_ (II) and Ti_3_CNO_2_-C-Pt_C_ (III). (**b**) The DOS plots of Ti_3-x_CNO_2_ (C) MXene (I), Ti_3-x_CNO_2_-C-Pt_SA_ (II) and Ti_3-x_CNO_2_-C-Pt_C_ (III)

**Table S1** Comparison of HER activity measured in this work with other representatives reported HER catalysts using 1.0 M KOH as electrolyte

| **Catalyst** | **Overpotential**  **(mV) @ 10**  **mA cm^-2^** | **Tafel slope (mV**  **dec^-1^)** | **References** |
| --- | --- | --- | --- |
| **Pt-Ti_3_CNT_x_ MXene** | **32.8** | **34** | **This work** |
| CoBDC-MXene | 29 | 46 | Adv. Funct. Mater. 2023, 33 (1), 2210322 |
| Pt@mh-3D MXene | 27 | 41 | Adv. Funct. Mater. 2020, 30 (47), 1910028. |
| Pt/NBF-ReS_2_/Mo_2_CT_x_ | 37 | 36 | Energy Storage Materials 2021, 42, 418-429. |
| MXene/B-Pt | 20 | 51.8 | J. Mater. Chem. A. 2023, 11 (11), 5830-5840. |
| PtNi@Ti_3_C_2_ | 36 | 59 | Appl. Catal. B Environ. 2021, 291, 120100. |
| Mo_2_C@NC@Pt | 47 | 57 | ACS Appl. Mater. & Inter. 2019, 11 (4), 4047-4056. |
| Co_0.31_Mo_1.69_C/MXene/NC | 75 | 32 | Adv. Energy Mater. 2019, 9 (34), 1901333. |
| NBF-CoSe_2_/Mo_2_CT_x_ | 81 | 29 | J. Energy Chem. 2022, 72, 453-464. |
| CoP/MXene | 116 | 57 | J. Mater. Chem. A. 2019, 7 (48), 27383-27393. |
| IrCo@ac-Ti_3_C_2_ | 135 | 56 | ChemSusChem 2020, 13 (5), 945-955. |
| VOOH/Ti_3_C_2_T_x_ | 100 | 81.6 | J. Mater. Chem. A. 2020, 8 (44), 23637-23644. |
| Co-MoS_2_/Mo_2_CT_x_ | 112 | 82 | Nanoscale 2019, 11 (22), 10992-11000. |
| MXene-F/N-gCW-CoSe_2_ | 116.8 | 84.2 | Carbon 2023, 206,  246-259. |
| NiFe-LDH/MXene/NF | 132 | 70 | Nano Energy 2019, 63, 103880. |
| Ti_3_C_2_@mNiCoP | 127 | 103 | ACS Appl. Mater. & Inter. 2020, 12 (16), 18570-18577. |
| VS_2_/V_2_C MXene | 137 | 58 | Nanoscale 2020, 12 (10), 6176-6187. |
| CoP@3D Ti_3_C_2_ | 168 | 58 | ACS Nano 2018, 12 (8), 8017-8028. |
| MXene@C | 200 | 32 | ACS Nano 2020, 14 (12), 17615-17625. |
| BP QDs/MXene | 190 | 83 | J. Mater. Chem. A. 2018, 6 (43), 21255-21260 |
| NiS_2_/V-MXene | 179 | 85 | J. Catal. 2019,  375, 8-20. |
| Ni_0.9_Fe_0.1_PS_3_@MXene | 196 | 114 | Adv. Energy Mater. 2018, 8 (26), 1801127. |
| Co^3+^@3D-Nb_2_CT_x_ NW | 236 | 120 | Adv. Sci. 2020, 7 (10), 1903680. |
| 3D-Nb_2_CT_x_ NW | 322 | 110 | Adv. Sci. 2020, 7 (10), 1903680. |

**Table S2** Comparison of HER activity measured in this work with other representatives reported HER catalysts using 0.5 M H_2_SO_4_ as electrolyte

| **Catalyst** | **Overpotential**  **(mV) @ 10**  **mA cm^-2^** | **Tafel slope (mV**  **dec^-1^)** | **References** |
| --- | --- | --- | --- |
| **Pt-Ti_3_CNT_x_ MXene** | **28** | **29** | **This work** |
| Pt_SA_-Mo_2_TiC_2_T_x_ | 30 | 30 | Nat. Catal. 2018, 1 (12), 985-992. |
| Ru_SA_-N-S-Ti_3_C_2_T_x_ | 76 | 90 | Adv. Mater. 2019, 31 (48), 1903841. |
| NBF-CoSe_2_/Mo_2_CT_x_ | 70 | 30 | J. Energy. Chem. 2022, 72, 453-464. |
| CoBDC-MXene | 41 | 59 | Adv. Funct. Mater. 2023, 33 (1), 2210322 |
| Pt-MXene-SWCNT | 62 | 78 | Adv. Funct. Mater. 2020, 30 (47), 2000693. |
| Co_0.31_Mo_1.69_C/MXene/NC | 81 | 24 | Adv. Energy. Mater. 2019, 9 (34), 1901333. |
| VS_2_/V_2_C MXene | 94 | 37 | Nanoscale 2020, 12 (10), 6176-6187. |
| Ru_SA_-N-Ti_3_C_2_T_x_ | 23 | 42 | J. Mater. Chem. A. 2020, 8 (46), 24710-24717. |
| 1T-MoS_2_-Ti_3_C_2_ | 98 | 45 | Appl. Catal. B Environ. 2021, 284, 119708. |
| MXene-F/N-gCW-CoSe_2_ | 83.3 | 94.5 | Carbon 2023, 206,  246-259. |
| Mo_2_CT_x_/2H-MoS_2_ | 119 | 60 | ACS Nano 2020, 14 (11), 16140-16155. |
| MoS_2_-Ti_3_C_2_@C | 135 | 45 | Adv. Mater. 2017, 29 (24), 1607017. |
| MoS_2_-Ti_3_C_2_T_x_ nanoroll | 152 | 70 | Appl. Catal. B Environ. 2019, 241, 89-94. |
| Mo_2_CTx:Co | 180 | 59 | J. Am. Chem. Soc. 2019, 141 (44), 17809-17816. |
| TiOF_2_@Ti_3_C_2_T_x_ | 197 | 56.2 | Appl. Surf. Sci. 2019, 496, 143729. |
| P3-V_2_CT_x_ | 163 | 74 | Adv. Funct. Mater. 2019, 29 (30), 1903443. |
| Mo_2_CT_x_ | 189 | 70 | ACS Energy. Lett. 2016, 1 (3), 589-594. |
| N-Ti_2_CT_x_ | 215 | 67 | J. Mater. Chem. A. 2018, 6 (42), 20869-20877. |
| Mo_2_C/graphene | 236 | 73 | Adv. Mater. 2017, 29 (35), 1700072. |
| Mo_2_TiC_2_T_x_ | 248 | 74 | ACS Appl. Energy. Mater. 2018, 1 (1), 173-180. |
| Ti_3_C_2_T_x_ nanofibers | 169 | 97 | ACS Sustain. Chem. & Eng. 2018, 6 (7), 8976-8982. |
| F-Ti_2_CT_x_ | 170 | 100 | Nano Energy 2018, 47,  512-518. |
| Mo_2.28_Nb_1.67_C_3_T_x_ | 183 | 91 | Nano Letters 2023, 23 (3), 931-938. |
| N-Ti_3_C_2_T_x_@600 | 198 | 92 | ACS Sustain. Chem. & Eng. 2019, 7 (19), 16879-16888. |
| Co-MoS_2_@Mo_2_CT_x_ | 218 | 94 | Nanoscale 2019, 11 (22), 10992-11000. |
| Mo_2_Ti_2_C_3_T_x_ | 275 | 99 | ACS Appl. Energy. Mater. 2018, 1 (1), 173-180. |
| MoS_2_@Mo_2_CT_x_ | 197 | 113 | CrystEngComm 2020,  22 (8), 1395-1403. |
| V-Ti_4_N_3_T_x_ | 300 | 107 | Adv. Funct. Mater. 2020, 30 (47), 2001136. |
| 1T-MoS_2_$\perp$Ti_3_C_2_ | 115 | 40 | J. Mater. Chem. A. 2018, 6 (35), 16882-16889. |

**Table S3** Summary of the DFT calculations and details of the constructed twelve different models, including the absence/presence of Ti vacancies (V_Ti_), the anchored different Pt species, the different Pt bonding states, the calculated charge transfer between Pt species and MXene support, and free energies for hydrogen evolution reaction

| **Theoretical**  **models** | **V_Ti_** | **Pt species & the bonding states** | **Charge**  **Transfer (e^-^)** | **Free**  **energies (eV)** |
| --- | --- | --- | --- | --- |
| Ti_3_CNO_2_ (N side) | NO | Absence | 0 | -0.46 |
| Ti_3_CNO_2_-N-Pt_SA_ | NO | Pt single atoms/ Pt bonded with surface O atoms | 0.1 | -0.31 |
| Ti_3_CNO_2_-N-Pt_C_ | NO | Pt clusters/ Pt bonded with surface O atoms | 0.3 | -0.21 |
| Ti_3-x_CNO_2_ (N side) | YES | Absence | 0 | 0.4 |
| Ti_3-x_CNO_2_-N-Pt_SA_ | YES | Pt single atoms/ Pt bonded with N and O atoms | 1.2 | -0.25 |
| **Ti_3-x_CNO_2_-N-Pt_C_** | **YES** | **Pt clusters/ Pt bonded with N and O atoms** | **1.56** | **-0.11** |
| Ti_3_CNO_2_ (C side) | NO | Absence | 0 | 0.8 |
| Ti_3_CNO_2_-C-Pt_SA_ | NO | Pt single atoms/ Pt bonded with surface O atoms | 0.13 | -0.35 |
| Ti_3_CNO_2_-C-Pt_C_ | NO | Pt clusters/ Pt bonded with surface O atoms | 0.54 | 0.24 |
| Ti_3-x_CNO_2_ (C side) | YES | Absence | 0 | 0.62 |
| Ti_3-x_CNO_2_-C-Pt_SA_ | YES | Pt single atoms/ Pt bonded with C and O atoms | 0.83 | -0.21 |
| Ti_3-x_CNO_2_-C-Pt_C_ | YES | Pt clusters/ Pt bonded with C and O atoms | 1.51 | -0.15 |

**Supplementary References**

1. G. Kresse, J. Furthmüller, Efficiency of ab-initio total energy calculations for metals and semiconductors using a plane-wave basis set. Comput. Mater. Sci. **6**, 15–50 (1996). <https://doi.org/10.1016/0927-0256(96)00008-0>
2. G. Kresse, J. Furthmüller, Efficient iterative schemes for *ab initio* total-energy calculations using a plane-wave basis set. Phys. Rev. B Condens. Matter **54**, 11169–11186 (1996). <https://doi.org/10.1103/physrevb.54.11169>
3. J.P. Perdew, A. Ruzsinszky, G.I. Csonka, O.A. Vydrov, G.E. Scuseria et al., Restoring the density-gradient expansion for exchange in solids and surfaces. Phys. Rev. Lett. **100**, 136406 (2008). <https://doi.org/10.1103/PhysRevLett.100.136406>
4. S. Grimme, J. Antony, S. Ehrlich, H. Krieg, A consistent and accurate *ab initio* parametrization of density functional dispersion correction (DFT-D) for the 94 elements H-Pu. J. Chem. Phys. **132**, 154104 (2010). <https://doi.org/10.1063/1.3382344>
5. J.K. Nørskov, J. Rossmeisl, A. Logadottir, L. Lindqvist, J.R. Kitchin et al., Origin of the overpotential for oxygen reduction at a fuel-cell cathode. J. Phys. Chem. B **108**, 17886–17892 (2004). <https://doi.org/10.1021/jp047349j>
